# Pangenome mining of the *Streptomyces* genus redefines species’ biosynthetic potential

**DOI:** 10.1186/s13059-024-03471-9

**Published:** 2025-01-14

**Authors:** Omkar S. Mohite, Tue S. Jørgensen, Thomas J. Booth, Pep Charusanti, Patrick V. Phaneuf, Tilmann Weber, Bernhard O. Palsson

**Affiliations:** 1 The Novo Nordisk Foundation Center for Biosustainability, Technical University of Denmark, Kongens Lyngby, 2800 Denmark; 2 Department of Bioengineering, University of California San Diego, La Jolla, CA 92093 USA; 3 Bioinformatics and Systems Biology Program, University of California San Diego, La Jolla, CA 92093 USA; 4 Department of Pediatrics, University of California San Diego, La Jolla, CA 92093 USA

**Keywords:** Pangenome analysis, *Streptomyces*, Genome mining, Biosynthetic Gene Clusters, Phylogenetic analysis, Metabolism

## Abstract

**Background:**

*Streptomyces* is a highly diverse genus known for the production of secondary or specialized metabolites with a wide range of applications in the medical and agricultural industries. Several thousand complete or nearly complete *Streptomyces* genome sequences are now available, affording the opportunity to deeply investigate the biosynthetic potential within these organisms and to advance natural product discovery initiatives.

**Results:**

We perform pangenome analysis on 2371 *Streptomyces* genomes, including approximately 1200 complete assemblies. Employing a data-driven approach based on genome similarities, the *Streptomyces* genus was classified into 7 primary and 42 secondary Mash-clusters, forming the basis for comprehensive pangenome mining. A refined workflow for grouping biosynthetic gene clusters (BGCs) redefines their diversity across different Mash-clusters. This workflow also reassigns 2729 known BGC families to only 440 families, a reduction caused by inaccuracies in BGC boundary detections. When the genomic location of BGCs is included in the analysis, a conserved genomic structure, or synteny, among BGCs becomes apparent within species and Mash-clusters. This synteny suggests that vertical inheritance is a major factor in the diversification of BGCs.

**Conclusions:**

Our analysis of a genomic dataset at a scale of thousands of genomes refines predictions of BGC diversity using Mash-clusters as a basis for pangenome analysis. The observed conservation in the order of BGCs’ genomic locations shows that the BGCs are vertically inherited. The presented workflow and the in-depth analysis pave the way for large-scale pangenome investigations and enhance our understanding of the biosynthetic potential of the *Streptomyces* genus.

**Supplementary Information:**

The online version contains supplementary material available at 10.1186/s13059-024-03471-9.

## Background

*Streptomyces*, a genus of soil bacteria, is known for its ability to produce various natural products that have applications in medicine and biotechnology. These organisms are characterized by their complex and diverse biosynthetic gene clusters (BGCs), which are responsible for the biosynthesis of these bioactive compounds [1, 2]. Over the past decades, several genomic studies have revealed that the full range of metabolites produced by *Streptomyces* and the associated biosynthetic pathways are not yet fully known [3–5].

The same genomic studies have revealed extensive genomic diversity within the *Streptomyces* genus. This diversity provides a huge potential for natural product discovery but at the same time complicates comparative analyses across different species and strains [6]. To mitigate this challenge, there is a growing consensus for the need to cluster *Streptomyces* into distinct smaller groups of closely related species [7, 8]. Such refined classification aims to facilitate more precise comparisons to understand the biosynthetic diversity and evolution within the genus.

Recent advances in sequencing technology and genome mining tools have allowed for the data-driven discovery of natural products [9]. Several genome mining and BGC clustering tools such as antiSMASH, BiG-SCAPE, and BiG-SLICE are available to assess the biosynthetic potential encoded in the genomes [10–13]. The genome mining tools have revealed that various bacterial species encode previously unknown biosynthetic potential [8, 14, 15]. While genome mining tools have significantly advanced our understanding of biosynthetic potential, there is a recognition that the estimates of diversity and novelty can be constrained by the inherent limitations of these individual tools and reference databases. These limitations include inaccurate definitions of BGC boundaries or incomplete entries in reference databases such as MIBiG [16]. The strategy of integrating results from different tools can partially mitigate these challenges [17].

Large-scale pangenome mining studies help to understand the evolutionary patterns of biosynthetic gene clusters (BGCs) along with a deep characterization of the biosynthetic repertoire of a given bacterial species or genus [8, 14, 15, 18]. Earlier pangenomic investigations of *Streptomyces*, examining 121 genomes [19], 124 genomes [20], and 205 genomes [18], respectively, have underscored that the pangenome of *Streptomyces* is open (a pangenome is said to be open if newly sequenced genomes appear to keep adding novel genes [21]) and highly diverse. These analyses brought to light a limited number of core genes—633 [19], 1018 [20], and 304 [18]—found across all strains considered in each study, respectively. Recent sequencing efforts have significantly increased the publicly available high-quality genomes of *Streptomyces* [22]. In light of this explosion of sequencing data, there is an emerging need to re-investigate the *Streptomyces* pangenome and the biosynthetic diversity within these organisms.

Recently, there has been an increase in using whole genome similarity as a basis for grouping taxa. For example, a Mash-based approach revealed 14 distinct phylogroups of *Escherichia coli* species [23] and a genome similarity network of *Pseudomonas* genus revealed at least 14 divisions [24]. Tools like Mash [25] and FastANI [26] can be used to find the genome similarities with similar performance. Here, we use Mash to group the highly diverse members of the *Streptomyces* genus. Furthermore, we show that Mash-clusters can be defined in a hierarchical fashion at primary and secondary levels depending on the size of each cluster. This classification provides an objective metric for defining groups based on genome similarity that is not reliant on species definitions, which are often contentious in *Streptomyces*.

This study constitutes the largest pangenome mining study of *Streptomyces* to date. By combining insights from the Mash-clustering with various genome mining tools, we have updated our understanding of the biosynthetic potential in this diverse genus.

## Results

### The dataset of *Streptomyces* genomes

**Fig. 1:**
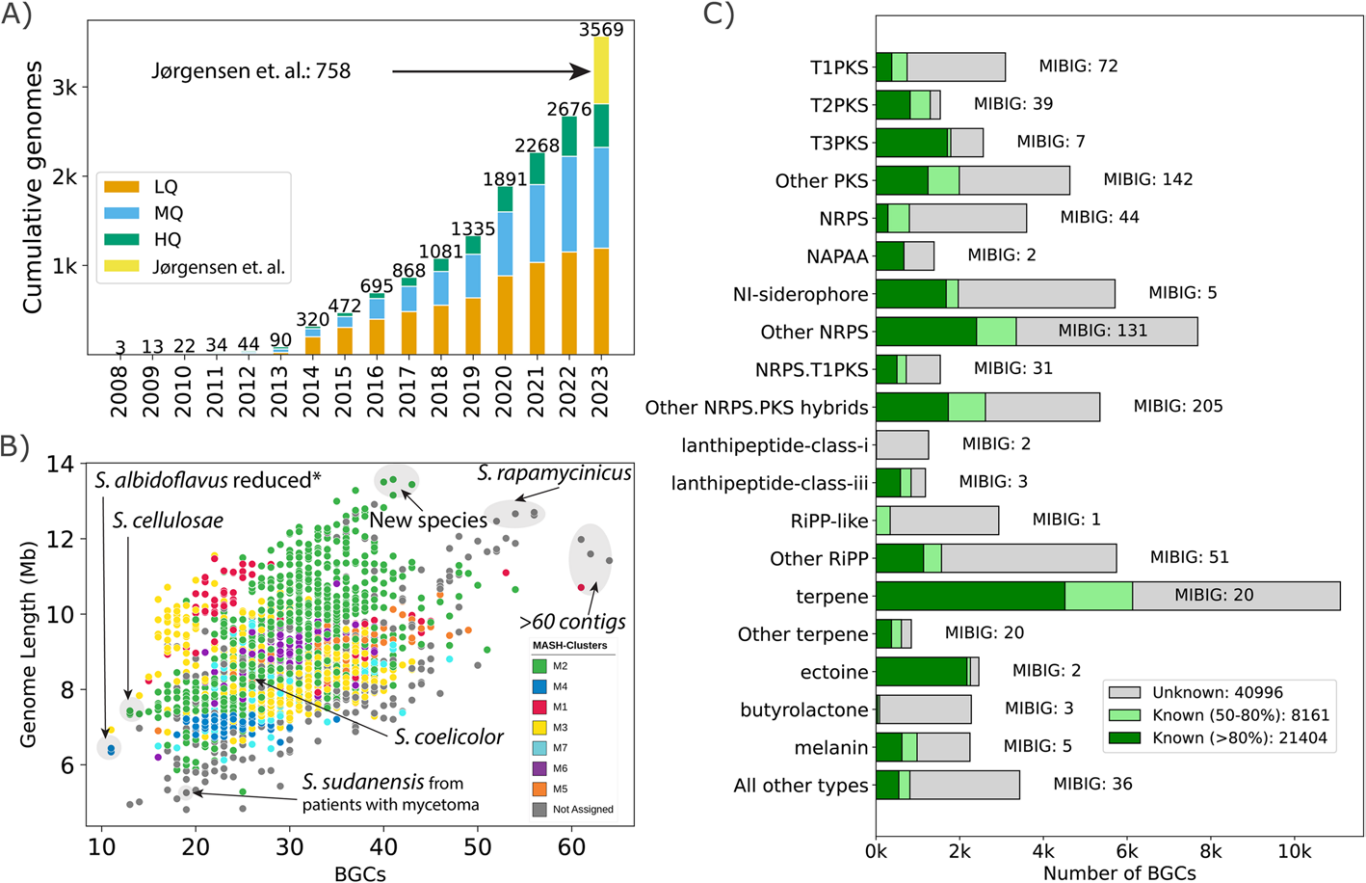
Dataset of *Streptomyces* genomes and BGC statistics. **A** Number of *Streptomyces* genomes from the NCBI RefSeq database as of 30 June 2023. The final bar includes newly sequenced high-quality genomes from our recent study [22]. Genomes are categorized by assembly quality: HQ (high-quality), MQ (medium-quality), and LQ (low-quality). **B** Scatter plot illustrating the relationship between genome length and the number of BGCs in 2371 genomes of the MQ and HQ categories. Annotations represent information on selected strains. **C** Breakdown of the common types of BGCs detected in the HQ and MQ genomes. Color-coded bars highlight BGC similarity percentages against the MIBiG database: gray for < 50%, light green for 50–80%, and green for > 80%. Bar annotations represent a tally of MIBiG entries with > 80% similarity for the detected BGCs. Abbreviation of BGC types: T1PKS, type 1 polyketide synthase; T2PKS, type 2 polyketide synthase; T3PKS, type 3 polyketide synthase; Other PKS, hybrid BGCs with at least one PKS category BGC; NRPS, non-ribosomal peptide synthetase; NAPAA, non-alpha poly-amino acids like ε-polylysine; NI-siderophore, NRPS-independent siderophores; Other NRPS, hybrid BGCs with at least one NRPS category BGC; NRPS.T1PKS, hybrid BGC with one of NRPS and T1PKS BGCs; Other NRPS.PKS hybrids, hybrid BGC with PKS, NRPS, and other BGCs; RiPP, ribosomally synthesized and post-translationally modified peptide; Other RiPP, hybrid BGCs with one RiPP BGC; All other types, BGCs of other types than above

In this study, we comprehensively analyzed genomes of the *Streptomyces* genus, sourcing both from the public database and from our newly published dataset [22]. As of 30 June 2023, we obtained accession IDs for 2938 *Streptomycetaceae* genomes of all qualities from the NCBI RefSeq database (Additional file 1: Table S1, Fig. 1A). We also incorporated 902 newly sequenced [22] high-quality complete actinomycete genomes for a total of 3840 genomes (Additional file 2: Fig. S1). We note that all of these 902 newly sequenced genomes were assembled de-novo rather than based on a reference genome. These 902 sequences have also been deposited at RefSeq.

These 3840 genomes were then curated further. To ensure a uniform taxonomic classification derived from whole genome sequences, we employed GTDB (version R214) [27, 28] for taxonomic assignments (Additional file 2: Fig. S2). Out of the 3840 genomes, 3569 were identified as belonging to the *Streptomyces* genus. Using different assembly statistics, we grouped the selected 3569 *Streptomyces* genomes into high-quality (HQ, 1215 genomes), medium-quality (MQ, 1156 genomes), and low-quality (LQ, 1198 genomes) (Additional file 2: Fig. S1, S3). The final dataset, post-curation, included 2371 genomes of sufficiently good quality (HQ or MQ).

We next classified the genomes at the species level. Of the 2371 good quality genomes, 1956 were assigned to one of the 608 GTDB-defined species. The species annotation was carried out using GTDB-Tk v2.3 with GTDB database v214 [27, 28]. Four species were highly represented (> 30 genomes) in our dataset: *S. albidoflavus* (or *S. albus*) (109 genomes), *S. anulatus* (58 genomes), *S. olivaceus* (46 genomes), and *S. bacillaris* (33 genomes). There were 415 genomes that did not get assigned to any of the GTDB species, representing potentially novel species beyond the GTDB catalog. To find the total number of species, we used genome similarity across the remaining 415 genomes. We calculated the whole genome similarity using Mash for these 415 genomes. The genomic similarity network was created where the edges represent similarity of greater than 95% (a typical threshold for species detection). We used the community detection method [29] to define the best partitions that were assigned different Mash-based species totaling up to 200 novel species. Combining GTDB and Mash-based assignments, the dataset encompasses at least 808 *Streptomyces* predicted species, with 468 species represented by a single genome. Overall, these statistics indicate that the dataset is highly diverse, necessitating the careful grouping of these genomes for pangenome analysis.

The *Streptomyces* pangenome exhibited wide-ranging genomic characteristics. Genome sizes spanned from 4.8 Mbp to 13.6 Mbp, with a median of 8.5 Mbp (Fig. 1B). Interestingly, the strains with the smallest genome sizes mainly belong to actinomycetoma-related pathogenic species of *S. sudanensis* and *S. somaliensis* [30]. In contrast, the largest-sized genomes primarily belong to *S. rapamycinicus* or other novel species*.* GC content ranged between 68.6 and 74.8%, with a median of 71.6%.

### Types of BGCs identified and similarity to known BGCs

Utilizing antiSMASH v7 [10], we identified a total of 70,561 BGCs in the 2371 HQ or MQ genomes (Additional file 3: Table S4). It is essential to highlight that antiSMASH will report two adjacent unrelated BGCs as a single hit. In general, the genome quality can significantly influence the number of BGCs predicted for a particular genome. Specifically, when BGCs are located on contig edges, their count can be artificially increased when analyzed with antiSMASH as a broken BGC is likely to be counted twice. Thus, the number of BGCs on the contig edge is a metric of genome quality for BGC analysis [31]. We identified only 6524 BGCs (9.2%) situated at contig edges indicating a high quality of the collected dataset at capturing mostly complete BGCs [32]. Among the 1215 genomes with complete assemblies (HQ), the number of BGCs per genome ranged between 11 and 56 with a median of 29 BGCs. To avoid sampling bias in number of genomes per species, we first calculated the means for each of the 808 species individually, which was then averaged to result in the mean of 29.9 BGCs in the *Streptomyces* genus (Additional file 1: Table S2). It should be noted that the set of HQ assemblies included several engineered *S. albidoflavus* strains in which multiple BGCs had been deleted (e.g., [33]), thus explaining the lower BGC count. The number of BGCs increased with the size of the genomes in accordance with prior observations (Fig. 1B) [3, 34]. Previous genome mining studies of *Streptomyces* reported average BGCs in the genus to be 39.64 [3], 44 [19], and 31.8 [18]. The variation in the predicted numbers is likely due to different versions of antiSMASH and the quality of genome assemblies used in the different studies. Plasmids can also be source of BGCs [35]; however, there are limitations on the classification of plasmids, for example circularity and highly diverse plasmid sizes. Of the 1215 HQ genomes, around 352 genomes had BGCs on plasmids with median of 2 BGCs sourced from plasmids among them. For this study, plasmid- and chromosome-encoded BGCs were analyzed together.

The predominant BGC types in our dataset are annotated as: terpene (11,095 BGCs), NRPS-independent (NI) siderophore (5711 BGCs), nonribosomal peptide synthetase (NRPS) (3599 BGCs), type1 polyketide synthase (T1PKS) (3092 BGCs), ribosomally synthesized and post-translationally modified peptide like (RiPP-like) (2933 BGCs), T3PKS (2562 BGCs), ectoine (2458 BGCs), butyrolactone (2277 BGCs), melanin (2244 BGCs), T2PKS (1536 BGCs), and NRPS-T1PKS (1536 BGCs). One can estimate the number of BGCs that are experimentally linked with known secondary metabolites by comparing the BGCs against the curated MIBiG database [16]. This estimate is provided automatically during antiSMASH analysis: the program generates “*knownclusterblast*” similarity scores that estimate how similar a certain region is to the BGCs in MIBiG by calculating a percentage of similar genes [10, 16]. A threshold on the *knownclusterblast* score of greater than 80% of similar genes led to 21,404 BGCs (~ 30%) that matched one of the 475 characterized BGCs from the MIBiG database. The most recurrent known BGCs were linked to the biosynthesis of compounds such as ectoine (2230), desferrioxamine (1685), geosmin (1412), hopene (1095), spore pigment (1083), isorenieratene (852), albaflavenone (807), ε-Poly-L-lysine (730), and alkylresorcinol (708). These BGCs are known to be found commonly across the *Streptomyces* genus [36]. On average, 31% of the BGCs per genome matched to known BGCs in MIBiG. A further 8161 BGCs (~ 11.6%) had similarity scores between 60 to 80%, dominated by 1116 hopene-like BGCs, while as many as 27,029 (38.3%) BGCs had similarity scores of less than 30%. These high numbers also reflect the fact that 41.6% of the MIBiG entries originate from Actinobacteria.

While estimates of novel BGCs provide valuable insights, they inherently depend on the completeness of the MIBiG database, potentially introducing bias. To further dissect this aspect, we examined the number of known BGCs across some of the abundant BGC types (Fig. 1C). We found that certain BGC types—ectoine, NRP-metallophore-NRPS hybrid, T3PKS, T2PKS, lanthipeptide-class-iii, non-alpha polyamino group acids (NAPAA), and terpene—exhibited a significant similarity with the MIBiG database, as evidenced by over 40% of these BGCs having a *knownclusterblast* similarity of above 80%. In contrast, BGC types such as RiPP-like, lanthipeptide-class-i, butyrolactone, NRPS, NRPS-like, T1PKS, and NRPS-like-T1PKS hybrid showed less than 15% of their BGCs aligning with the MIBiG database with the same similarity threshold. However, it is essential to recognize that some BGC types, such as ectoine or NAPAA, are naturally less diverse and represent only a few compounds. For example, the majority of the ectoine-type BGCs (2173 in total) were primarily aligned with just two MIBiG entries, both coding for the same compound ectoine (BGC0000853 and BGC0002052). Similarly, recognized BGC types like NAPAA, lanthipeptide-class-iii, melanin, and NI-siderophore matched fewer than eight MIBiG entries, and some of them are naturally less diverse.

### Mash-based analysis revealed 7 primary and 42 secondary Mash-clusters

Here, we propose a Mash-based whole genome similarity metric to empower comparative pangenome analysis by providing a numerical grouping of strains instead of the taxonomic delineations [23, 25]. Genomic distances were calculated using Mash across all pairs of genomes (Additional file 4: Table S6). The Mash distance is typically correlated with 1—ANI (average nucleotide identity) [25]. The Mash-clusters were generated by optimal K-means clustering in synergy with the highest average silhouette scores (Additional file 2: Fig. S4, Additional file 4: Table S7).

**Fig. 2:**
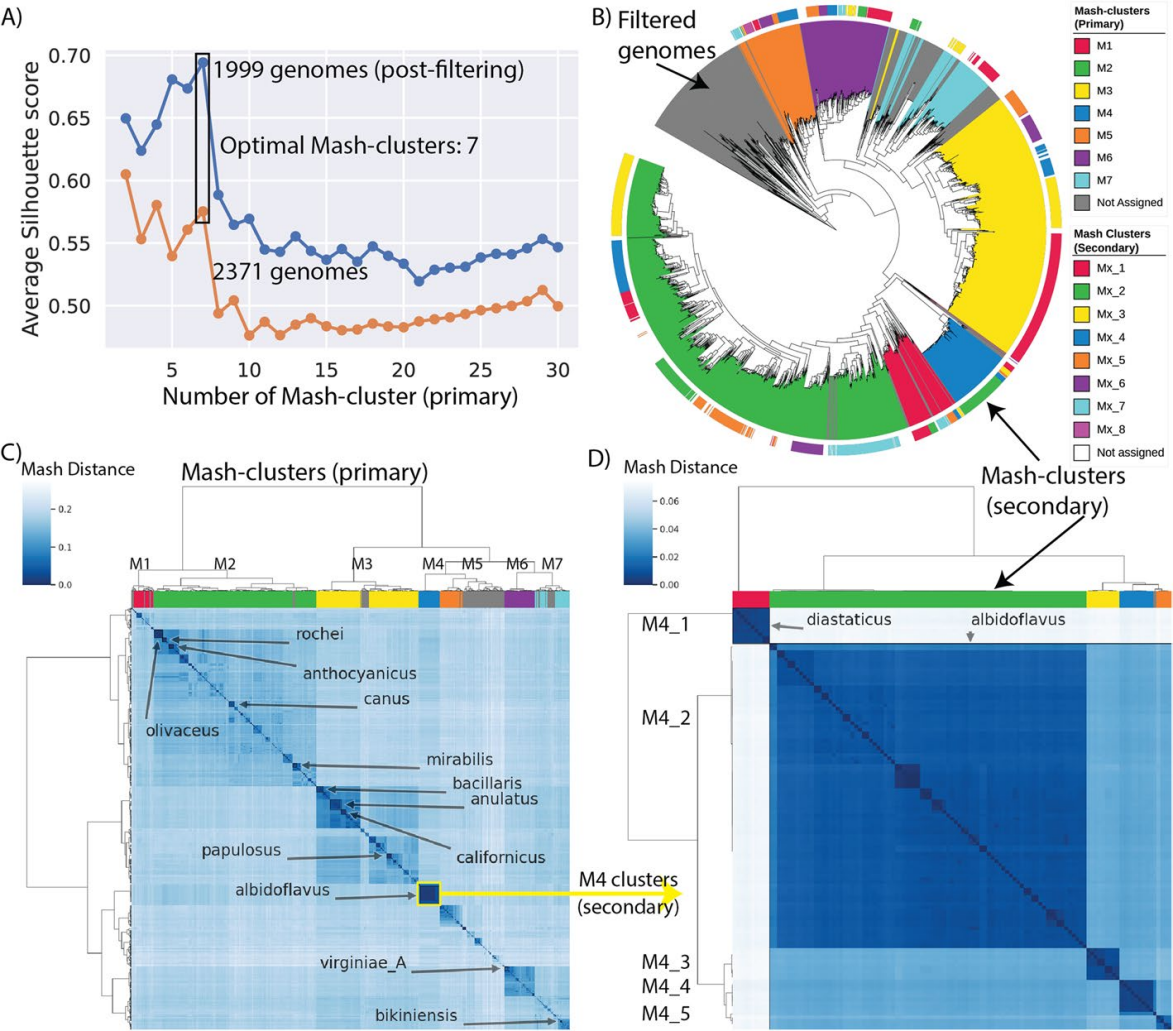
Mash-based clustering of the *Streptomyces* genus provides a basis for pangenome analysis. **A** The average silhouette scores of all samples against the number of primary clusters with hierarchical clustering based on the Mash distance matrix. The orange line represents the original dataset of 2371 genomes, whereas the blue represents the dataset after filtering poorly clustered samples. **B** A phylogenetic tree reconstructed using getphylo with *K. setae* strain KM-6054 as an outgroup. See Additional file 2: Fig. S13 for trees constructed using different methods and the consensus. The colored ranges represent the Mash-cluster assignment with gray color representing filtered genomes. The outer color strip represents the colors for secondary Mash-clusters (see Additional file 2: Fig. S6 to S12 for details). **C** Heatmap representing the Mash distances between the 2371 genomes. The rows and columns are clustered using the hierarchical clustering method where the colors on columns represent the seven primary Mash-clusters (with gray color representing filtered-out genomes). The highlighted text on the heatmap represents some of the abundant species. **D** Heatmap representing the Mash distances between the 119 genomes of the selected M4 cluster. The rows and columns are clustered using the hierarchical clustering method where the colors on columns represent the five secondary Mash-clusters (M4_1 to M4_5). M4_1 represents *S. diastaticus* whereas M4_2 to M4_5 represent different clusters within *S. albidoflavus*

This analysis yielded seven primary Mash-clusters among 1999 genomes, termed M1 through M7 (Fig. 2, Additional file 4: Table S8). To ensure the robustness of these clusters, a stringent silhouette score cutoff (0.4) was iteratively employed, leading to the removal of 372 genomes (Additional file 2: Fig. S5). These filtered genomes are less likely to be part of one of the 7 major Mash-clusters and may form additional clusters upon future sequencing efforts of these clades (Fig. 2B). Venturing deeper, all primary Mash-clusters were subjected to an additional round of clustering, revealing 42 secondary Mash-clusters that encompassed 1670 genomes after refinement based on silhouette scores with the same cutoffs (Additional file 2: Fig. S6-S12). Mash-clusters M5_7, M1_5, and M5_8 were among those with largest number of BGCs per genome with median values being 43, 41.5, and 40, respectively, whereas Mash-clusters M3_5, M2_4, and M1_2 harbored the fewest BGCs on average with median values of 18, 20, and 21, respectively (Additional file 1: Table S3).

Several Mash-clusters stood out in this analysis. M2 emerged as the largest primary Mash-cluster representing 871 genomes. M2 harbors key species such as *S. coelicolor*, *S. rochei,* and *S. canus*. The second largest Mash-cluster, M3, represented 510 genomes with species such as *S. anulatus*, *S. bacillaris* and *S. papulosus*. We note that a significant portion of genomes from Mash-cluster M5 were excluded from the refined Mash-clusters due to their low silhouette scores (Additional file 2: Fig. S5). Mash-cluster M4 represented 119 genomes, mostly of the species *S. albidoflavus* (previously designated *S. albus*), and was noteworthy for its high average clustering score (Fig. 2D, Additional file 2: Fig. S9).

### Comparison of Mash-clusters with phylogenetic trees

The biggest drawback of using a similarity metric like Mash is the lack of an evolutionary model. Therefore, to evaluate the evolutionary relevance of the Mash-clusters, we compared them to genome-scale phylogenetic trees (Fig. 2B). We constructed three trees by employing three distinct methodologies: autoMLST [37], GTDB-Tk (de novo workflow) [28], and getphylo [38] (Additional file 2: Fig. S13, Additional file 5). Upon comparison, a broad consensus was observed between the Mash-defined clusters and the clades delineated by different phylogenetic trees. There were, however, some outliers, chiefly clusters M1 and M7, which appeared to be paraphyletic (Additional file 2: Fig. S13). Upon closer inspection, however, these outliers fell within parts of the phylogenetic trees that were poorly supported and incongruent between the different methodologies. Further analysis revealed a striking level of incongruence between the three phylogenies. Only 62% of the branches were supported by a majority consensus and 33% by all three methodologies. The genus *Streptomyces* and its two major clades (represented by M2 and M3) are fully congruent as well as many of the species and species complexes. However, lineages show a high degree of polytomy at the sub-generic level. This incongruence demonstrates the potential fallibility of phylogenetic methods when studying the intra-genus level relationships of *Streptomyces*.

Finally, we also compared the Mash-clusters with the RED groups (relative evolutionary divergence-based groups) defined in a recent study as bacterial groups analogous to genera but characterized by equal evolutionary distance [8] (Additional file 2: Fig. S14). A consensus was observed for major groups except that the Mash-based method has split the RG_2 into two separate Mash-clusters, M3 and M6. In this fashion, Mash-clustering proposed here complements the phylogenetic methods to produce statistically correlated groups.

### BGC diversity predictions based on known cluster similarity

**Fig. 3:**
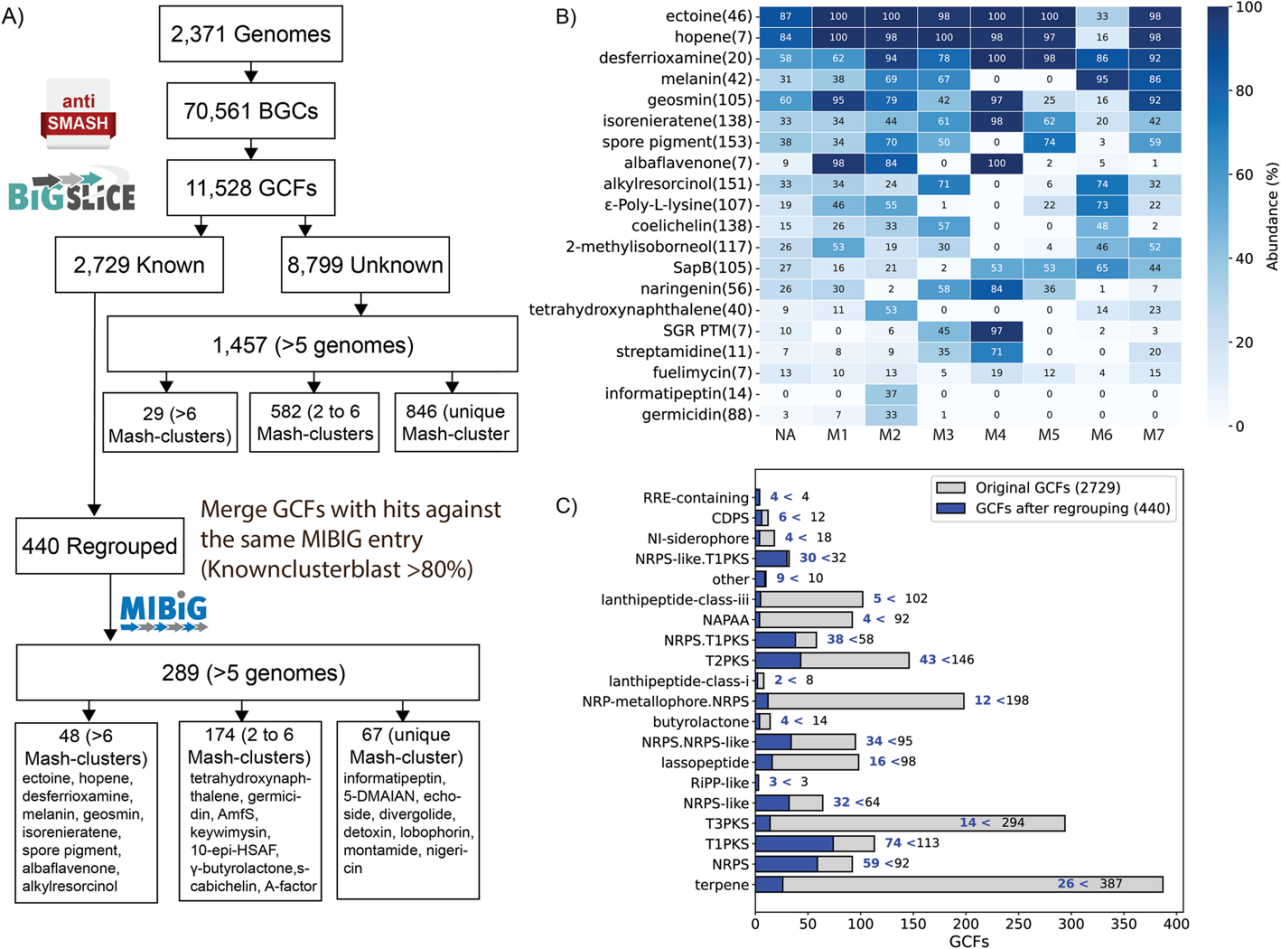
Advanced clustering of BGCs redefines known GCFs with reduced diversity in specific types of BGCs. **A** Workflow used to detect BGCs, GCFs based on BiG-SLICE, and regrouping GCFs based on *knownclusterblast* similarity (> 80% of genes). Several examples of known GCFs are reported in the bottom boxes, classified into common, accessory, or unique GCFs to Mash-clusters. **B** Percentage abundance of the top twenty known GCFs across different primary Mash-clusters. Each row corresponds to a known compound (GCF). The number in parentheses denotes the number of BiG-SLICE detected GCFs that were regrouped into one GCF. **C** Overview of the number of GCFs that were regrouped across the twenty most abundant BGC types. Gray bars represent the number of GCFs detected using only BiG-SLICE, whereas blue bars represent the reduced number of GCFs after regrouping based on *knownclusterblast*

To assess the diverse biosynthetic potential across genomes, it is helpful to group BGCs into gene cluster families (GCFs). GCFs are groups of BGCs that are homologous to each other and thus are hypothesized to encode molecules that have similar chemical structures. GCFs are calculated by clustering BGCs using specialized tools such as BiG-SCAPE [11] or BiG-SLICE [12]. As a first step, we opted for BiG-SLICE (optimal with larger datasets) to execute this clustering across the entire dataset. Utilizing default parameters, we identified a total of 11,528 GCFs from the 70,561 BGCs (Fig. 3A, Additional file 6: Table S9). However, as highlighted in previous work [17], integrating diverse genome mining tools can enhance GCF refinement. For instance, minor genetic variations in regions adjacent to, but not directly involved in, biosynthesis can inadvertently lead to the classification of BGCs that code for identical secondary metabolites into disparate GCFs. To mitigate these issues, as a second step of defining GCFs, we used antiSMASH’s *knownclusterblast* results (with a similarity threshold of > 80% of genes) to regroup the GCFs with the presence of predicted known BGCs.

A total of 2729 GCFs predicted by BiG-SLICE in the first step were associated with known secondary metabolites according to *knownclusterblast* results. After regrouping these GCFs at the second step, we effectively reduced the count of known GCFs from 2729 to 440 (Fig. 3A, Additional file 6: Table S10-S11). For instance, BGCs that code for spore pigment, alkylresorcinol, coelichelin, and isorenieratene (in the second step) were detected as 153, 151, 138, and 138 different GCFs (in the first step), respectively (Fig. 3B). We investigated whether these reductions of GCF diversity predictions are dependent on the type of BGCs (Fig. 3C). For example, BGC types such as lanthipeptide-class-iii, terpene, NRP_metallophore-NRPS hybrid, and T3PKS showed a high level of reduction in the diversity of GCFs when *knownclusterblast* results were integrated. In contrast, types such as NRPS or T1PKS showed a relatively lower reduction in the diversity of GCFs as was predicted in the first step using BiG-SLICE (Fig. 3C).

We also note that these regrouped GCFs could contain minor internal variations. For a more precise investigation, we constructed a similarity network of two regrouped GCFs coding for spore pigment (153 originally predicted GCFs) and isorenieratene (138 originally predicted GCFs). We used a BiG-SCAPE generated distance matrix to create this network (more optimal for a relatively small dataset) (Additional file 2: Fig. S15A, and S15C). We also aligned the selected BGCs, which showed that the overestimated diversity of these known BGCs can be attributed to inaccurate BGC boundaries. For instance, variation in BGCs from different Mash-clusters was largely due to differences in the neighboring regions of the detected BGCs. The differential neighboring regions causing variation within the regrouped GCF were generally conserved within genomes from the same Mash-clusters (Additional file 2: Fig. S15B and S15D). In general, we observed that the types requiring fewer genes for core biosynthesis, such as terpene, T2PKS, T3PKS, siderophore, or RiPPs, were also among the most affected by these variations in neighboring regions.

The regrouping of 2729 known GCFs was carried out on the basis of the similarity to nearest MIBiG BGCs. For the remaining 8799 unknown GCFs, this analysis was not possible at this stage. This diversity of unknown GCFs is also likely an overestimation which cannot yet be tested by our approach.

### Diversity of GCFs across genomes from different Mash-clusters

Subsequently, we examined the distribution patterns of GCFs across the genomes delineated by the seven primary Mash-clusters to identify BGCs associated with specific Mash-clusters (Fig. 3B). Mash-cluster M2 contained 2606 GCFs that did not appear in any other Mash-cluster. Similarly, Mash-clusters M3 and M6 contained 811 and 648 GCFs, respectively, that were specific to those Mash-clusters (Additional file 2: Fig. S16). We also note that a total of 2338 GCFs were specific to the 372 genomes that were dropped from the Mash-cluster definitions and are likely to represent further diversity. It is imperative to note that Mash-clusters M2 and M3 constitute the most populous clades which may explain their apparent diversity of GCFs.

To gain deeper insights into the biosynthetic signatures of different Mash-clusters, we analyzed all GCFs containing at least five BGCs. This encompassed 289 GCFs with experimentally linked compounds from the MIBiG database and 1457 putatively novel GCFs. We found that 48 of the “known” GCFs (such as ectoine, hopene, and desferrioxamine) displayed a widespread genomic distribution, being present in genomes across all Mash-clusters. We detected 174 known GCFs (such as germicidin, streptamidine, and SGR-PTM) in the genomes across multiple, but not all, Mash-clusters. Finally, 67 of the “known” GCFs (such as informatipeptin, 5-DMAIAN, and echoside) were specific to genomes from only one of the major Mash-clusters, representing the biosynthetic signatures of these groups of genomes (Additional file 2: Fig. S17). We also observed the same pattern of conservation of unknown GCFs in specific Mash-clusters (Additional file 2: Fig. S18). This observed presence of GCFs across Mash-clusters implies certain BGCs are likely to be found in certain Mash-clusters at primary or secondary levels (Additional file 2: Fig. S17-S18).

### Conservation of chromosomal synteny of BGCs

Finally, we present a novel workflow to capture BGC diversity by analyzing synteny within a Mash-cluster. The diversity of the BGCs and their functions is computationally predicted using similarity metrics and by visualization of the similarity networks (e.g., using BiG-SCAPE detected similarity scores). To explore the syntenic relationship between BGCs, we extended this network by adding edges between the BGCs that are neighbors on the chromosomes. Thus, all BGCs in any genome would be connected by edges in the order of presence on chromosomes.

**Fig. 4:**
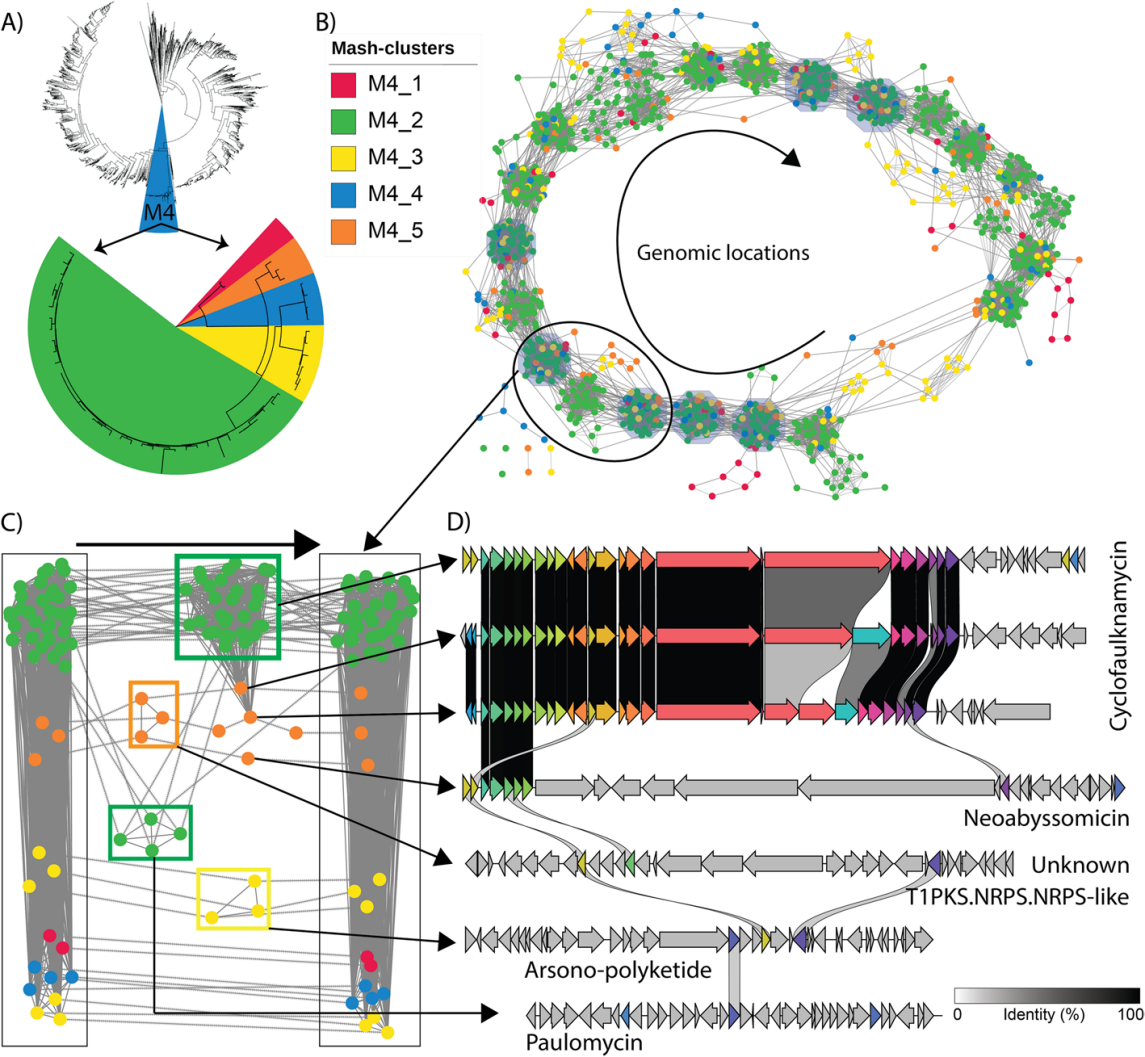
Synteny of BGCs across Mash-clusters M4_1 to M4_5 showed conserved and variable regions. **A** Phylogenetic tree (top) of all 2371 genomes with highlighted M4 primary Mash-cluster. Phylogenetic tree (bottom) of complete HQ genomes from the M4 primary Mash-cluster grouped into five secondary Mash-clusters M4_1 through M4_5. M4_1 represents *S. diastaticus* whereas M4_2 to M4_5 represent different clusters within *S. albidoflavus*. **B** Synteny network view of GCFs where the nodes represent detected BGCs across 49 high-quality complete genomes from M4. Seven of the BGCs were present across all 49 genomes and in the same order. The edges with solid lines represent BiG-SCAPE-based similarity between BGCs. **C** A selected portion of the synteny network from part B. The leftmost BGC is a type 2 lanthipeptide and the rightmost BGC is a NI-siderophore. They are two of the seven BGCs conserved in all genomes. The middle BGCs are variable. **D** Alignment of several variable BGCs from part C across strains from different secondary Mash-clusters

As an example, we selected 49 complete genomes from Mash-cluster M4 that were further grouped into five secondary Mash-clusters (M4_1 to M4_5) (Fig. 4A, Additional file 7). These strains primarily belonged to *S. albidoflavus* (M4_2 to M4_5) and *S. diastaticus* species. The resulting network of BGCs showed remarkable conservation of the order in which the BGCs have evolved on the chromosomal location (Fig. 4B). We observed that different BGCs are either inserted or deleted from specific locations while maintaining the order of the seven commonly present BGCs across the M4 Mash-cluster genomes. We also observed that these differences are conserved within the secondary Mash-clusters (Fig. 4B). This observation implicates the vertical inheritance of BGCs as strains evolve across different clades or groups.

We focused on a specific region between two of the conserved BGCs coding for a type 2 lanthipeptide and NI-siderophore (Fig. 4C). The genomes belonging to Mash-clusters M4_1 and M4_4 did not possess any BGCs in this chromosomal region, along with some of the M4_3 genomes. The majority of the M4_2 genomes harbored an NRPS BGC coding for the known molecule cyclofaulknamycin (Fig. 4D). The genomes of the M4_5 Mash-cluster showed interesting variation in this region. Two genomes were observed to harbor a reduced version of the cyclofaulknamycin BGC that could have a differential or loss of function, whereas the other M4_5 genome has acquired a completely different T1PKS BGC in the same region, one that codes for neoabyssomicin (Fig. 4D) [39]. The genomes in M4_5 also harbor a yet uncharacterized T1PKS-NRPS hybrid BGC in the region. Some of the M4_2 genomes have additional BGCs in the region coding for the known PKS-like molecule paulomycin, whereas the others from M4_3 have a T1PKS-PKS-like hybrid BGC that codes for arsono-polyketide, which is widespread across *Streptomyces sp.* [40].

**Fig. 5:**
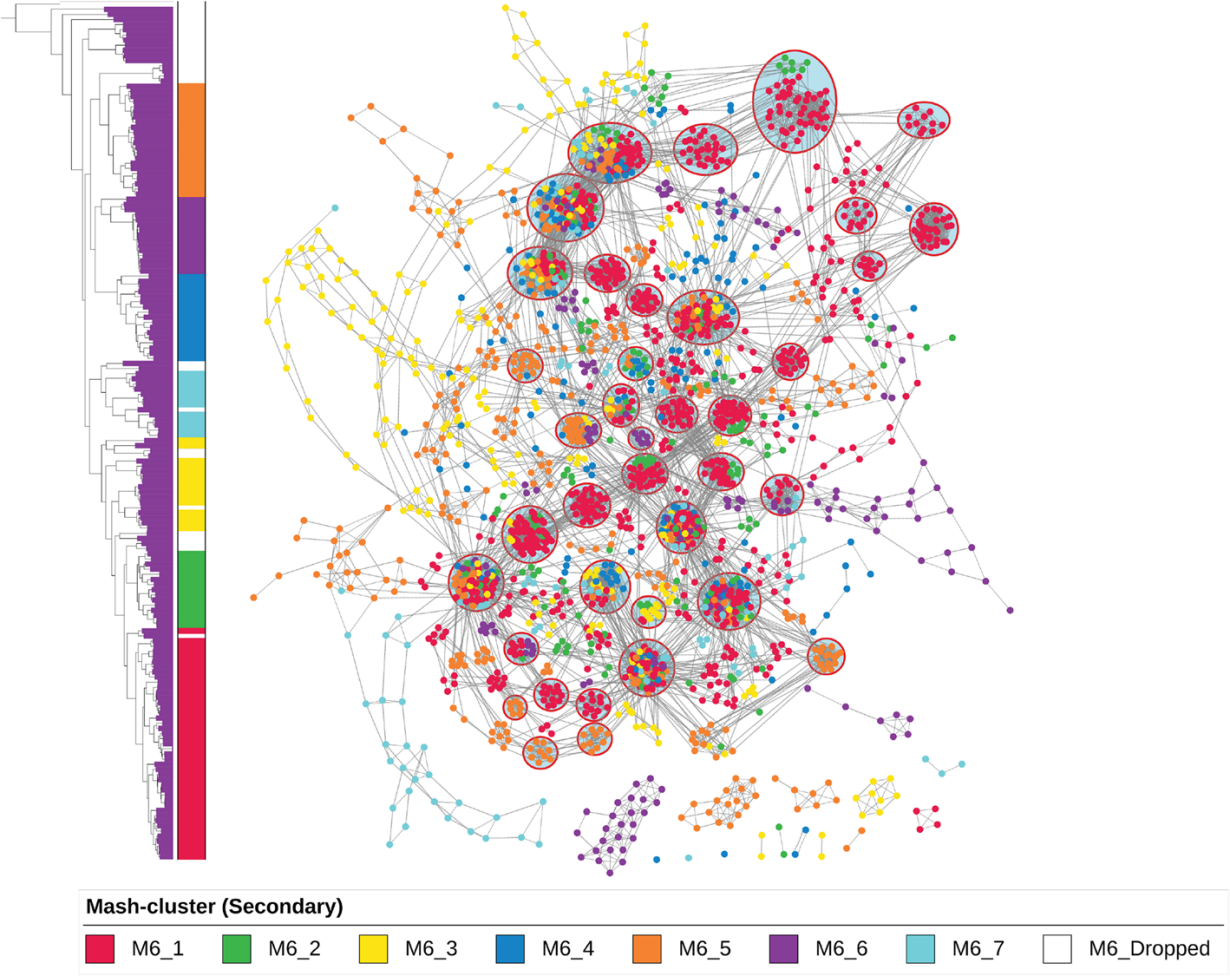
Synteny of BGCs across diverse Mash-clusters M6_1 to M6_7 showed high-level conservation of chromosomal order. Different colors of nodes represent secondary Mash-clusters from M6_1 to M6_7 which are also shown on the phylogenetic tree by the color bar. The top 40 most common GCFs across and specific to Mash-clusters are annotated by open red circles that enclose the GCFs (nodes)

In another example, we selected genomes from a more diverse Mash-cluster, M6, that consists of as many as 63 species, compared to the M4 Mash-cluster with only two species. The similarity network of BGCs displayed high diversity across genomes of the M6 Mash-cluster (Fig. 5, Additional file 2: Fig. S19 to S26); however, a conserved structure could still be observed. Common BGCs forming the most abundant GCFs were found to generally follow a chromosomal order with variations appearing between different secondary Mash-clusters. Conservation is clearer when comparing subclusters as genomes belonging to the same secondary Mash-cluster share a higher number of common BGCs. For example, a similarity network of genomes from 8 different species from Mash-cluster M6_1 reveals the conserved and variable regions on the chromosomes across species (Additional file 2: Fig. S20). Higher silhouette scores indicated greater conservation of BGCs. M6_1 and M6_2 that had higher silhouette scores displayed high conservation of the BGCs as well as the chromosomal order of BGCs (Additional file 2: Fig. S19). Conversely, M6_3 and M6_7 had the most divergent BGCs in comparison to other Mash-clusters (Fig. 5, Additional file 8). These two secondary Mash-clusters had lower silhouette scores and thus represent poorly clustered groups (Additional file 2: Fig. S11). As with the above example, dot plots can be used to visualize this relationship in a pairwise fashion (Additional file 2: Fig. S27-S28)—notably, the terminal regions of the genome are much less conserved than in M4.

We observed a similar pattern of conservation of the chromosomal order of common BGCs within all secondary Mash-clusters of M6, with the pattern being the most evident at species level (Additional file 2: Fig. S21-S28). In addition, we also found a similar pattern among the five species within the M2_3 secondary Mash-cluster, which includes the model strain *S. coelicolor* A3(2) (Additional file 2: Fig. S29). Thus, the strategy to integrate BGC similarity and synteny provides an effective way to select different levels of Mash-clusters and investigate them individually.

## Discussion

In this study, we conducted pangenome mining of the biosynthetic potential inherent in the *Streptomyces* genus, leveraging a dataset totaling over 2370 genomes. Our investigative approach was underpinned by a comprehensive workflow that encompassed crucial steps for robust analysis. These steps included taxonomic identification, data quality checks, and Mash-based clustering as well as the detection of BGCs and GCFs. Furthermore, our methodology involved the regrouping of known GCFs to discern functional diversity and a thorough examination of synteny among BGCs distributed across the chromosomes. This comprehensive analytical framework has provided insights into both genomic architecture and the functional diversity inherent in these prolific secondary metabolite producers.

We emphasized the critical role of data curation as the foundational step in comparative genomic analysis, ensuring the establishment of a consistent dataset. Our workflow included an assessment of critical assembly metrics such as the number of contigs, N50 score, completeness, and contamination, enabling the classification of genomes into high, medium, and low quality. Within the vast genomic landscape of the *Streptomyces* genus, the clearly defined classification of strains is essential for comparative analysis. Historically, comparative genome mining studies have predominantly centered on examining single species. However, this approach is limiting, especially in a genus like *Streptomyces* where many species are represented by a single genome sequence in public databases. This limitation has often necessitated a broader lens, encompassing genomes from the entire genus [8, 18, 19]. As valuable as genus-level insights are, the detection of over 800 species of *Streptomyces* demands a more focused approach. Accordingly, we sought to define distinct, sub-genus level groups based on clustering Mash-based similarities. This methodology not only facilitated the grouping of genomes into distinct clusters but also prioritized those that were consistently clustered. While acknowledging the inherent limitations of clustering algorithms, we employed a strict silhouette score as a necessary metric, recognizing that these algorithms have their drawbacks, especially when dealing with unevenly distributed starting datasets. Consequently, we omitted 372 strains from Mash-cluster assignments, prioritizing the integrity of our analytical framework. Validation of the Mash-cluster definitions against different phylogenetic trees underscored the robustness of this grouping strategy for comparative analysis.

The description of bacterial species requires the adoption of strict criteria on the naming, genotyping and phenotyping of the organism of interest [41]. Bacterial taxonomy is extremely valuable to our understanding of diversity but is time consuming and requires specific expertise and resources not available to many labs. Given the vast rate at which new genomes are acquired (now on the order of 1000 s [22]), it is not possible to gain complete taxonomic classification for entire collections expediently. Mash-clustering does not obviate the need for proper taxonomic analysis; however, it provides an objective measure to create useful groupings for comparative analysis. We found the diversity and classification of GCFs to be notably influenced by several factors, including the type of BGC, the definition of BGC boundaries, and the completeness of the MIBiG database. This observation emphasizes the crucial role of manual inspection and refinement of existing genome mining tools in accurately characterizing the inherent diversity of detected BGCs. In the course of our study, the integration of similarity scores derived from *knownclusterblast* with the BiG-SLICE-based network highlighted a noteworthy finding—that the diversity of computationally predicted BGCs may be considerably lower, especially in BGC types where the core biosynthetic regions are notably smaller than the predicted boundary regions. While we anticipate that improvements in GCF detection algorithms may yield more accurate predictions, the prediction of boundaries remains a substantial challenge in the genome mining field.

Leveraging the definition of Mash-clusters in our analysis, we identified GCFs demonstrating specificity or commonality across distinct Mash-clusters. Some of the common GCFs putatively coded for secondary metabolites such as ectoine, hopene, and desferrioxamine among others. This approach also facilitated the discernment of signature BGCs associated with groups of strains at different Mash-cluster levels. It is crucial to note that the variable size of Mash-clusters introduced variability in the number of signature BGCs observed across different clusters. As genome mining advances, these insights contribute to the ongoing refinement of methodologies, paving the way for more accurate and comprehensive assessments of biosynthetic potential across microbial genomes.

A detailed exploration of BGCs within Mash-cluster M4, which was further categorized into five secondary Mash-clusters, uncovered a striking observation—BGC order along the chromosome appears to be conserved. We observed shared genomic events such as deletions, insertions, and modifications of BGCs in specific chromosomal regions across distinct secondary Mash-clusters. Importantly, these patterns extend beyond M4, resonating across various Mash-clusters and species. Our investigation extends to the various secondary Mash-clusters M6_1 to M6_7. We observed that the pattern of conserved chromosomal organization of common BGCs becomes clearer as one goes from primary to secondary Mash-clusters and is most prominent at the species level. Generally, the Mash-clusters with higher average silhouette scores presented a pattern of the conserved organization of BGCs more evidently. Furthermore, this pattern is exemplified by a comparative analysis involving five species, including *S. coelicolor*, within the secondary Mash-cluster M2_3 (Additional file 2: Fig. S29). Over and over again, the similarity networks of closely related genomes from species or Mash-clusters showed conserved order of common BGCs and variable BGCs in the neighborhoods between conserved BGCs. Such representation could help to show how neighboring species, or closely related genomes, have evolved to harbor diverse BGCs at specific chromosomal positions.

There has been significant debate over the role of horizontal gene transfer (HGT) versus vertical inheritance in the evolution of BGCs [42, 43]. Previous genomic analyses suggested that HGT plays an important role in the evolution of BGCs [43–46]. However, evidence is building that horizontal gene transfer is much rarer than previously thought in *Streptomyces* spp. [42]. Conversely, there is mounting evidence that processes associated with vertical inheritance such as gene duplications and intragenomic recombination play an important role in the diversification of BGCs [47]. The observation that BGCs belonging to related GCFs share conserved synteny across the Mash-clusters adds additional evidence to the argument that vertical inheritance is more important than previously thought. Our findings underscore the role of vertical descent in the evolution of BGCs across species and Mash-clusters, aligning with a growing body of evidence in the literature [14, 48, 49].

With the exponential growth of genome sequencing, the influence of vertical descent is becoming increasingly apparent in the evolution of BGCs. The findings from this study significantly contribute to our understanding of these vertical inheritance mechanisms along with a need for manual inspection to more accurately capture the functional diversity of GCFs. These insights have broader implications for understanding the adaptive strategies employed by these prolific secondary metabolite producers in diverse ecological niches and environments.

## Conclusions

In conclusion, our study presents a pangenome analysis of the biosynthetic diversity of *Streptomyces*, a genus of high industrial importance. Data-driven clustering of nearly 2400 *Streptomyces* genomes into Mash-clusters revealed (1) the diversity (or lack thereof) of computationally predicted BGCs, especially when automatically grouped into GCFs, (2) that certain BGCs/GCFs are specific to certain Mash-clusters, thus acting as potential biosynthetic signatures for the Mash-cluster, and (3) that synteny among BGCs are conserved, implying that vertical inheritance plays a major role in the evolution of BGCs. Our results on GCF conservation at taxonomic levels support that the BGCs are key traits of species defining their ecotypes. Taken together, our work not only contributes to advancing our understanding of secondary metabolite biosynthesis in *Streptomyces* but also highlights the evolving capabilities of pangenome analytics for biosynthetic diversity exploration.

## Methods

### Data collection, taxonomy detection, and quality check

The starting dataset to select *Streptomyces* genomes was gathered from two sources: NCBI and from those presented in a recent study [22]. As of 30 June 2023, we collected a total of 2938 genomes of all assembly levels from NCBI RefSeq belonging to the family *Streptomycetaceae* (Additional file 1: Table S1). We used this broader family of *Streptomycetaceae* with the aim of assigning taxonomy based on GTDB consistently (version R214) [27, 28]. We collected an additional 902 of the 1034 actinomycete genomes from a recent study [22] (Additional file 1: Table S1). We note that 121 genomes of the 1034 were already available on NCBI on 30 June 2023, and 11 were added later to the other study [22]. These genomes were processed through BGCFlow, and different tools to assess the quality of the genomes were run [17]. Out of these 3840 genomes, 3569 were identified as belonging to the *Streptomyces* genus as per GTDB definitions (Additional file 1: Table S1, Additional file 2: Fig. S2).

The *Streptomyces* dataset of 3569 genomes was processed with multiple quality checks. We calculated genome completeness and contamination metrics using CheckM [50]. When cutoffs of greater than 90% completeness and less than 5% contamination were used, 59 genomes were found to have low-quality assemblies (Additional file 2: Fig. S3). We also used the assembly statistics on the contigs and N50 scores for further curation. The genomes designated as complete or chromosome-level assembly as per NCBI were classified as high-quality (HQ). From the remaining genomes with scaffold or contig level assembly, we further annotated the genomes with more than 100 contigs or N50 score of less than 100 kb as low-quality (LQ). Genomes with fewer than 100 contigs were classified as medium-quality (MQ) (Additional file 2: Fig. S3).

For the 415 genomes that lacked species assignments in GTDB-Tk analysis, we calculated the Mash-based similarity network where the edges represent genome-wide similarity of greater than 95% (typical threshold for species detection). We used the community detection method [29] to define the best partitions that were assigned different Mash-based species.

### Mash-based clustering analysis to group Streptomyces species

We used a whole genome sequence similarity-based workflow to cluster the genomes into different subgroups of the *Streptomyces* genus. A similar workflow with Mash-based analysis was shown to capture the phylogroups previously [23]. Following this method, we calculated Mash-distance for all pairs of genomes in the dataset using a BGCFlow rule that runs Mash with K-mer size of 21 (Additional file 4: Table S4) [25]. We computed pairwise distances using Pearson’s correlation coefficient and performed hierarchical clustering using the ward.D2 method.

We added additional steps to the Mash-based analysis method [23] to identify the optimal number of clusters. We followed the elbow method to find the optimal number of k-means clusters and validated them using the average silhouette scores. We detected 7 optimal clusters based on both adjusted inertia for the K-means method and the high average silhouette score across the given dataset (Additional file 2: Fig. S4). The heatmap visualizations represented the diverse Mash-clusters defined here (Additional file 2: Fig. S4). Next, we visualized the silhouette scores across different Mash-clusters to validate the clustering using swarm plots (Additional file 2: Fig. S5). A random cutoff of 0.4 was chosen to select the genomes that have good cluster assignments. This cutoff results in the majority of the dataset being clustered consistently (except for Mash-cluster M5 that appears to be poorly clustered). We iteratively removed the poorly clustered genomes from the dataset until all genomes consistently scored above 0.4 on silhouette scores.

These curated steps resulted in the assignment of 1999 genomes to a valid Mash-cluster (Additional file 4: Table S8). We further identified Mash-clusters within each of the above-defined primary Mash-clusters. This secondary level of analysis led to the identification of 42 consistent secondary Mash-clusters across 1670 of the 1999 genomes. We note that the assignment of the Mash-clusters is dependent on the abundance of genomes collected in each cluster and will likely change as the number of genomes increases.

### Comparing Mash-clusters against phylogenetic trees

Phylogenetic trees were inferred from all 2371 curated *Streptomyces* genomes using an outgroup genome of the *Kitasatospora* genus (*K. setae* strain KM-6054). We used 3 different methods: GTDB-Tk [28], autoMLST [37], and getphylo [38]. The consensus tree was calculated using IQ-Tree2 [51]. RED groups (relative evolutionary distance) were calculated in a prior phylogenetic study based on GTDB [8]. The tree visualizations were generated using iTOL [52].

### Genome mining to detect BGCs

All genomes were annotated or reannotated using prokka v1.14.6 [53]. We also used a list of seven selected genomes with high-quality manually curated annotations as a priority while running prokka using the parameter “*–proteins*”. We used antiSMASH v7.0.0 on the annotated genomes to detect secondary metabolite BGCs (Additional file 3: Table S4) [10]. The *knownclusterblast* results were used for primary assessment of whether the detected BGC regions show substantial similarity against the BGCs from MIBiG database [54]. We used a strict cutoff of greater than 80% *knownclusterblast* similarity to tentatively identify BGCs that produce known secondary metabolites (Fig. 1C, Additional file 3: Table S5). BGCs with 50 to 80% similarity were similarly marked as producers of known secondary metabolites but with lower confidence.

### Detection of GCFs

We used BiG-SLICE to calculate gene cluster families (GCFs) from identified BGCs using the default parameters (threshold of 900) [12] (Additional file 6: Table S9). We further annotated the GCFs as known if they had BGCs with *knownclusterblast* similarity above 80%. Different GCFs that contained BGCs with hits against the same MIBiG entry were combined into a single “regrouped” GCF and putatively associated with known BGCs (Additional file 6: Table S10-S11). The abundance of some of the common GCFs (after regrouping) was calculated across different Mash-clusters (Fig. 3B). The UpSet plot was used to visualize the overlap of GCFs across Mash-clusters (Additional file 2: Fig. S16). Selected BGCs from two GCFs putatively coding for spore pigment and isorenieratene were further extracted for in-depth comparison. For more accurate similarity calculation, we used BiG-SCAPE to generate a similarity network with a default threshold of 0.3 on the distance metric [11]. The network was visualized using Cytoscape where node colors represented different Mash-clusters [55]. Representative BGCs from different BiG-SCAPE predicted GCFs were further chosen to visualize the BGC region alignment using clinker tool [56] (Additional file 2: Fig. S15).

### Integrated network of BGCs similarity and chromosomal order

We developed a custom workflow to simultaneously visualize BGC diversity and the order of BGCs along the chromosome. As a case study, we selected BGCs from 49 high-quality complete genomes from Mash-cluster M4 that spanned 5 secondary-level Mash-clusters (Additional file 7). Each node in the network represents a BGC, and nodes were connected with two types of edges. The first type represented BiG-SCAPE-based similarity. The second type reflected the order of BGCs present on the chromosome. A specific region of the chromosome with two conserved BGCs was extracted for manual inspection of the variation of this region. The selected BGCs were visualized using the clinker [56] to observe the alignments. A similar integrated network was also reconstructed for 23 genomes from 5 different GTDB-defined species that belonged to Mash-cluster M2_3. Dot plots were generated using a custom Python package “dotplotter” v 1.0.0 that was created for this study to plot the results of a pairwise blastn searches (https://github.com/drboothtj/dotplotter [57]).

## Supplementary Information

Additional file 1. Metadata tables of the genomes used. Table S1: Genomes Metadata. List of 3840 genomes used at the start of the study with information on the source, quality assigned, taxonomic information based on GTDB, checkM metrics of assembly quality, bioproject accession numbers for all genomes, assigned Mash-clusters at two levels for selected Streptomyces genomes, and the RED groups from prior study. Table S2. Average BGCs (species). Number of BGCs on average across 808 detected species. Table S3. Average BGCs (Mash-clusters). Number of BGCs on average across 42 secondary Mash-clusters.

Additional file 2. Supplementary figures S1 to S29. Includes supplementary figures and the legends for Fig. S1 to S29.

Additional file 3. Tables for detected BGCs across 2371 genomes of HQ and MQ quality. Table S4: BGCs count. Table with number of BGCs detected in each of the genomes analyzed. Table S5: BGC information. Metadata table with information on each of the detected BGCs.

Additional file 4. Tables for Mash-based clustering and silhouette scores. Table S6. Mash distances. Table with Mash based distances across all 2371 selected genomes. Table S7. Silhouette scores (primary). Table with list of 2371 genomes with assigned clusters based on clustering analysis. The columns represent the assigned clusters after each filtering round (upto 5). The genomes being removed based on silhouette score cutoff of 0.4 are annotated as “Dropped”. The columns also mention the silhouette score at each round of filtering. The final round includes 1999 genomes with primary Mash-cluster assignments. Table S8. Silhouette scores (secondary). Table with list of 1999 genomes with assigned clusters at secondary level based on clustering analysis. The columns represent the assigned clusters after each filtering round (upto 3). The genomes being removed based on silhouette score cutoff of 0.4 are annotated as “Dropped”. The columns also mention the silhouette score at each round of filtering. The final round includes 1670 genomes with secondary Mash-cluster assignments.

Additional file 5. Phylogenetic trees using different methods. Three different phylogenetic tree files were calculated using autoMLST, getphylo and GTDB-Tk methods. The iTOL project with all the phylogenetic tree can be found at the link below: https://itol.embl.de/shared/omkar31.

Additional file 6. Tables for detected GCFs using BiGSLiCE and regrouping of known GCFs. Table S9. GCFs (BiGSLICE). List of detected GCFs using BiGSLICE with metadata on number of BGCs and combined GCF ID that were regrouped based on shared known clusters blast hits. Table S10. GCFs (Regrouped): List of GCFs as defined in this study using BiGSLICE along with knownclusterblast similarity with metadata on number of BGCs BiGSLICE defined GCFs. Table S11. BGC information: Assignment of GCFs and combined GCFs for each BGC.

Additional file 7. Cytoscape file of BiG-SCAPE similarity network for BGCs in M4 Mash-cluster. The network visualizations corresponding to Figs. 4B and 4C. The network included edges based on BiGSCAPE similarity. Additional edges were added if the BGCs appeared next to each other on the chromosome.

Additional file 8. Cytoscape file of BiG-SCAPE similarity network for BGCs in M6 Mash-cluster. The network visualizations corresponding to Fig. 5. The network included edges based on BiGSCAPE similarity. Additional edges were added if the BGCs appeared next to each other on the chromosome.

Additional file 9. Review history. The review history for this manuscript.

## Data Availability

All the data is available as supplementary materials in Additional files 1 to 8. Additional file 1: Table S1 contains the accessions of all the genomes used in the study. The general workflow used to generate the data is available at https://github.com/NBChub/bgcflow [58]. The specific downstream analysis for this manuscript is available at GitHub link https://github.com/NBChub/Streptomyces_pangenome_ms [59] and version of the code is also deposited at Zenodo https://doi.org/10.5281/zenodo.14310728 [60].

## References

[1] Scherlach K, Hertweck C. Mining and unearthing hidden biosynthetic potential. Nat Commun. 2021;12:3864. 34162873 10.1038/s41467-021-24133-5 PMC8222398

[2] Lee N, Hwang S, Kim J, Cho S, Palsson B, Cho B-K. Mini review: Genome mining approaches for the identification of secondary metabolite biosynthetic gene clusters in Streptomyces. Comput Struct Biotechnol J. 2020;18:1548–56. 32637051 10.1016/j.csbj.2020.06.024 PMC7327026

[3] Belknap KC, Park CJ, Barth BM, Andam CP. Genome mining of biosynthetic and chemotherapeutic gene clusters in Streptomyces bacteria. Sci Rep. 2020;10:2003. 32029878 10.1038/s41598-020-58904-9 PMC7005152

[4] Omura S, Ikeda H, Ishikawa J, Hanamoto A, Takahashi C, Shinose M, et al. Genome sequence of an industrial microorganism Streptomyces avermitilis: deducing the ability of producing secondary metabolites. Proc Natl Acad Sci U S A. 2001;98:12215–20. 11572948 10.1073/pnas.211433198 PMC59794

[5] Bentley SD, Chater KF, Cerdeño-Tárraga A-M, Challis GL, Thomson NR, James KD, et al. Complete genome sequence of the model actinomycete Streptomyces coelicolor A3(2). Nature. 2002;417:141–7. 12000953 10.1038/417141a

[6] Nikolaidis M, Hesketh A, Frangou N, Mossialos D, Van de Peer Y, Oliver SG, et al. A panoramic view of the genomic landscape of the genus Streptomyces. Microb Genom. 2023;9. Available from: 10.1099/mgen.0.001028 10.1099/mgen.0.001028 PMC10327506 37266990

[7] Komaki H. Recent progress of reclassification of the genus Streptomyces. Microorganisms. 2023;11. Available from: 10.3390/microorganisms11040831 10.3390/microorganisms11040831 PMC10145440 37110257

[8] Gavriilidou A, Kautsar SA, Zaburannyi N, Krug D, Müller R, Medema MH, et al. Compendium of specialized metabolite biosynthetic diversity encoded in bacterial genomes. Nat Microbiol. 2022;7:726–35. 35505244 10.1038/s41564-022-01110-2

[9] Ziemert N, Alanjary M, Weber T. The evolution of genome mining in microbes - a review. Nat Prod Rep. 2016;33:988–1005. 27272205 10.1039/c6np00025h

[10] Blin K, Shaw S, Augustijn HE, Reitz ZL, Biermann F, Alanjary M, et al. antiSMASH 7.0: new and improved predictions for detection, regulation, chemical structures and visualisation. Nucleic Acids Res. 2023;51:W46–50. 37140036 10.1093/nar/gkad344 PMC10320115

[11] Navarro-Muñoz JC, Selem-Mojica N, Mullowney MW, Kautsar SA, Tryon JH, Parkinson EI, et al. A computational framework to explore large-scale biosynthetic diversity. Nat Chem Biol. 2020;16:60–8. 31768033 10.1038/s41589-019-0400-9 PMC6917865

[12] Kautsar SA, van der Hooft JJJ, de Ridder D, Medema MH. BiG-SLiCE: a highly scalable tool maps the diversity of 1.2 million biosynthetic gene clusters. Gigascience. 2021;10. Available from: 10.1093/gigascience/giaa154 10.1093/gigascience/giaa154 PMC7804863 33438731

[13] Blin K, Shaw S, Kautsar SA, Medema MH, Weber T. The antiSMASH database version 3: increased taxonomic coverage and new query features for modular enzymes. Nucleic Acids Res. 2021;49:D639–43. 33152079 10.1093/nar/gkaa978 PMC7779067

[14] Steinke K, Mohite OS, Weber T, Kovács ÁT. Phylogenetic distribution of secondary metabolites in the Bacillus subtilis species complex. mSystems. 2021;6. Available from: 10.1128/mSystems.00057-21 10.1128/mSystems.00057-21 PMC8546965 33688015

[15] Mohite OS, Lloyd CJ, Monk JM, Weber T, Palsson BO. Pangenome analysis of Enterobacteria reveals richness of secondary metabolite gene clusters and their associated gene sets. Synth Syst Biotechnol. 2022;7:900–10. 35647330 10.1016/j.synbio.2022.04.011 PMC9125672

[16] Terlouw BR, Blin K, Navarro-Muñoz JC, Avalon NE, Chevrette MG, Egbert S, et al. MIBiG 3.0: a community-driven effort to annotate experimentally validated biosynthetic gene clusters. Nucleic Acids Res. 2023;51:D603–10. 36399496 10.1093/nar/gkac1049 PMC9825592

[17] Nuhamunada M, Mohite OS, Phaneuf PV, Palsson BO, Weber T. BGCFlow: systematic pangenome workflow for the analysis of biosynthetic gene clusters across large genomic datasets. Nucleic Acids Res. 2024;52:5478–95. 38686794 10.1093/nar/gkae314 PMC11162802

[18] Otani H, Udwary DW, Mouncey NJ. Comparative and pangenomic analysis of the genus Streptomyces. Sci Rep. 2022;12:18909. 36344558 10.1038/s41598-022-21731-1 PMC9640686

[19] Caicedo-Montoya C, Manzo-Ruiz M, Ríos-Estepa R. Pan-Genome of the genus Streptomyces and prioritization of biosynthetic gene clusters with potential to produce antibiotic compounds. Front Microbiol. 2021;12: 677558. 34659136 10.3389/fmicb.2021.677558 PMC8510958

[20] Lorenzi J-N, Lespinet O, Leblond P, Thibessard A. Subtelomeres are fast-evolving regions of the Streptomyces linear chromosome. Microb Genom. 2019;7. Available from: 10.1099/mgen.0.000525 10.1099/mgen.0.000525 PMC8627663 33749576

[21] Mira A, Martín-Cuadrado AB, D’Auria G, Rodríguez-Valera F. The bacterial pan-genome: a new paradigm in microbiology. Int Microbiol. 2010;13:45–57. 20890839 10.2436/20.1501.01.110

[22] Jørgensen TS, Mohite OS, Sterndorff EB, Alvarez-Arevalo M, Blin K, Booth TJ, et al. A treasure trove of 1034 actinomycete genomes. Nucleic Acids Res. 2024; Available from: 10.1093/nar/gkae523 10.1093/nar/gkae523 PMC11260486 38908028

[23] Abram K, Udaondo Z, Bleker C, Wanchai V, Wassenaar TM, Robeson MS 2nd, et al. Mash-based analyses of Escherichia coli genomes reveal 14 distinct phylogroups. Commun Biol. 2021;4:117. 33500552 10.1038/s42003-020-01626-5 PMC7838162

[24] Passarelli-Araujo H, Franco GR, Venancio TM. Network analysis of ten thousand genomes shed light on Pseudomonas diversity and classification. Microbiol Res. 2022;254: 126919. 34808515 10.1016/j.micres.2021.126919

[25] Ondov BD, Treangen TJ, Melsted P, Mallonee AB, Bergman NH, Koren S, et al. Mash: fast genome and metagenome distance estimation using MinHash. Genome Biol. 2016;17:132. 27323842 10.1186/s13059-016-0997-x PMC4915045

[26] Hernández-Salmerón JE, Moreno-Hagelsieb G. FastANI, Mash and Dashing equally differentiate between species. PeerJ. 2022;10: e13784. 35891643 10.7717/peerj.13784 PMC9308963

[27] Parks DH, Chuvochina M, Rinke C, Mussig AJ, Chaumeil P-A, Hugenholtz P. GTDB: an ongoing census of bacterial and archaeal diversity through a phylogenetically consistent, rank normalized and complete genome-based taxonomy. Nucleic Acids Res. 2022;50:D785–94. 34520557 10.1093/nar/gkab776 PMC8728215

[28] Chaumeil P-A, Mussig AJ, Hugenholtz P, Parks DH. GTDB-Tk v2: memory friendly classification with the genome taxonomy database. Bioinformatics. 2022;38:5315–6. 36218463 10.1093/bioinformatics/btac672 PMC9710552

[29] Blondel VD, Guillaume J-L, Lambiotte R, Lefebvre E. Fast unfolding of communities in large networks. J Stat Mech. 2008;2008:P10008.

[30] Watson AK, Kepplinger B, Bakhiet SM, Mhmoud NA, Chapman J, Allenby NE, et al. Systematic whole-genome sequencing reveals an unexpected diversity among actinomycetoma pathogens and provides insights into their antibacterial susceptibilities. PLoS Negl Trop Dis. 2022;16: e0010128. 35877680 10.1371/journal.pntd.0010128 PMC9352199

[31] Sánchez-Navarro R, Nuhamunada M, Mohite OS, Wasmund K, Albertsen M, Gram L, et al. Long-read metagenome-assembled genomes improve identification of novel complete biosynthetic gene clusters in a complex microbial activated sludge ecosystem. mSystems. 2022;7:e0063222. 36445112 10.1128/msystems.00632-22 PMC9765116

[32] Tizabi D, Bachvaroff T, Hill RT. Comparative analysis of assembly algorithms to optimize biosynthetic gene cluster identification in novel marine actinomycete genomes. Frontiers in Marine Science. 2022;9. Available from: 10.3389/fmars.2022.914197

[33] Myronovskyi M, Rosenkränzer B, Nadmid S, Pujic P, Normand P, Luzhetskyy A. Generation of a cluster-free Streptomyces albus chassis strains for improved heterologous expression of secondary metabolite clusters. Metab Eng. 2018;49:316–24. 30196100 10.1016/j.ymben.2018.09.004

[34] Seshadri R, Roux S, Huber KJ, Wu D, Yu S, Udwary D, et al. Expanding the genomic encyclopedia of Actinobacteria with 824 isolate reference genomes. Cell Genom. 2022;2: 100213. 36778052 10.1016/j.xgen.2022.100213 PMC9903846

[35] Saati-Santamaría Z. Global map of specialized metabolites encoded in prokaryotic plasmids. Microbiol Spectr. 2023;11: e0152323. 37310275 10.1128/spectrum.01523-23 PMC10434180

[36] Ward AC, Allenby NE. Genome mining for the search and discovery of bioactive compounds: the Streptomyces paradigm. FEMS Microbiol Lett. 2018;365. Available from: 10.1093/femsle/fny240 10.1093/femsle/fny240 30265303

[37] Alanjary M, Steinke K, Ziemert N. AutoMLST: an automated web server for generating multi-locus species trees highlighting natural product potential. Nucleic Acids Res. 2019;47:W276–82. 30997504 10.1093/nar/gkz282 PMC6602446

[38] Booth TJ, Shaw S, Weber T. Getphylo: rapid and automatic generation of multi-locus phylogenetic trees. bioRxiv. 2023. Available from: https://www.biorxiv.org/content/10.1101/2023.07.26.550493.abstract 10.1186/s12859-025-06035-1 PMC11748604 39827349

[39] Tu J, Li S, Chen J, Song Y, Fu S, Ju J, et al. Characterization and heterologous expression of the neoabyssomicin/abyssomicin biosynthetic gene cluster from Streptomyces koyangensis SCSIO 5802. Microb Cell Fact. 2018;17:28. 29463238 10.1186/s12934-018-0875-1 PMC5819245

[40] Cruz-Morales P, Kopp JF, Martínez-Guerrero C, Yáñez-Guerra LA, Selem-Mojica N, Ramos-Aboites H, et al. Phylogenomic analysis of natural products biosynthetic gene clusters allows discovery of arseno-organic metabolites in model streptomycetes. Genome Biol Evol. 2016;8:1906–16. 27289100 10.1093/gbe/evw125 PMC4943196

[41] International Code of Nomenclature of Prokaryotes. Prokaryotic Code (2008 Revision). Int J Syst Evol Microbiol. 2019;69:S1–111. 26596770 10.1099/ijsem.0.000778

[42] Chase AB, Sweeney D, Muskat MN, Guillén-Matus DG, Jensen PR. Vertical inheritance facilitates interspecies diversification in biosynthetic gene clusters and specialized metabolites. MBio. 2021;12: e0270021. 34809466 10.1128/mBio.02700-21 PMC8609351

[43] Fischbach MA, Walsh CT, Clardy J. The evolution of gene collectives: how natural selection drives chemical innovation. Proc Natl Acad Sci U S A. 2008;105:4601–8. 18216259 10.1073/pnas.0709132105 PMC2290807

[44] Ginolhac A, Jarrin C, Robe P, Perrière G, Vogel TM, Simonet P, et al. Type I polyketide synthases may have evolved through horizontal gene transfer. J Mol Evol. 2005;60:716–25. 15909225 10.1007/s00239-004-0161-1

[45] Medema MH, Cimermancic P, Sali A, Takano E, Fischbach MA. A systematic computational analysis of biosynthetic gene cluster evolution: lessons for engineering biosynthesis. PLoS Comput Biol. 2014;10: e1004016. 25474254 10.1371/journal.pcbi.1004016 PMC4256081

[46] Choulet F, Aigle B, Gallois A, Mangenot S, Gerbaud C, Truong C, et al. Evolution of the terminal regions of the Streptomyces linear chromosome. Mol Biol Evol. 2006;23:2361–9. 16956972 10.1093/molbev/msl108

[47] Booth TJ, Bozhüyük KAJ, Liston JD, Batey SFD, Lacey E, Wilkinson B. Bifurcation drives the evolution of assembly-line biosynthesis. Nat Commun. 2022;13:1–12. 35715397 10.1038/s41467-022-30950-z PMC9205934

[48] Adamek M, Alanjary M, Sales-Ortells H, Goodfellow M, Bull AT, Winkler A, et al. Comparative genomics reveals phylogenetic distribution patterns of secondary metabolites in Amycolatopsis species. BMC Genomics. 2018;19:426. 29859036 10.1186/s12864-018-4809-4 PMC5984834

[49] Chevrette MG, Gavrilidou A, Mantri S, Selem-Mojica N, Ziemert N, Barona-Gómez F. The confluence of big data and evolutionary genome mining for the discovery of natural products. Nat Prod Rep. 2021;38:2024–40. 34787598 10.1039/d1np00013f

[50] Parks DH, Imelfort M, Skennerton CT, Hugenholtz P, Tyson GW. CheckM: assessing the quality of microbial genomes recovered from isolates, single cells, and metagenomes. Genome Res. 2015;25:1043–55. 25977477 10.1101/gr.186072.114 PMC4484387

[51] Minh BQ, Schmidt HA, Chernomor O, Schrempf D, Woodhams MD, von Haeseler A, et al. IQ-TREE 2: new models and efficient methods for phylogenetic inference in the genomic era. Mol Biol Evol. 2020;37:1530–4. 32011700 10.1093/molbev/msaa015 PMC7182206

[52] Letunic I, Bork P. Interactive Tree Of Life (iTOL) v4: recent updates and new developments. Nucleic Acids Res. 2019;47:W256–9. 30931475 10.1093/nar/gkz239 PMC6602468

[53] Seemann T. Prokka: rapid prokaryotic genome annotation. Bioinformatics. 2014;30:2068–9. 24642063 10.1093/bioinformatics/btu153

[54] Kautsar SA, Blin K, Shaw S, Navarro-Muñoz JC, Terlouw BR, van der Hooft JJJ, et al. MIBiG 2.0: a repository for biosynthetic gene clusters of known function. Nucleic Acids Res. 2020;48:D454–8. 31612915 10.1093/nar/gkz882 PMC7145714

[55] Shannon P, Markiel A, Ozier O, Baliga NS, Wang JT, Ramage D, et al. Cytoscape: a software environment for integrated models of biomolecular interaction networks. Genome Res. 2003;13:2498–504. 14597658 10.1101/gr.1239303 PMC403769

[56] Gilchrist CLM, Chooi Y-H. clinker & clustermap.js: automatic generation of gene cluster comparison figures. Available from: 10.1101/2020.11.08.370650 10.1093/bioinformatics/btab007 33459763

[57] Booth TJ. Draw dotplots from blastn results. GitHub. 2024. Available from: https://github.com/drboothtj/dotplotter

[58] Nuhamunada M, Mohite OS. BGCFlow: systematic pangenome workflow for the analysis of biosynthetic gene clusters across large genomic datasets. GitHub. 2023. Available from: https://github.com/NBChub/bgcflow 10.1093/nar/gkae314 PMC11162802 38686794

[59] Mohite OS. NBChub/Streptomyces_pangenome_ms. GitHub. 2024. Available from: https://github.com/NBChub/Streptomyces_pangenome_ms

[60] Mohite OS. Pangenome mining of the Streptomyces genus redefines species’ biosynthetic potential. Zenodo. 2024. Available from: https://zenodo.org/records/14310728 10.1186/s13059-024-03471-9 PMC11734326 39810189

