# Supplementary material for: Pangenome mining of the Streptomyces genus redefines species’ biosynthetic potential
Source: Genome Biol. 2025 Jan 14;26:9. doi: 10.1186/s13059-024-03471-9 (PMC11734326; doi:10.1186/s13059-024-03471-9)
Supplement: Supplementary file 2 — Additional file 2. Supplementary figures S1 to S29. Includes supplementary figures and the legends for Fig. S1 to S29. [file 13059_2024_3471_MOESM2_ESM.docx]

Additional file 2 for

Pangenome mining of the *Streptomyces* genus redefines species’ biosynthetic potential

Omkar S. Mohite^1^, Tue S. Jørgensen^1^, Thomas J. Booth^1^, Pep Charusanti^1^, Patrick V. Phaneuf^1^, Tilmann Weber^1#^, Bernhard O. Palsson^1,2#^

^1^The Novo Nordisk Foundation Center for Biosustainability, Technical University of Denmark, Kongens Lyngby 2800, Denmark.

^2^Department of Bioengineering, University of California San Diego, La Jolla, CA 92093, USA.

^#^To whom correspondence should be addressed**.**

Email: [palsson@eng.ucsd.edu](mailto:palsson@eng.ucsd.edu), [tiwe@biosustain.dtu.dk](mailto:tiwe@biosustain.dtu.dk)

Email Addresses:

Omkar S. Mohite: [omkmoh@biosustain.dtu.dk](mailto:omkmoh@biosustain.dtu.dk)

Tue S. Jørgensen: [tuspjo@biosustain.dtu.dk](mailto:tuspjo@biosustain.dtu.dk)

Thomas J. Booth: [thoboo@biosustain.dtu.dk](mailto:thoboo@biosustain.dtu.dk)

Pep Charusanti: [pecha@biosustain.dtu.dk](mailto:pecha@biosustain.dtu.dk)

Patrick V. Phaneuf: [phaneuf@biosustain.dtu.dk](mailto:phaneuf@biosustain.dtu.dk)

Tilmann Weber^#^: [tiwe@biosustain.dtu.dk](mailto:tiwe@biosustain.dtu.dk)

Bernhard O. Palsson^#^: [palsson@eng.ucsd.edu](mailto:palsson@eng.ucsd.edu)

Additional file 2 includes:

Figures S1 to S29

**
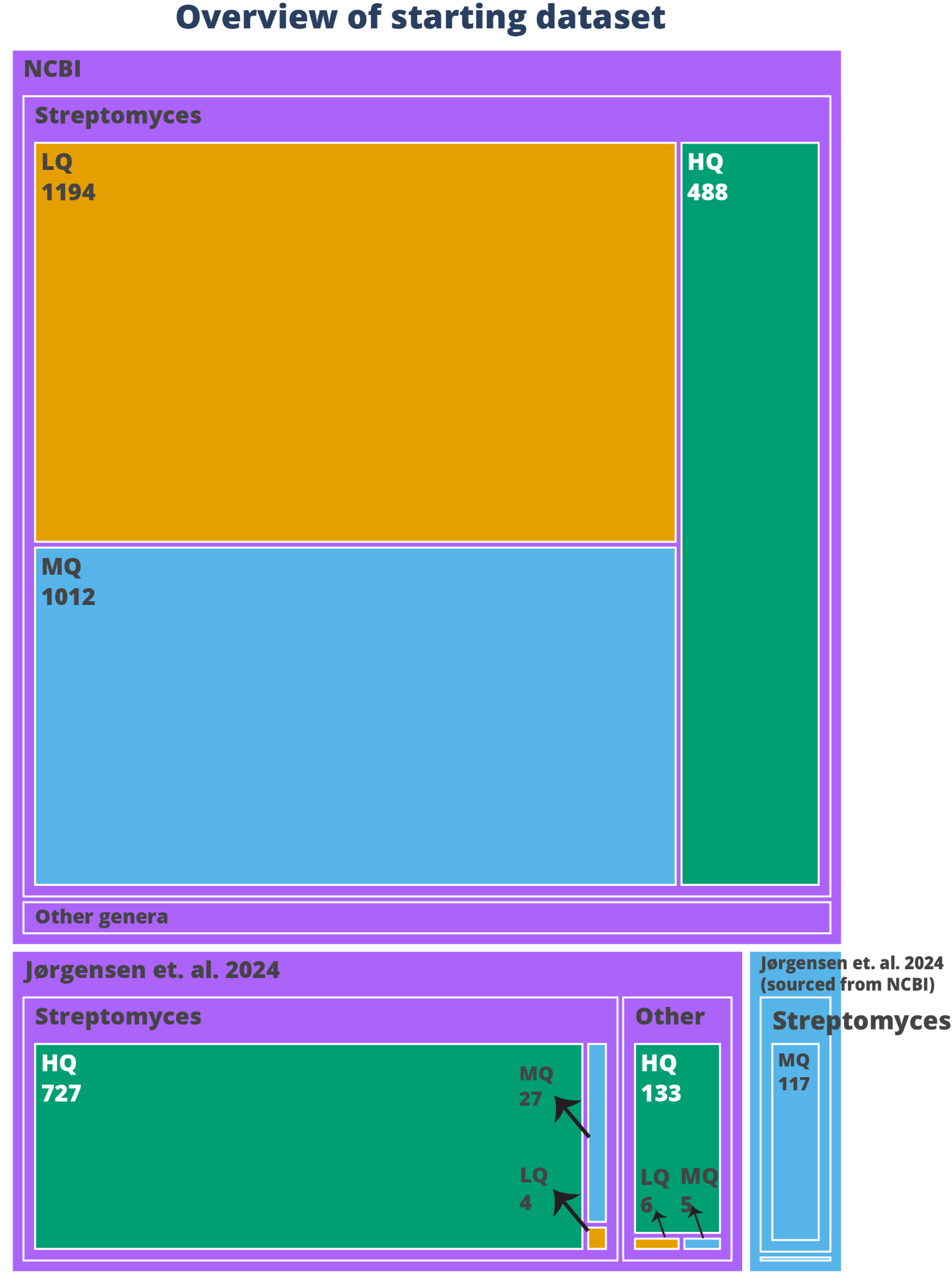
**

**Fig S1. Dataset overview**

Treemap illustrating the number of genomes during various filtering stages. The primary rectangles denote the genome source. Genomes were sourced from NCBI on 30 June, 2023 and our prior study. Part of the genomes form our prior study were already available at NCBI on 30 June, 2023 and were sourced from there. The secondary layer signifies the GTDB-based genus assignment to *Streptomyces*. The tertiary layer classifies genomes by the assembly quality as defined in this study: HQ (High Quality), MQ (Medium Quality), or LQ (Low Quality). HQ: Genomes with complete or chromosome-level assemblies. MQ: Genomes with contig or scaffold level assembly with less than 100 contigs. LQ: Genomes with contig or scaffold level assembly with more than 100 contigs.

**
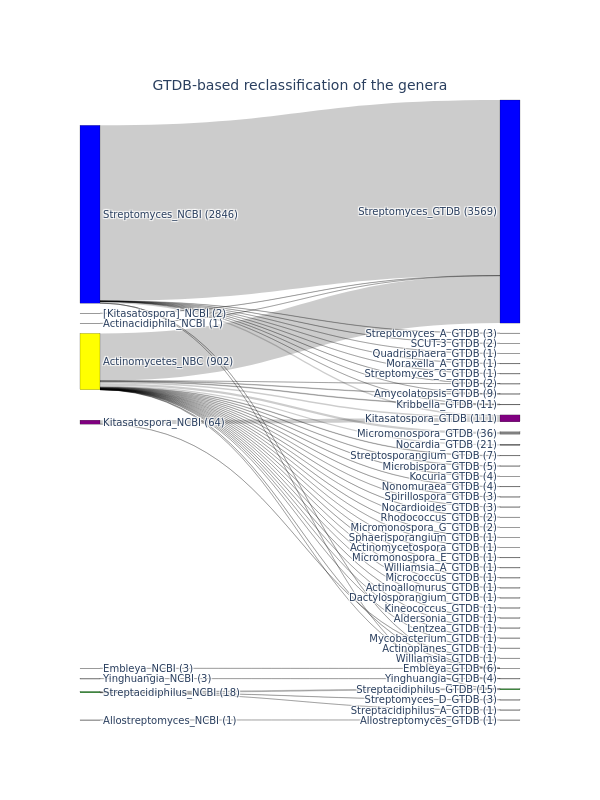
**

**Fig S2. GTDB-based taxonomic assignment**

The genomes of the *Streptomycetaceae* family from NCBI RefSeq and actinomycetes from our recent study (also known as NBC collection) (left) were assigned genus definitions based on GTDB R214 (right). Note that 38 *Streptomyces* genomes were reassigned to different genera using GTDB taxonomy (25 to *Kitasatospora*)

**
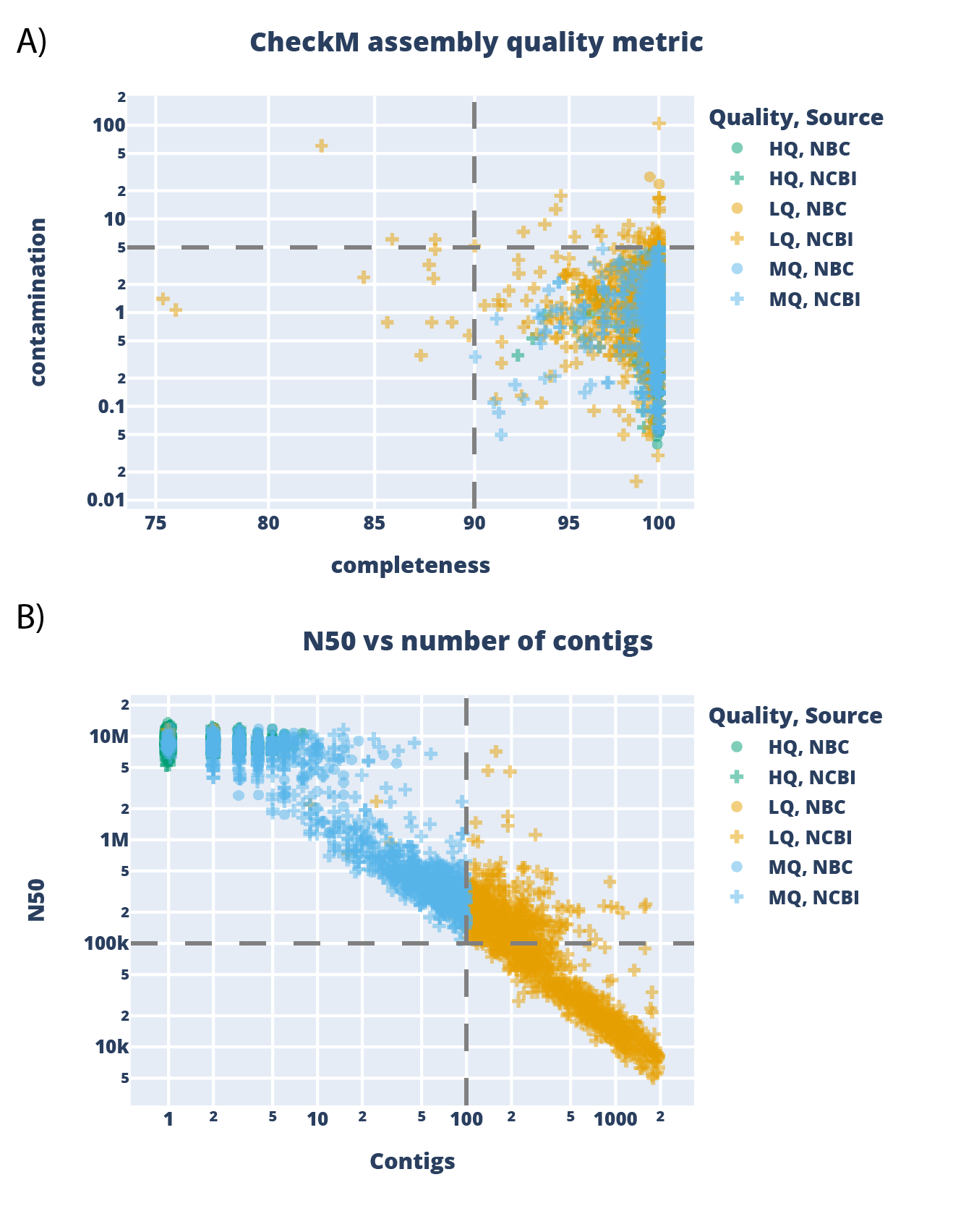
**

**Fig S3. Assembly quality overview and filtering of the dataset**

A) Scatter plot representing the distribution of completeness and contamination score calculated using CheckM across 3,569 *Streptomyces* genomes. Genomes with completeness of less than 90% or contamination of more than 5% were dropped. B) Scatter plot representing the distribution of N50 score and number of contigs across 3,569 *Streptomyces* genomes. The colors represent the quality (HQ, MQ, or LQ) whereas the shapes represent the source of the genome (NCBI or NBC). HQ: Genomes with complete or chromosome level assemblies. MQ: Genomes with contig or scaffold level assembly with less than 100 contigs. LQ: Genomes with contig or scaffold level assembly with more than 100 contigs.


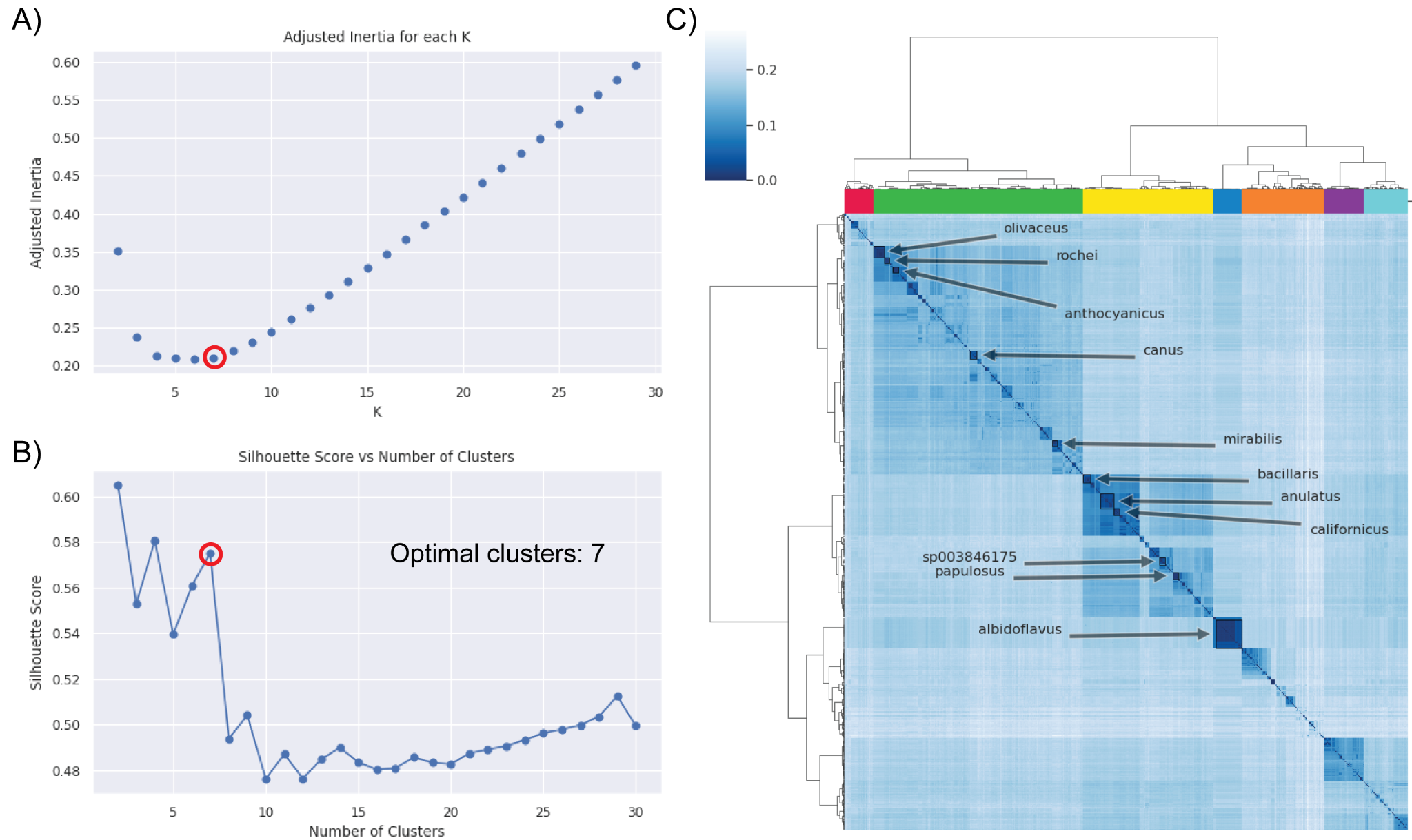


**Fig S4. Detection of optimal clusters using K-means and Silhouette scores on the curated dataset of 2371 genomes**

A) Adjusted inertia against different K-means clusters representing optimal clustering with 7 Mash-clusters. B) Average Silhouette score of all samples for different numbers of clusters showing 7 optimal Mash-clusters. C) Hierarchical dendrogram with clustermap representing Mash distance values across genomes. The column colors represent the 7 optimal Mash-clusters. The top 20 abundant species are highlighted in the clustermap.


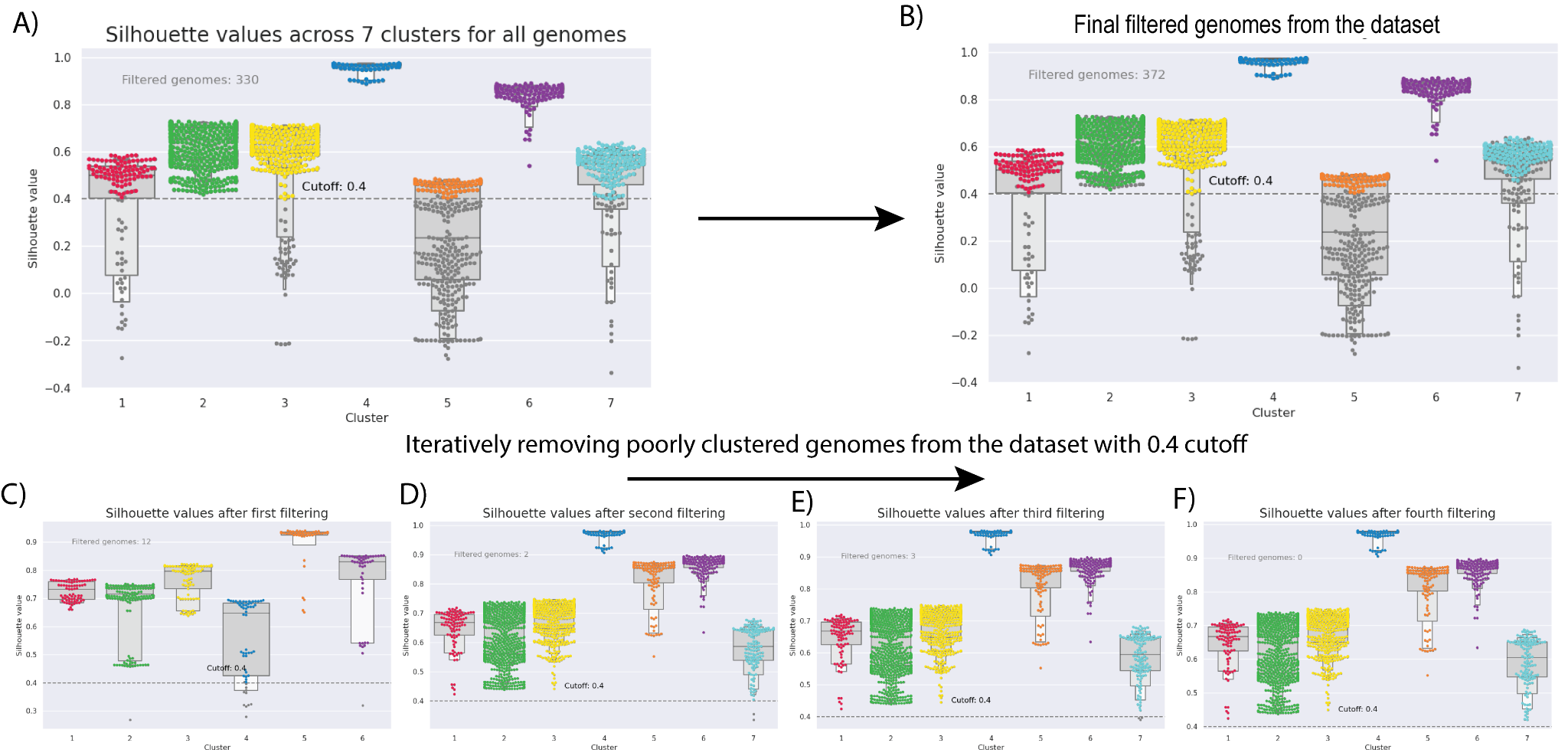


**Fig S5. Iterative filtering of poorly clustered genomes using Silhouette score cutoff**

A) Swarmplot representing silhouette values of each sample genome across the 7 predicted Mash-clusters. The genomes with silhouette values lower than 0.4 are removed to detect the clusters accurately. C) to F) Iteratively reducing the size of the dataset until all samples have silhouette values higher than 0.4. B) Final dataset of 1999 genome samples plotted on the original clustering in panel A. The color of the dots represents one of the seven predicted primary Mash-clusters with grey color denoting the poorly clustered sample genomes that are filtered out.

**
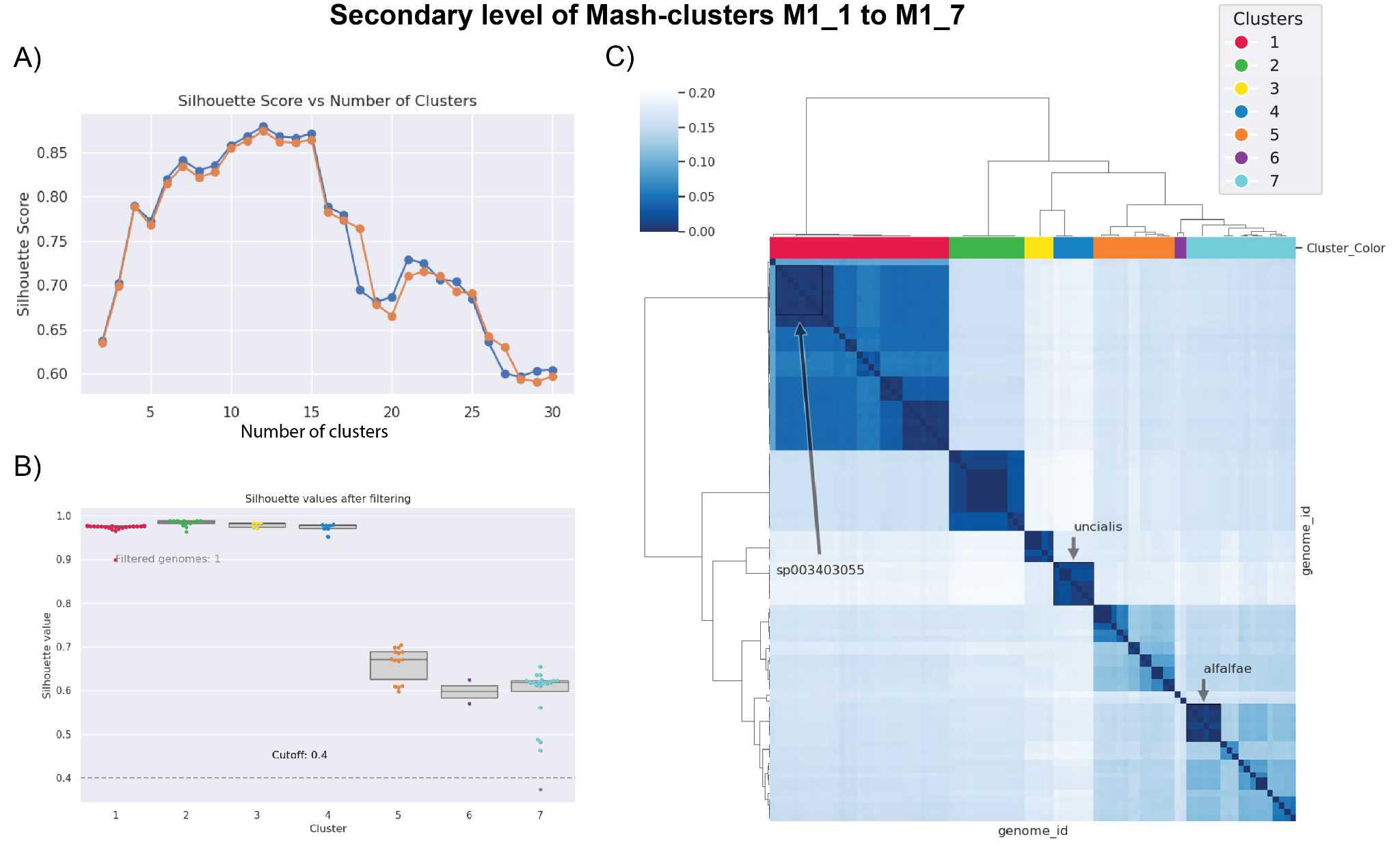
**

**Fig S6. Detection of secondary Mash-clusters using Silhouette scores within the M1 primary Mash-cluster**

A) The average silhouette scores of all samples against the number of defined clusters with hierarchical clustering based on the Mash distance matrix. The orange line plot represents the original dataset of M1 Mash-cluster genomes whereas the blue represents the dataset after removing poorly clustered samples. B) The silhouette scores of each sample across 7 secondary Mash-clusters. The cutoff of 0.4 was used to select the samples with good clustering. The grey dots represent 1 genome that was removed from the clustering analysis. C) Heatmap representing the Mash distances between the genomes from the refined dataset. The rows and columns are clustered using the hierarchical clustering method where the colors on columns represent the 7 secondary Mash-clusters. The highlighted text on the heatmap represents some of the abundant species.

**
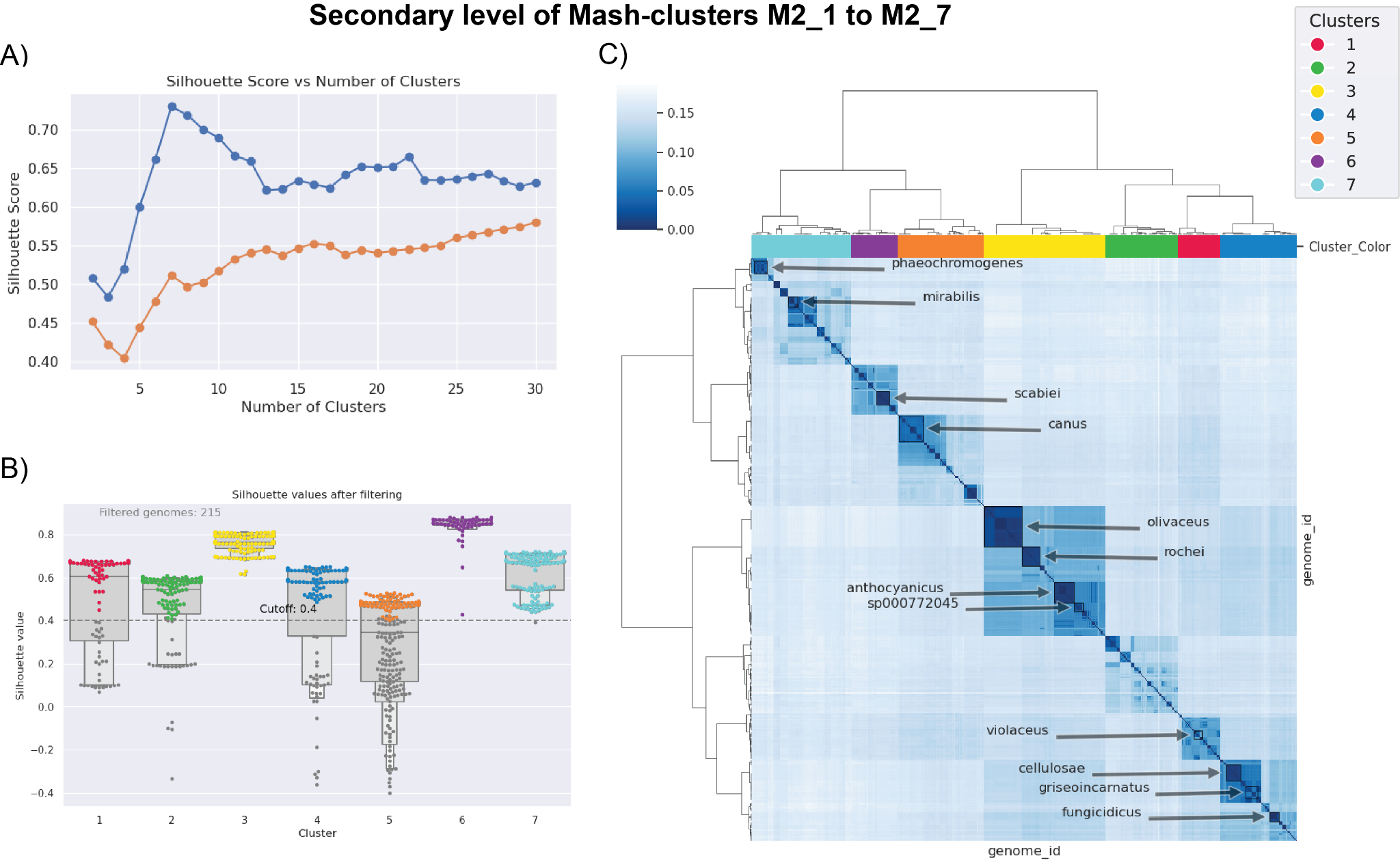
**

**Fig S7. Detection of secondary Mash-clusters using Silhouette scores within the M2 primary Mash-cluster**

A) The average silhouette scores of all samples against the number of defined clusters with hierarchical clustering based on the Mash distance matrix. The orange line plot represents the original dataset of M2 Mash-cluster genomes whereas the blue represents the dataset after removing poorly clustered samples. B) The silhouette scores of each sample across 7 secondary Mash-clusters. The cutoff of 0.4 was used to select the samples with good clustering. The grey dots represent 215 genomes that were removed from the clustering analysis. C) Heatmap representing the Mash distances between the genomes from the refined dataset. The rows and columns are clustered using the hierarchical clustering method where the colors on columns represent the 7 secondary Mash-clusters. The highlighted text on the heatmap represents some of the abundant species. Note: *S. anthocyanicus* is the renamed species of *S. coelicolor* as per GTDB.

**
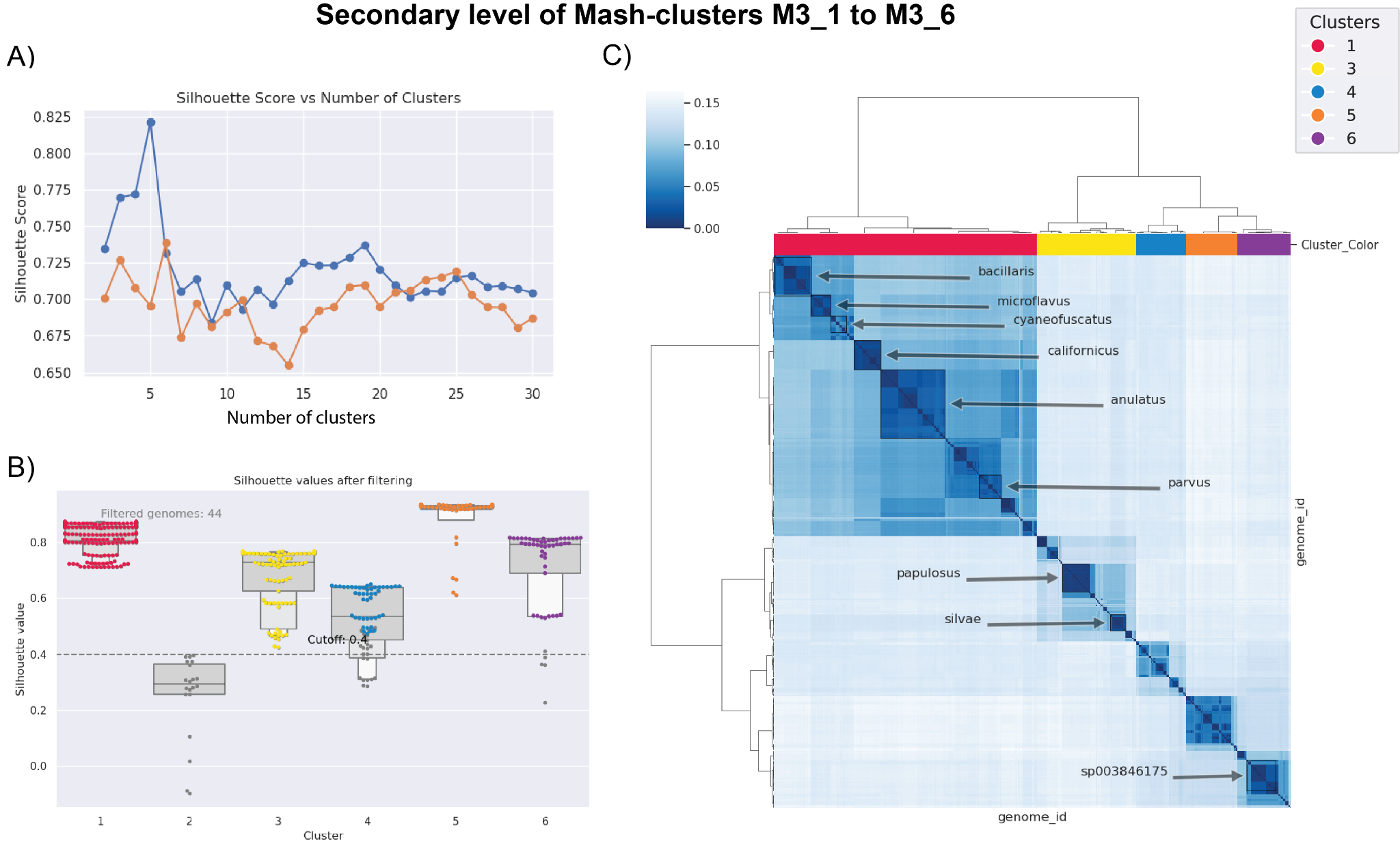
**

**Fig S8. Detection of secondary Mash-clusters using Silhouette scores within the M3 primary Mash-cluster**

A) The average silhouette scores of all samples against the number of defined clusters with hierarchical clustering based on the Mash distance matrix. The orange line plot represents the original dataset of M3 Mash-cluster genomes whereas the blue represents the dataset after removing poorly clustered samples. B) The silhouette scores of each sample across 6 secondary Mash-clusters. The cutoff of 0.4 was used to select the samples with good clustering. The grey dots represent 44 genomes that were removed from the clustering analysis, including an entire cluster 2. C) Heatmap representing the Mash distances between the genomes from the refined dataset. The rows and columns are clustered using the hierarchical clustering method where the colors on columns represent the 5 secondary Mash-clusters (note that cluster 2 was completely removed). The highlighted text on the heatmap represents some of the abundant species.


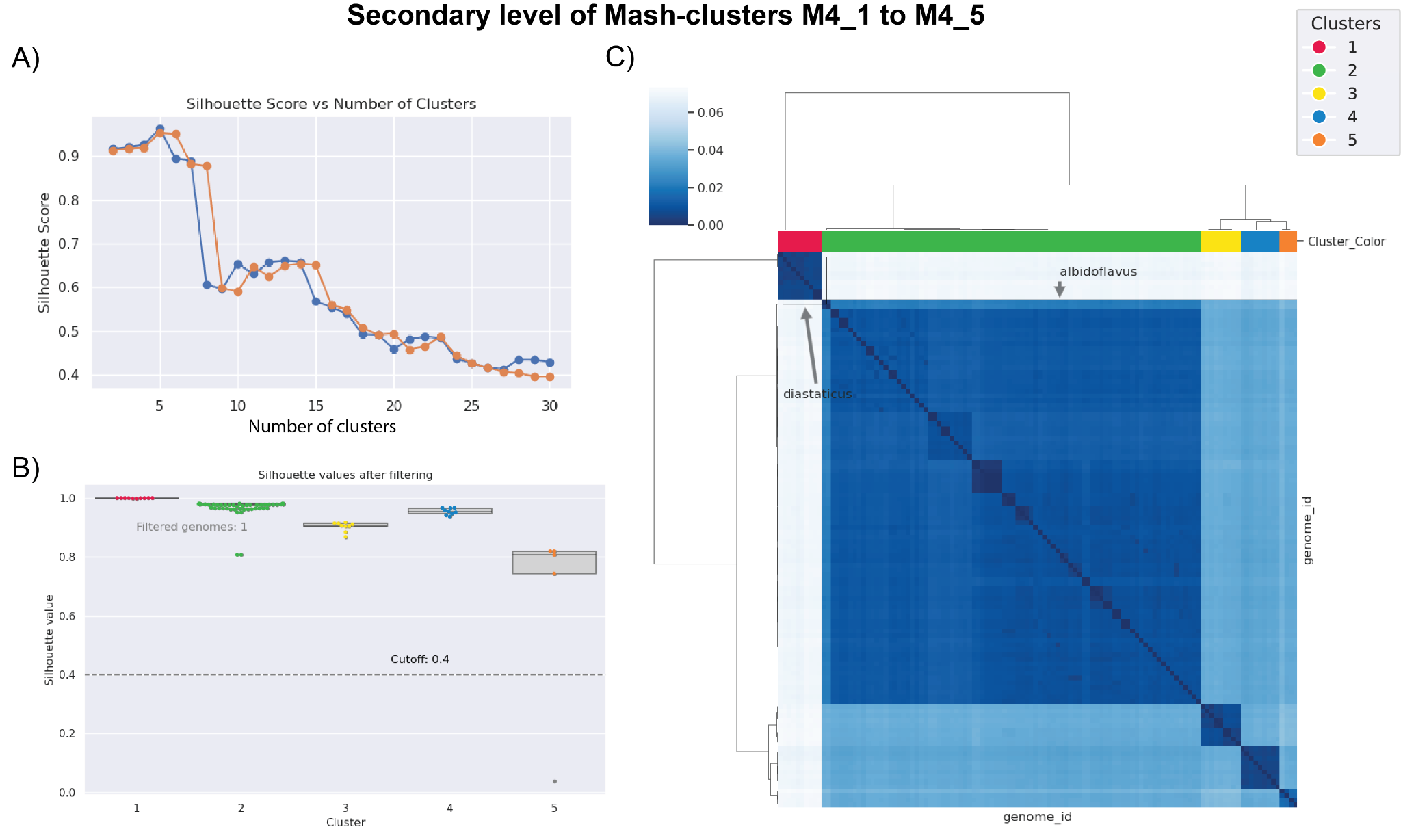


**Fig S9. Detection of secondary Mash-clusters using Silhouette scores within the M4 primary Mash-cluster**

A) The average silhouette scores of all samples against the number of defined clusters with hierarchical clustering based on the Mash distance matrix. The orange line plot represents the original dataset of M4 Mash-cluster genomes whereas the blue represents the dataset after removing poorly clustered samples. B) The silhouette scores of each sample across 5 secondary Mash-clusters. The cutoff of 0.4 was used to select the samples with good clustering. The grey dots represent 1 genome that was removed from the clustering analysis. C) Heatmap representing the Mash distances between the genomes from the refined dataset. The rows and columns are clustered using the hierarchical clustering method where the colors on columns represent the 5 secondary Mash-clusters. The highlighted text on the heatmap represents some of the abundant species including *S. albidoflavus* as a major contributor of the M4 Mash-cluster.


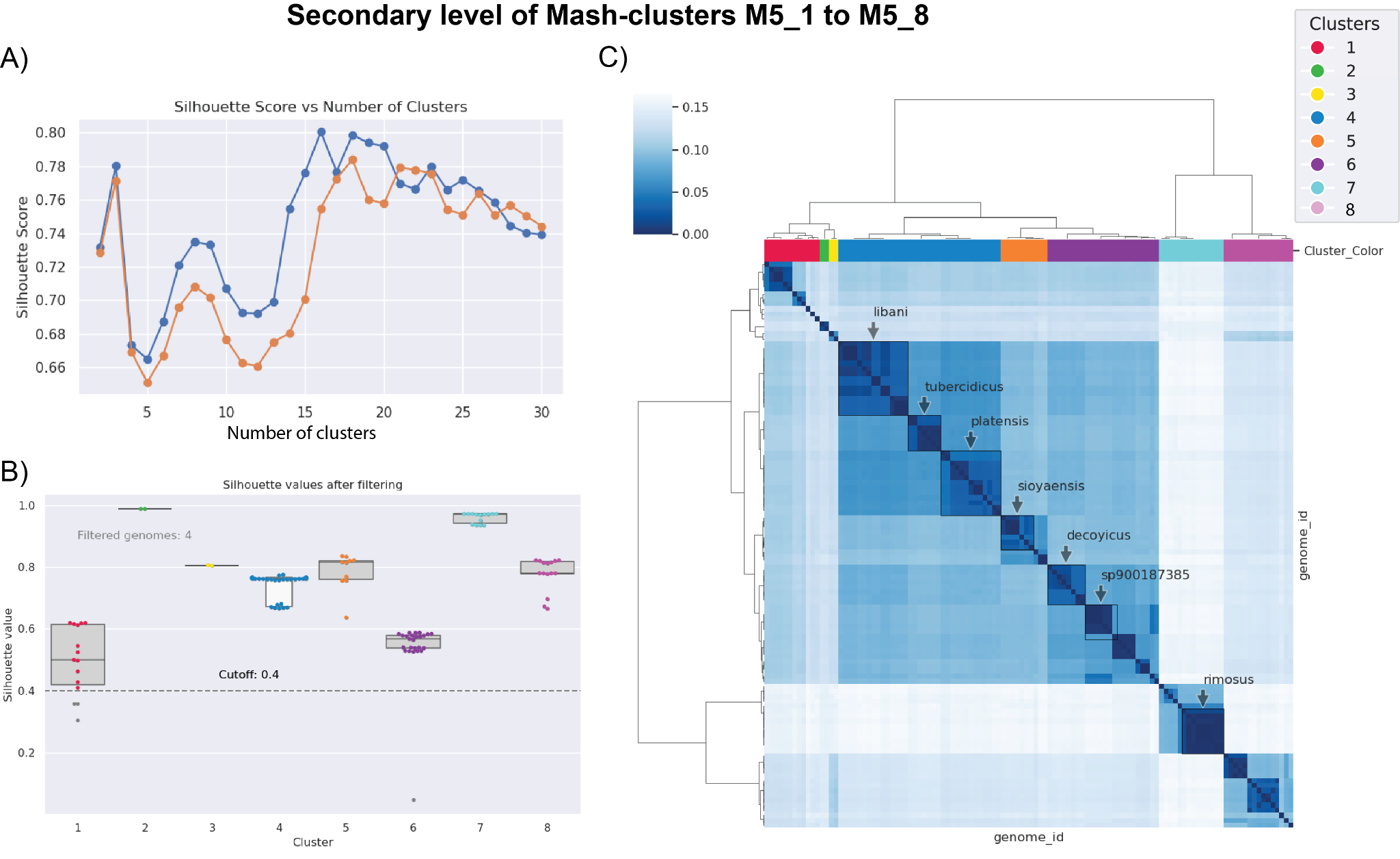


**Fig S10. Detection of secondary Mash-clusters using Silhouette scores within the M5 primary Mash-cluster**

A) The average silhouette scores of all samples against the number of defined clusters with hierarchical clustering based on the Mash distance matrix. The orange line plot represents the original dataset of M5 Mash-cluster genomes whereas the blue represents the dataset after removing poorly clustered samples. B) The silhouette scores of each sample across 8 secondary Mash-clusters. The cutoff of 0.4 was used to select the samples with good clustering. The grey dots represent 4 genomes that were removed from the clustering analysis. C) Heatmap representing the Mash distances between the genomes from the refined dataset. The rows and columns are clustered using the hierarchical clustering method where the colors on columns represent the 8 secondary Mash-clusters. The highlighted text on the heatmap represents some of the abundant species. Note that the M5 Mash-cluster is one of the most diverse and likely poorly sampled in the dataset.

**
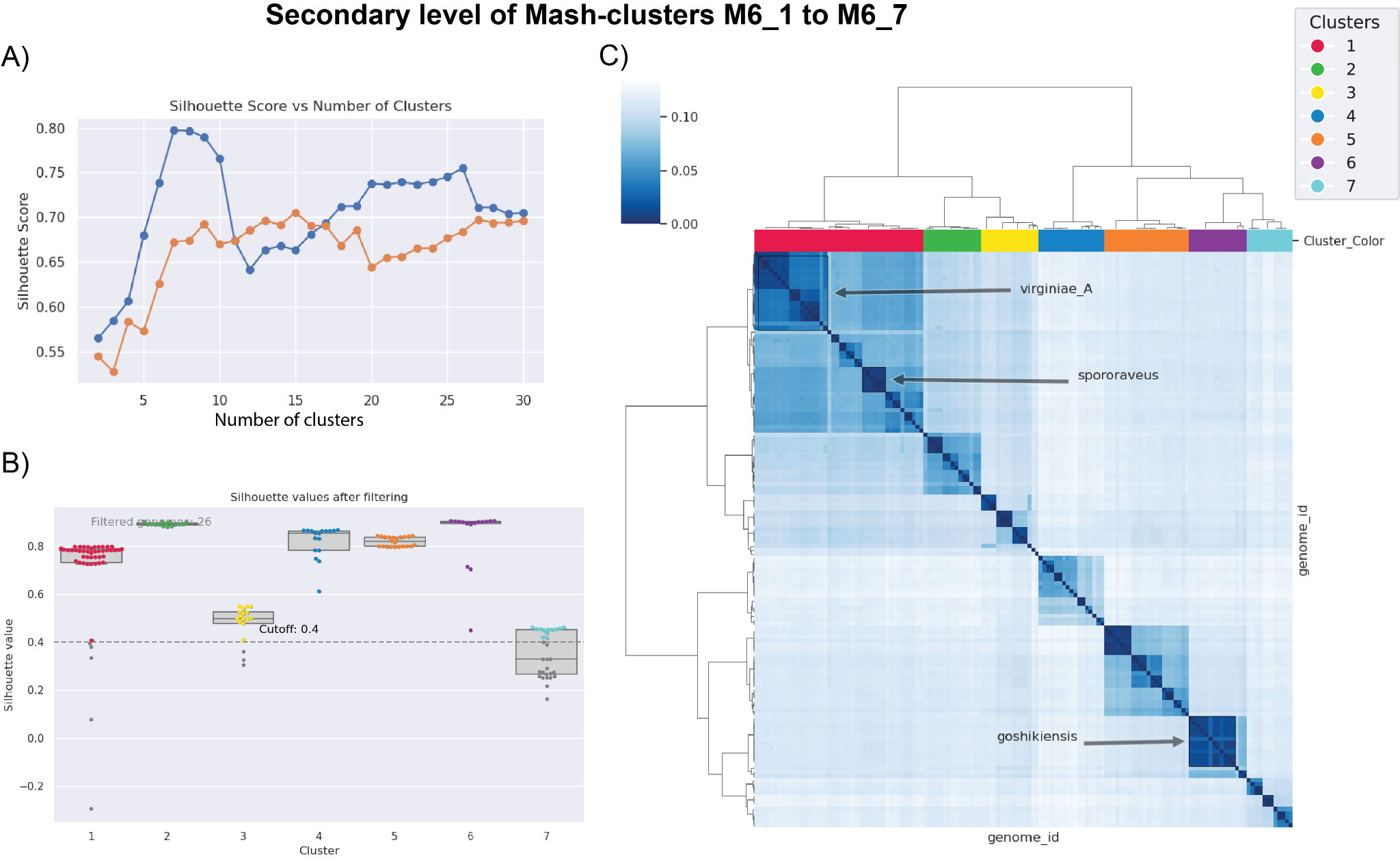
**

**Fig S11. Detection of secondary Mash-clusters using Silhouette scores within the M6 primary Mash-cluster**

A) The average silhouette scores of all samples against the number of defined clusters with hierarchical clustering based on the Mash distance matrix. The orange line plot represents the original dataset of M6 Mash-cluster genomes whereas the blue represents the dataset after removing poorly clustered samples. B) The silhouette scores of each sample across 7 secondary Mash-clusters. The cutoff of 0.4 was used to select the samples with good clustering. The grey dots represent 26 genomes that were removed from the clustering analysis. C) Heatmap representing the Mash distances between the genomes from the refined dataset. The rows and columns are clustered using the hierarchical clustering method where the colors on columns represent the 7 secondary Mash-clusters. The highlighted text on the heatmap represents some of the abundant species.

**
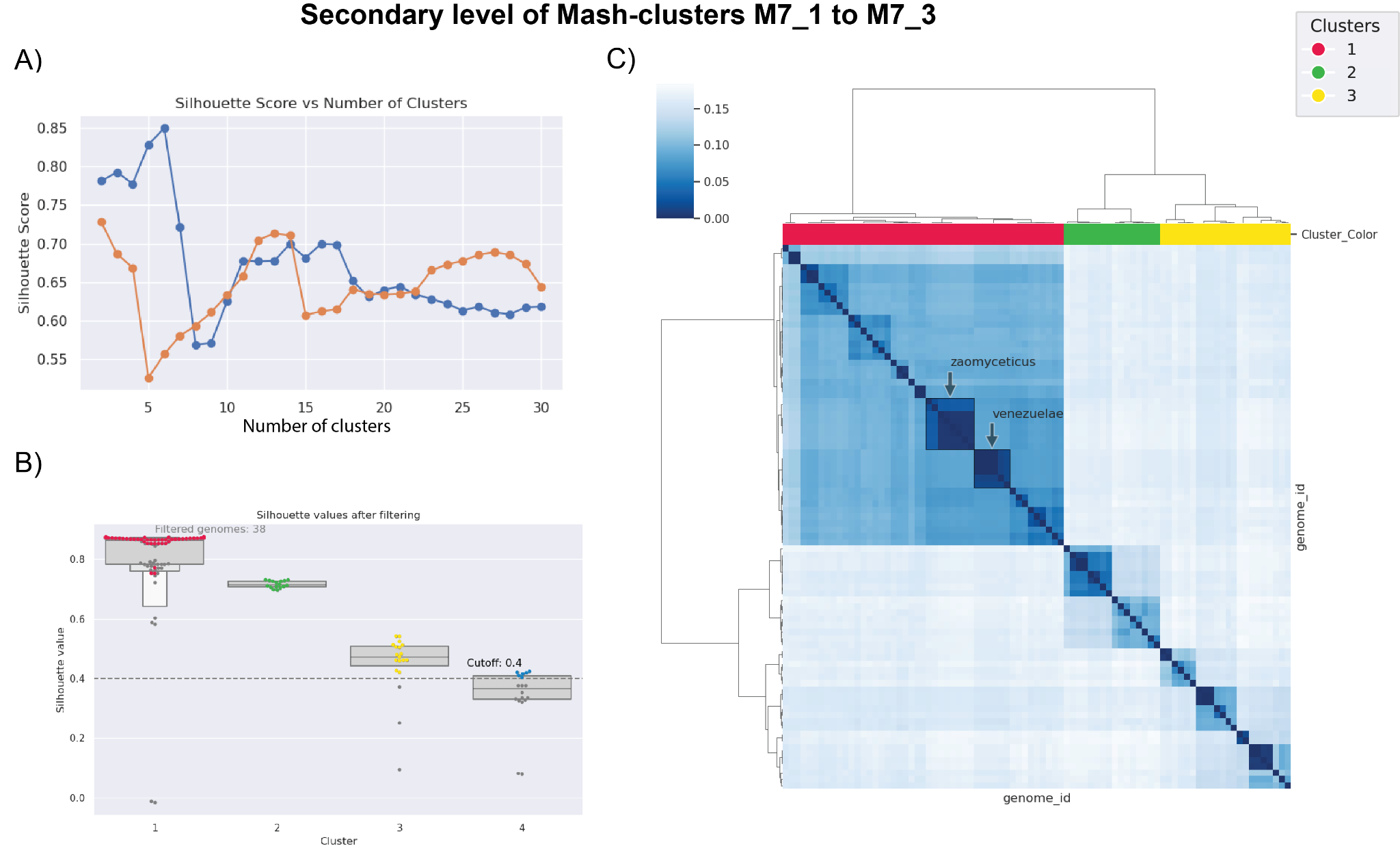
**

**Fig S12. Detection of secondary Mash-clusters using Silhouette scores within the M7 primary Mash-cluster**

A) The average silhouette scores of all samples against the number of defined clusters with hierarchical clustering based on the Mash distance matrix. The orange line plot represents the original dataset of M7 Mash-cluster genomes whereas the blue represents the dataset after removing poorly clustered samples. B) The silhouette scores of each sample across 4 secondary Mash-clusters. The cutoff of 0.4 was used to select the samples with good clustering. The grey dots represent 38 genomes that were removed from the clustering analysis. C) Heatmap representing the Mash distances between the genomes from the refined dataset (note that 3 clusters were generated in the refined dataset). The rows and columns are clustered using the hierarchical clustering method where the colors on columns represent the 3 secondary Mash-clusters. The highlighted text on the heatmap represents some of the abundant species.


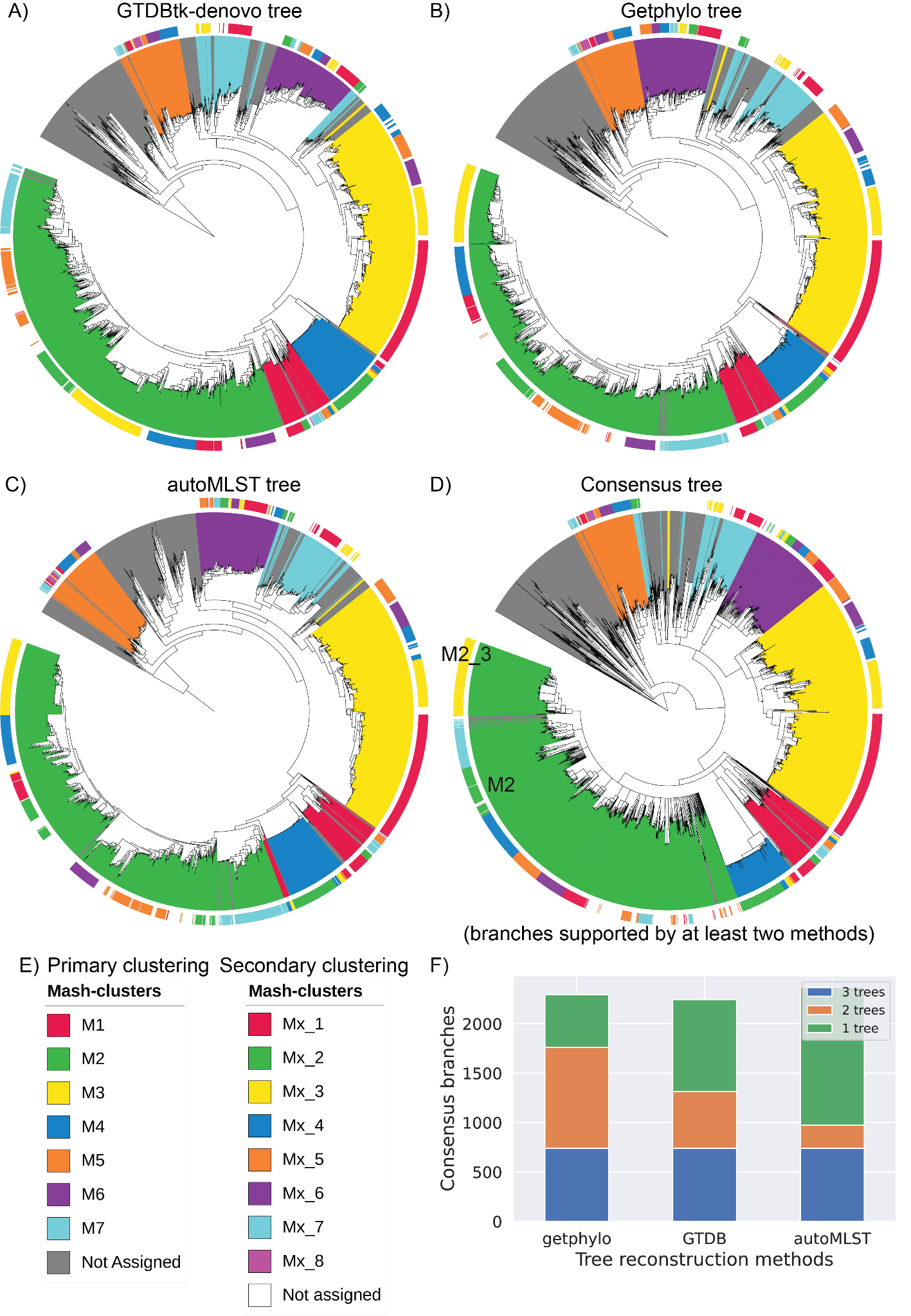


**Fig S13. Comparative assessment of Mash-clusters aligned against different phylogenetic trees**

Phylogenetic trees were reconstructed using 3 different methods: A) GTDB-Tk denovo, B) getphylo, and C) autoMLST. D) A consensus tree generated from the getphylo tree where the branches supported in at least two trees were kept. E) Color legend representing the primary and secondary level of Mash-clusters. For example, M2 and M2_3 are highlighted in panel D. F) Number of branches in individual trees showing the consensus across the other trees, with getphylo showing maximum number of consensus branches.


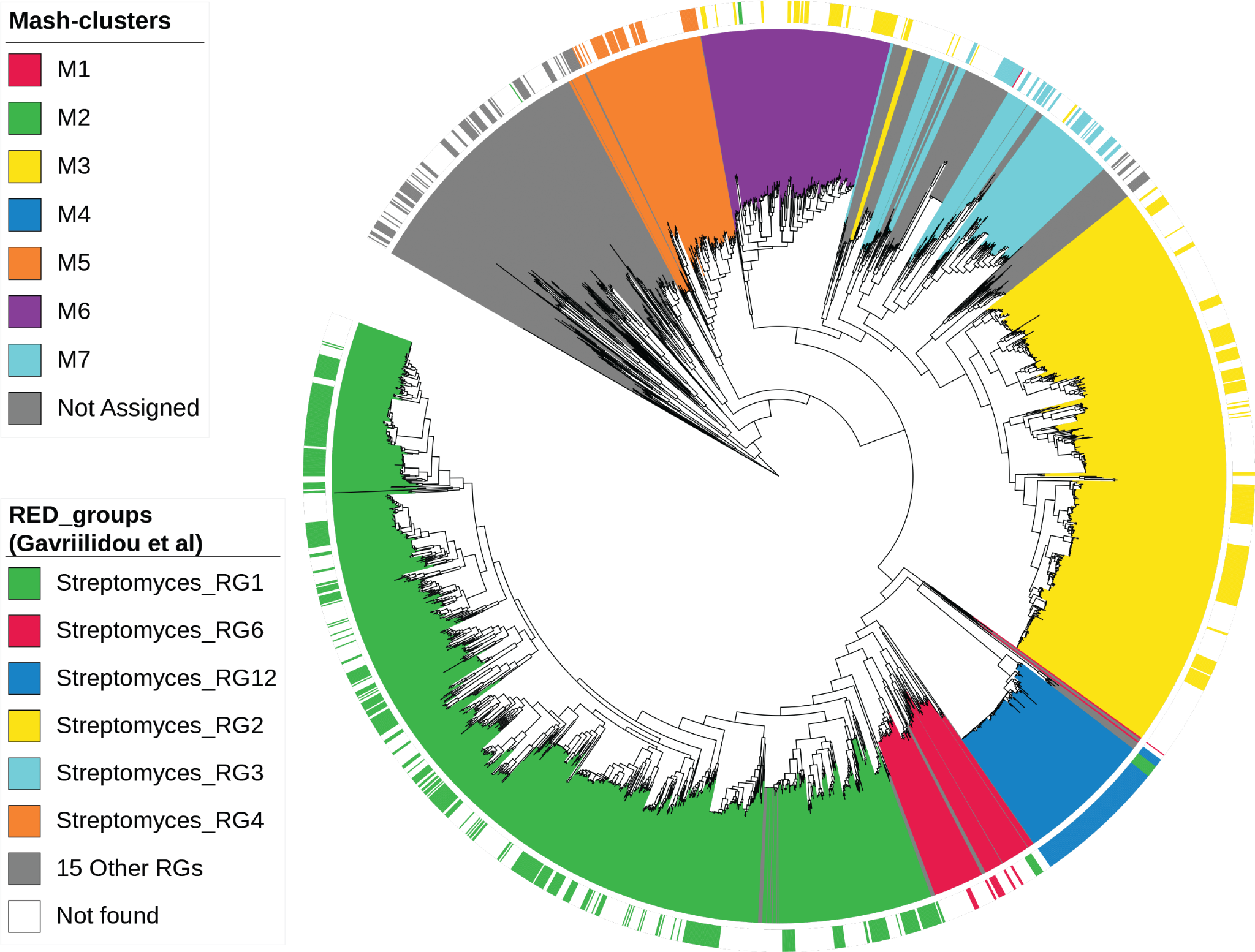


**Fig S14: Comparison of Mash-clusters with groups proposed by Gavriilidou et. al.**

The RED (relative evolutionary divergence) groups as defined by Gavriilidou et. al. were mapped to the consensus tree and the Mash-clusters. The top 6 RED groups are represented by different colors on the external strip with the remaining RED groups colored in grey. The GTDB species that were not part of the earlier study are ignored on color strip.


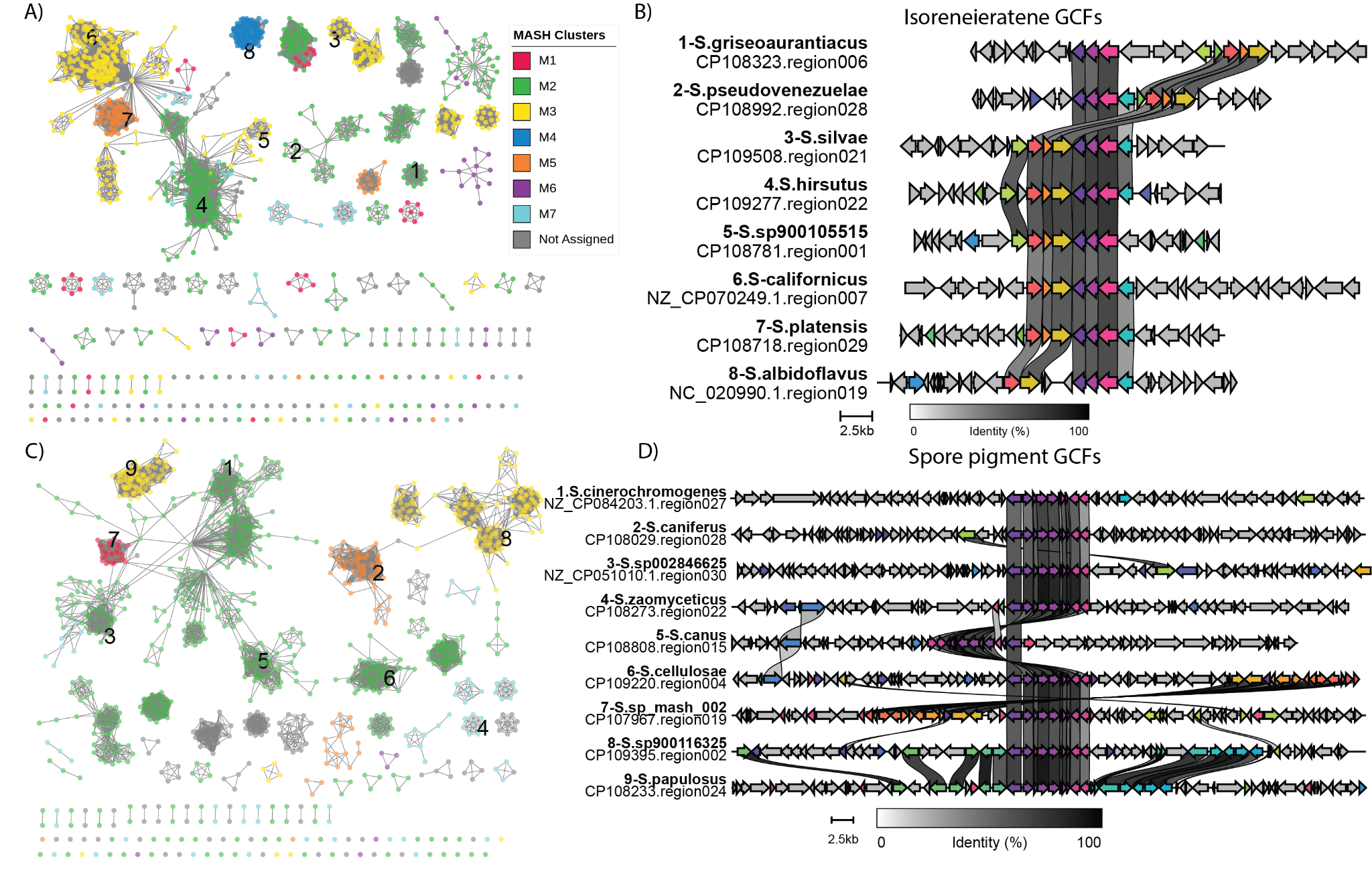


**Fig S15. Distribution and variation within common GCFs**

A) Similarity network based on BiG-SCAPE indicating detection of different GCFs for BGCs with hits against a common MIBiG entry of isorenieratene. B) The alignment of selected BGCs from panel A (highlighted with numbers) indicates mostly conserved core biosynthetic genes with variations arising from extended cluster boundary definitions. C) Similarity network based on BiG-SCAPE indicating detection of different GCFs for BGCs with hits against a common MIBiG entry of spore pigment. D) The alignment of selected BGCs from panel C (highlighted with numbers) again indicating highly conserved core biosynthetic genes with variations arising from extended cluster boundary definitions.

**
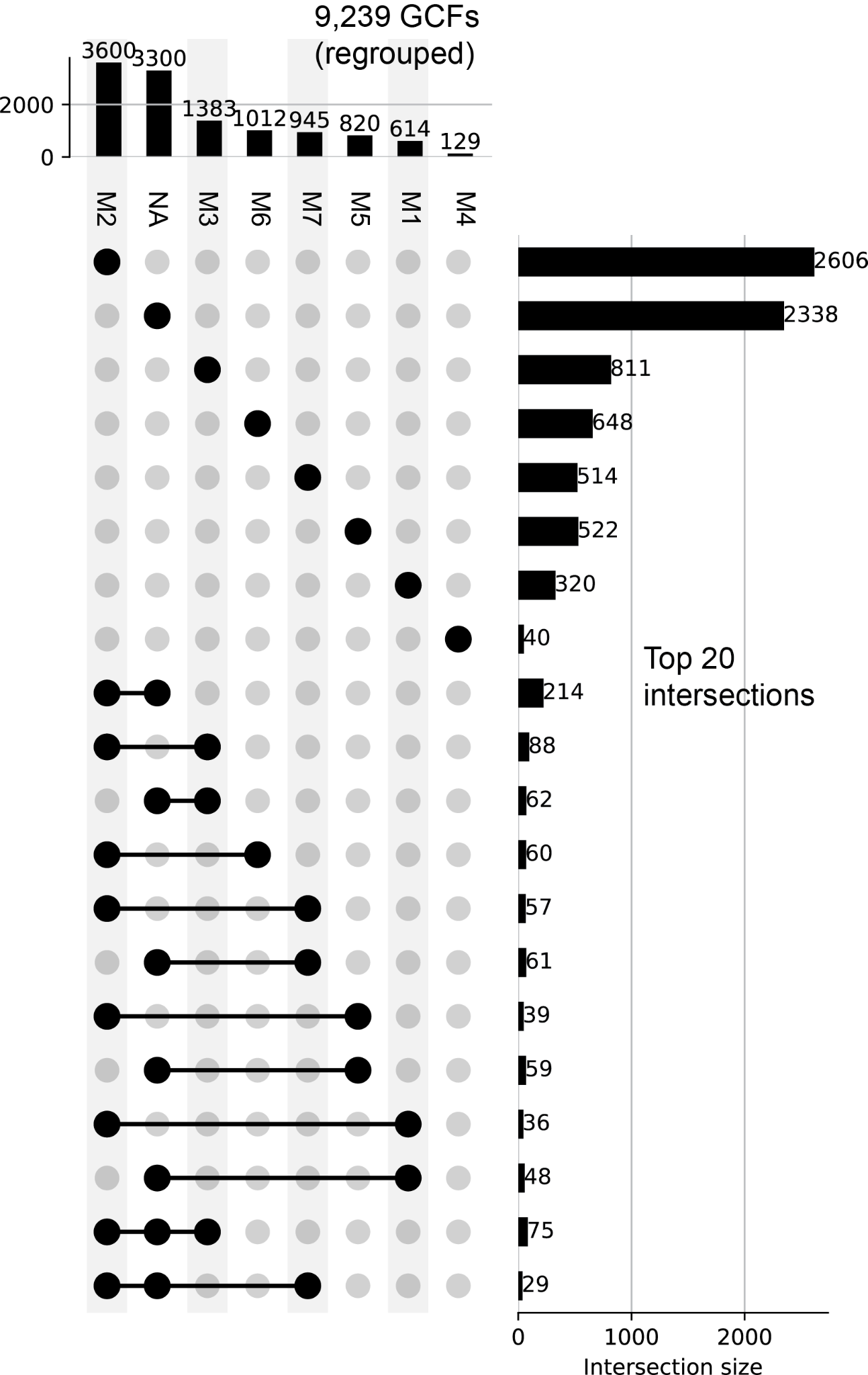
**

**Fig S16. UpSet plot representation of GCFs present across different primary Mash-clusters**

The ‘*NA’* category is represented by genomes with no Mash-cluster assigned. The top 20 most abundant intersections are selected for the visualization. The bars along the top represent total GCFs present in each Mash-cluster. The bars along the right represent the number of GCFs in the corresponding intersection.

**
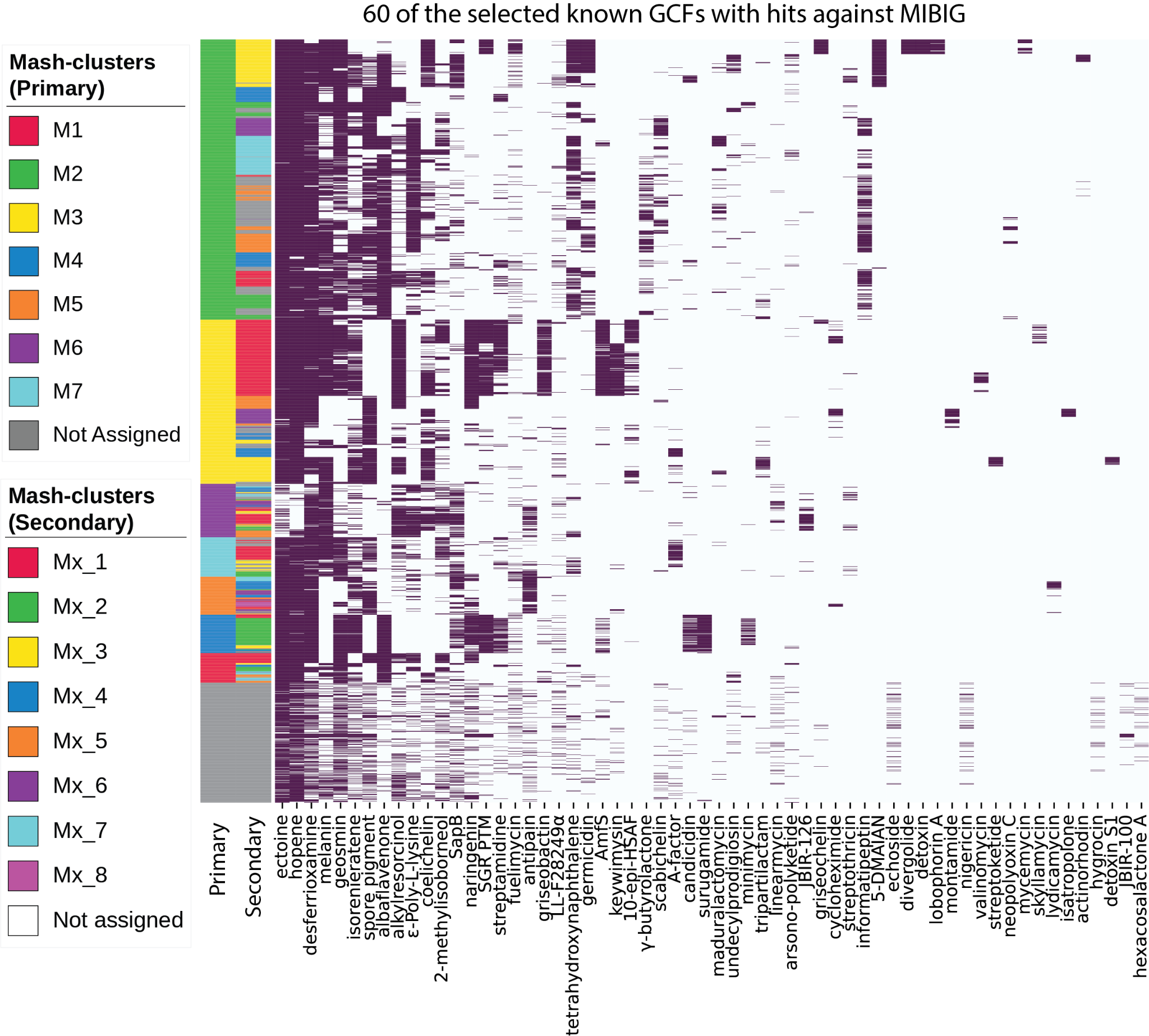
**

**Fig S17: The presence-absence heatmap of GCFs with knownclusterblast similarity hits against the MIBiG database**

The top 20 GCFs were selected from each of the three categories: present in more than 6 Mash-clusters, present in 2 to 6 Mash-clusters, and present in only one of the Mash-clusters. The row colors represent Mash-cluster assignment at both primary and secondary levels. The secondary Mash-cluster colors are assigned within each primary Mash-cluster. For example, M4_1 to M4_5 are assigned the colors of Mx_1 to Mx_5 in the legend for secondary Mash-clusters.


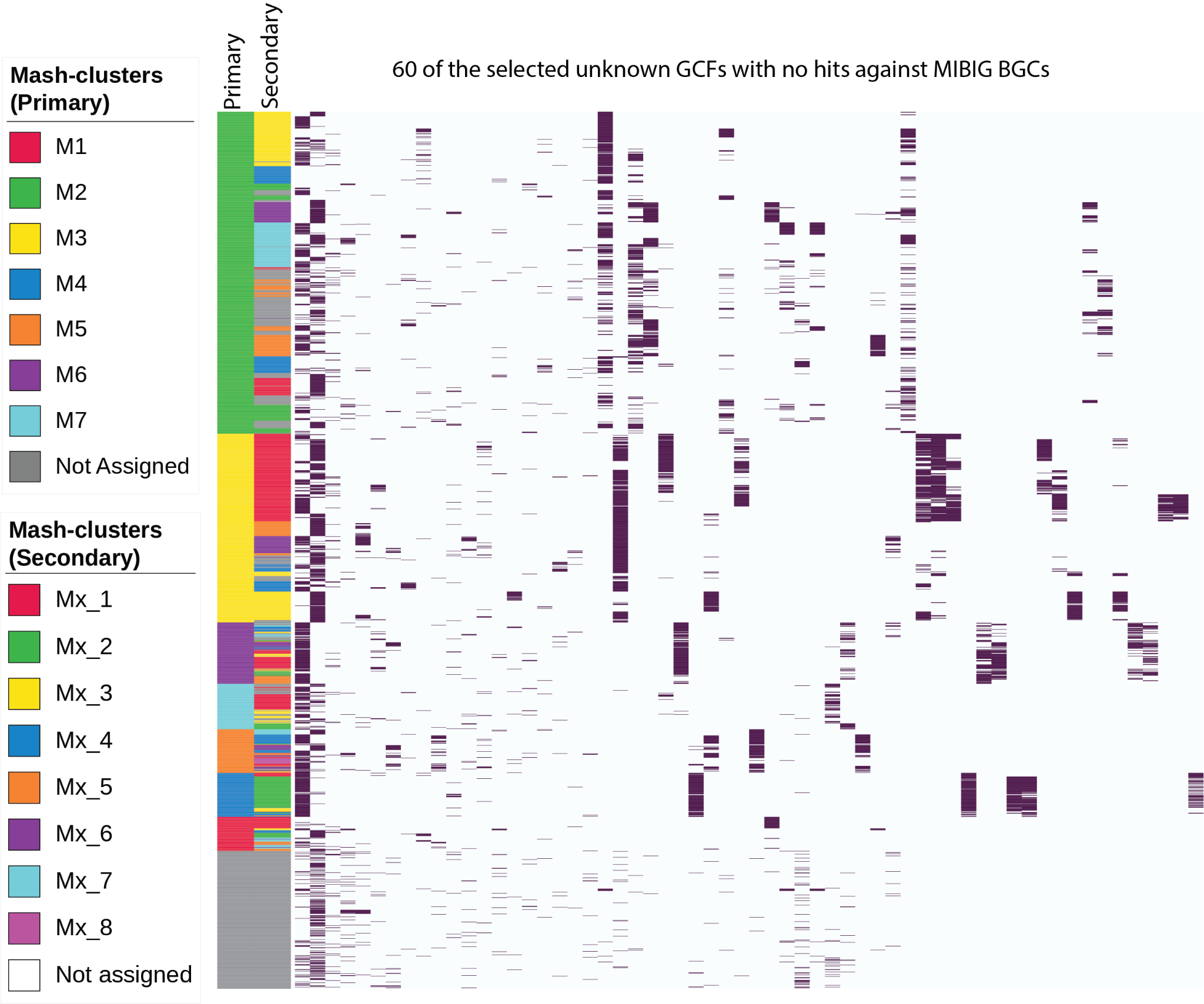


**Fig S18. The presence-absence heatmap of GCFs without knownclusterblast similarity hits against the MIBiG database**

The top 20 GCFs were selected from each of the three categories: present in more than 6 Mash-clusters, present in 2 to 6 Mash-clusters, and present in one of the Mash-clusters. The row colors represent Mash-cluster assignment at both primary and secondary levels. The secondary Mash-cluster colors are assigned within each primary Mash-cluster. For example, M4_1 to M4_5 are assigned the colors of Mx_1 to Mx_5 in the legend for secondary Mash-clusters.


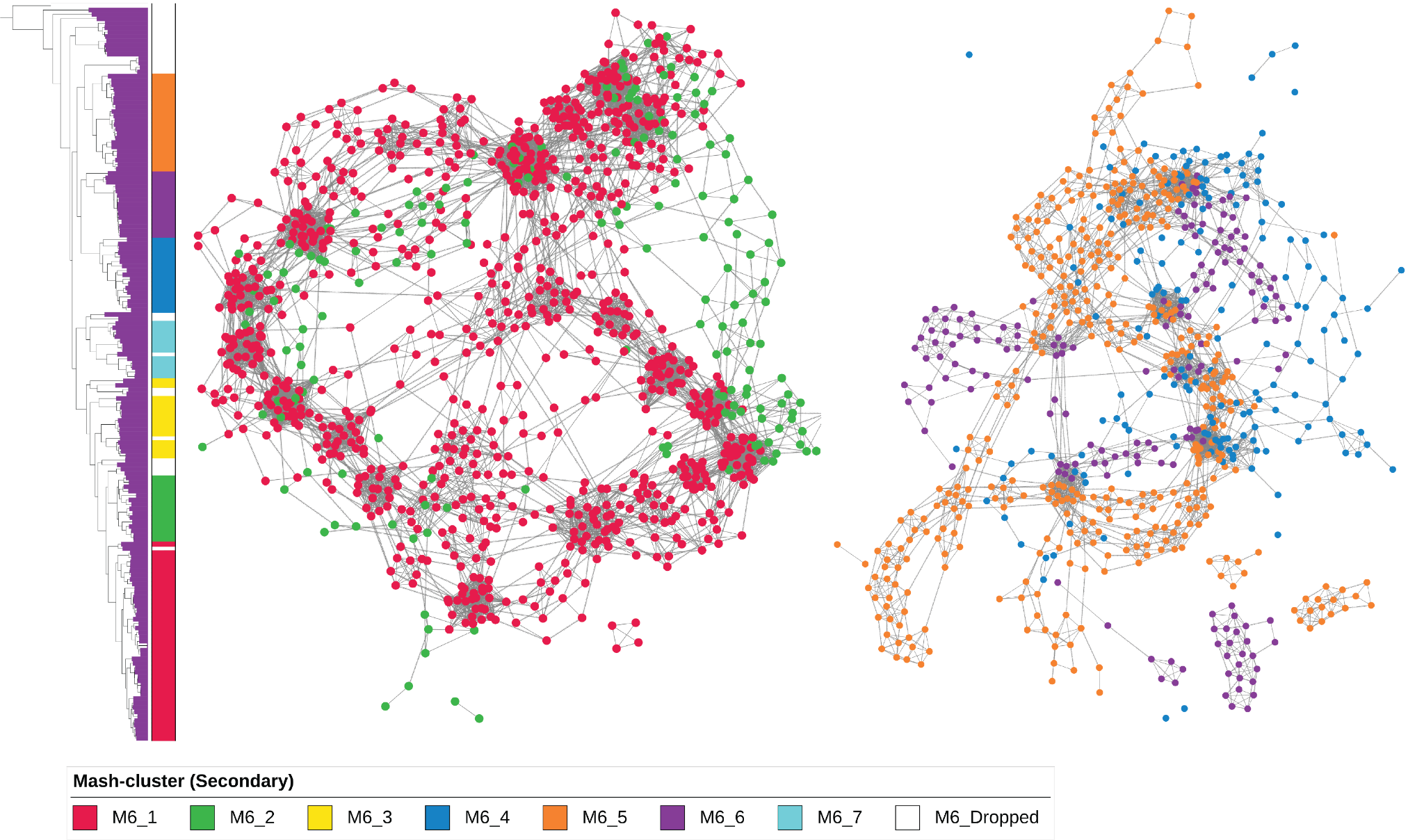


**Fig S19. Enriched similarity network of multiple closely related secondary Mash-clusters**

Different colors of nodes represent secondary Mash-clusters from M6 which are also shown on the phylogenetic tree by the color bar. The similarity network of neighbouting Mash-clusters M6_1, M6_2 (on the left panel) and M6_4, M6_5, M6_6 (on the right panel) show BGCs conserved within and across Mash-clusters. We note that Mash-clusters M6_3 and M6_7 had some of the lowest silhouette scores (Fig S11) and thus were not included in this figure.

.
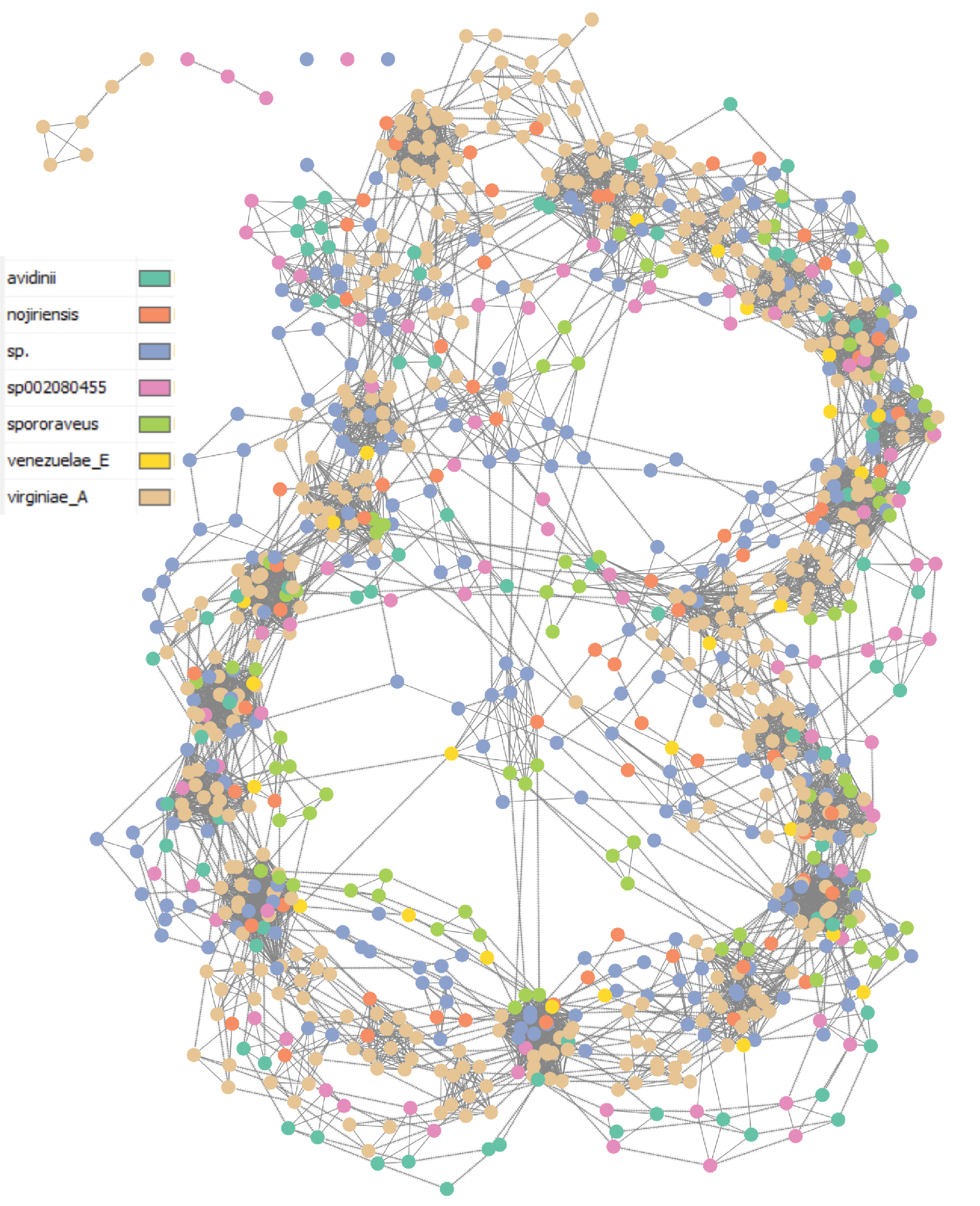


**Fig S20. Similarity network integrated with chromosomal order of BGCs across multiple species of M6_1 Mash-cluster**

Similarity network integrating chromosomal order across 7 different species of Mash-cluster M6_1 depicting the conserved and variable BGCs across the genomes.


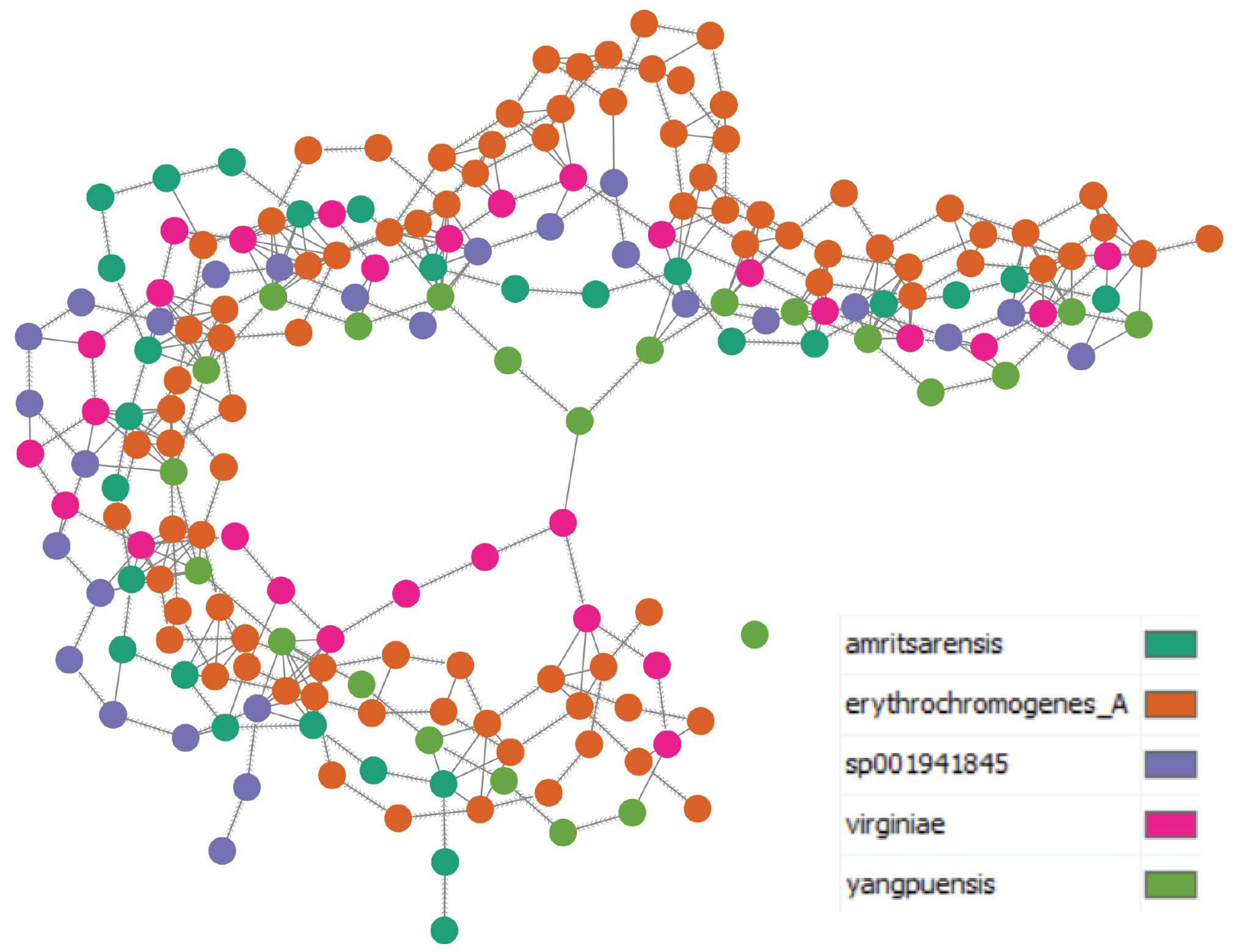


**Fig S21. Similarity network integrated with chromosomal order of BGCs across multiple species of M6_2 Mash-cluster**

Similarity network integrating chromosomal order across 5 different species of Mash-cluster M6_2 depicting the conserved and variable BGCs across the genomes.

**
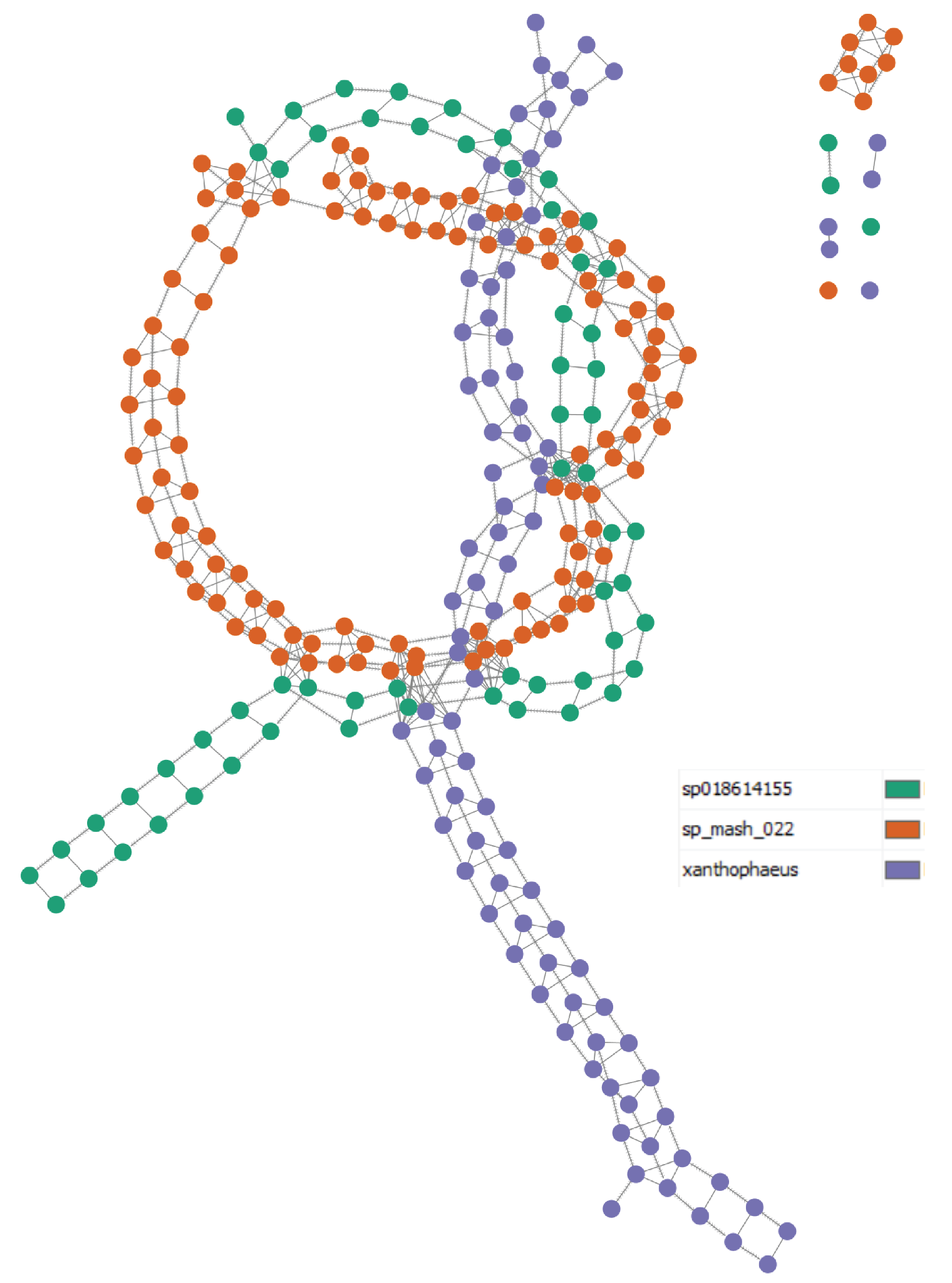
**

**Fig S22. Similarity network integrated with chromosomal order of BGCs across multiple species of M6_3 Mash-cluster**

Similarity network integrating chromosomal order across 3 different species of Mash-cluster M6_3 depicting the conserved and variable BGCs across the genomes. All 3 species of this Mash-cluster are relatively diverse and share less number of common BGCs.


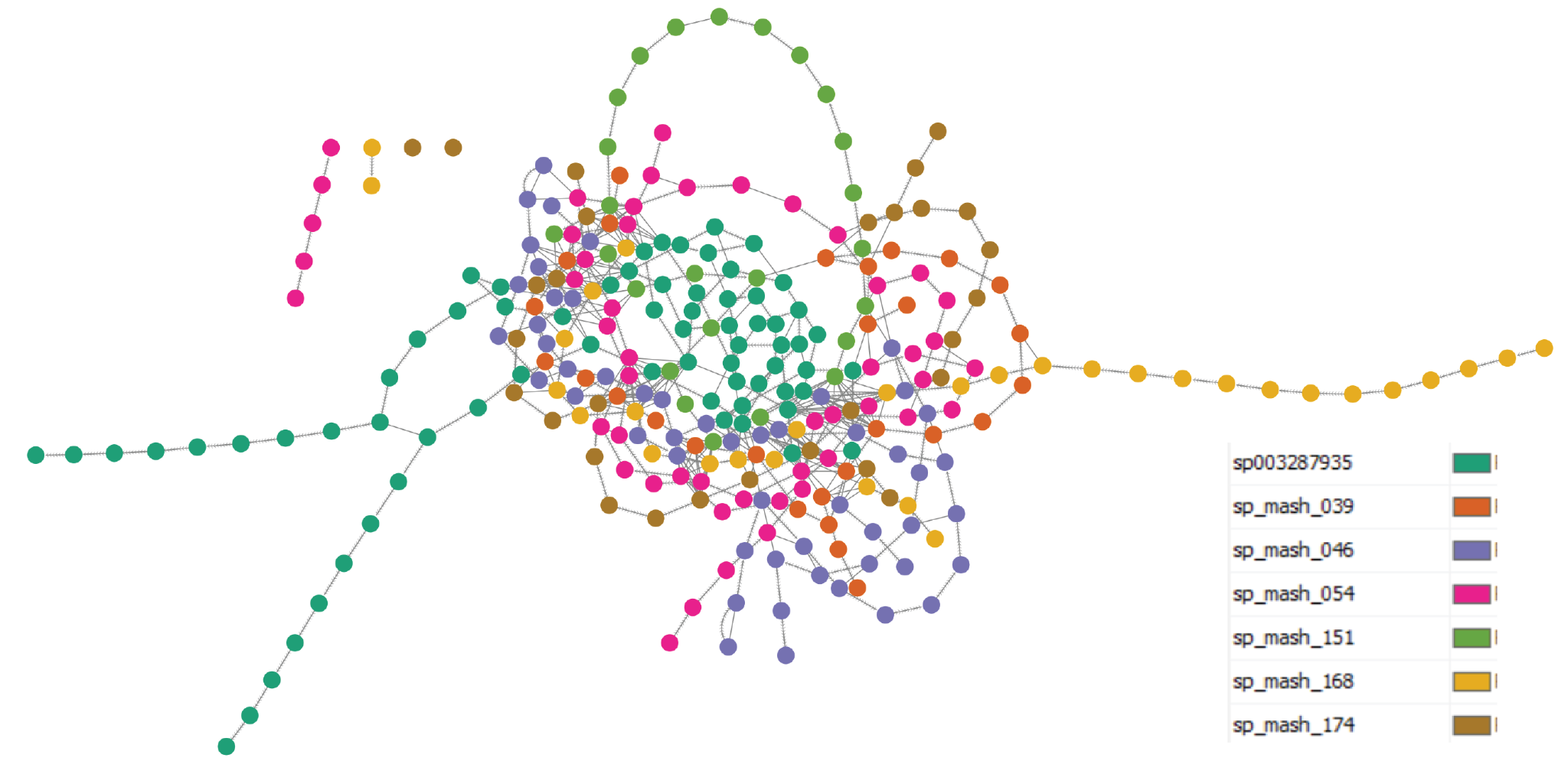


**Fig S23. Similarity network integrated with chromosomal order of BGCs across multiple species of M6_4 Mash-cluster**

Similarity network integrating chromosomal order across 7 different species of Mash-cluster M6_4 depicting the conserved and variable BGCs across the genomes. The species of this Mash-cluster have relatively high diversity of BGCs.

**
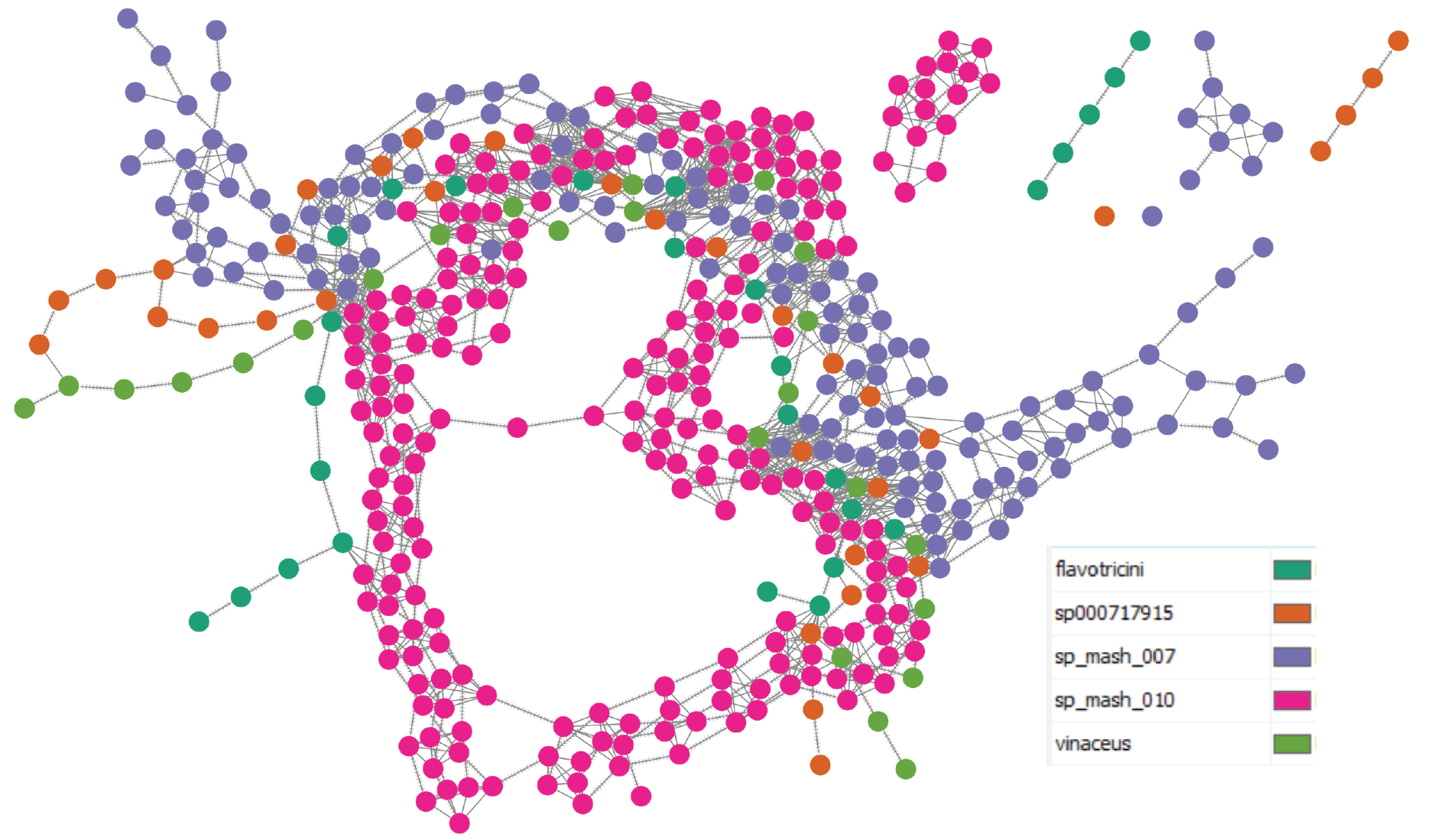
**

**Fig S24. Similarity network integrated with chromosomal order of BGCs across multiple species of M6_5 Mash-cluster**

Similarity network integrating chromosomal order across 5 different species of Mash-cluster M6_5 depicting the conserved and variable BGCs across the genomes. Mash-cluster M6_5 has relatively high diversity of BGCs.

**
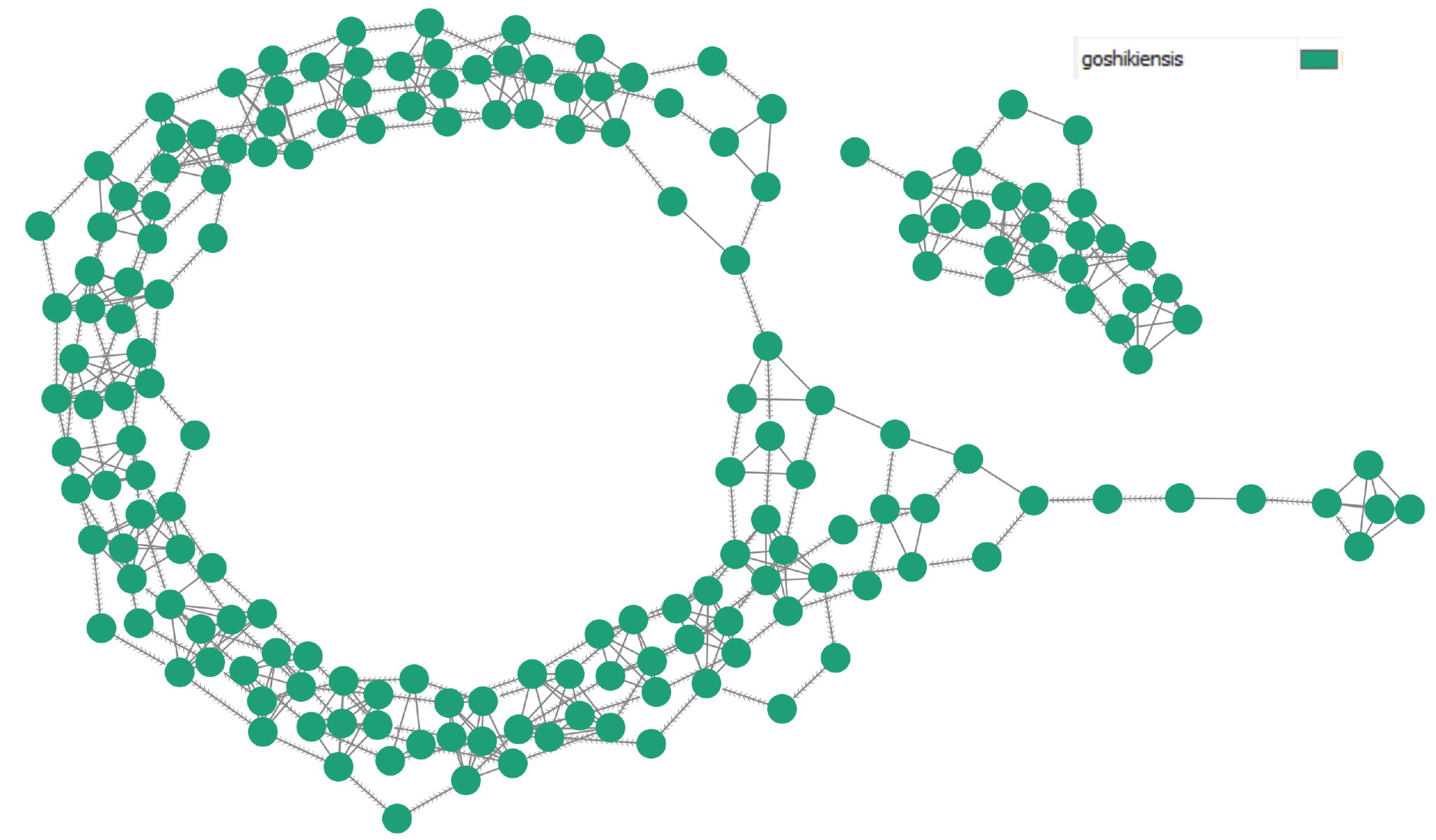
**

**Fig S25. Similarity network integrated with chromosomal order of BGCs across multiple species of M6_6 Mash-cluster**

Similarity network integrating chromosomal order across Mash-cluster M6_6, which consists of only 1 species, depicting the conserved and variable BGCs across the genomes.


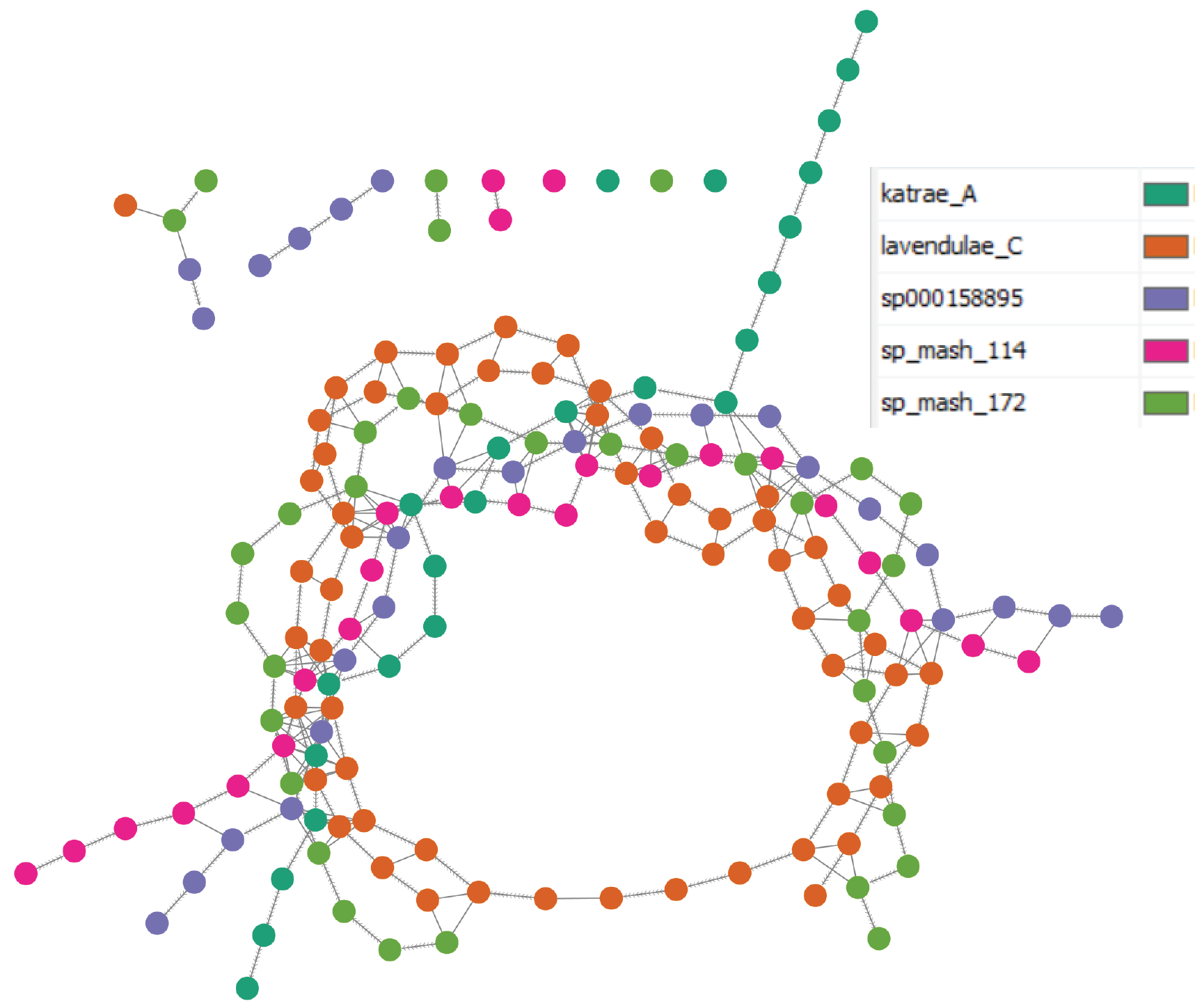


**Fig S26. Similarity network integrated with chromosomal order of BGCs across multiple species of M6_7 Mash-cluster**

Similarity network integrating chromosomal order across 4 different species of Mash-cluster M6_7 depicting the conserved and variable BGCs across the genomes.

**
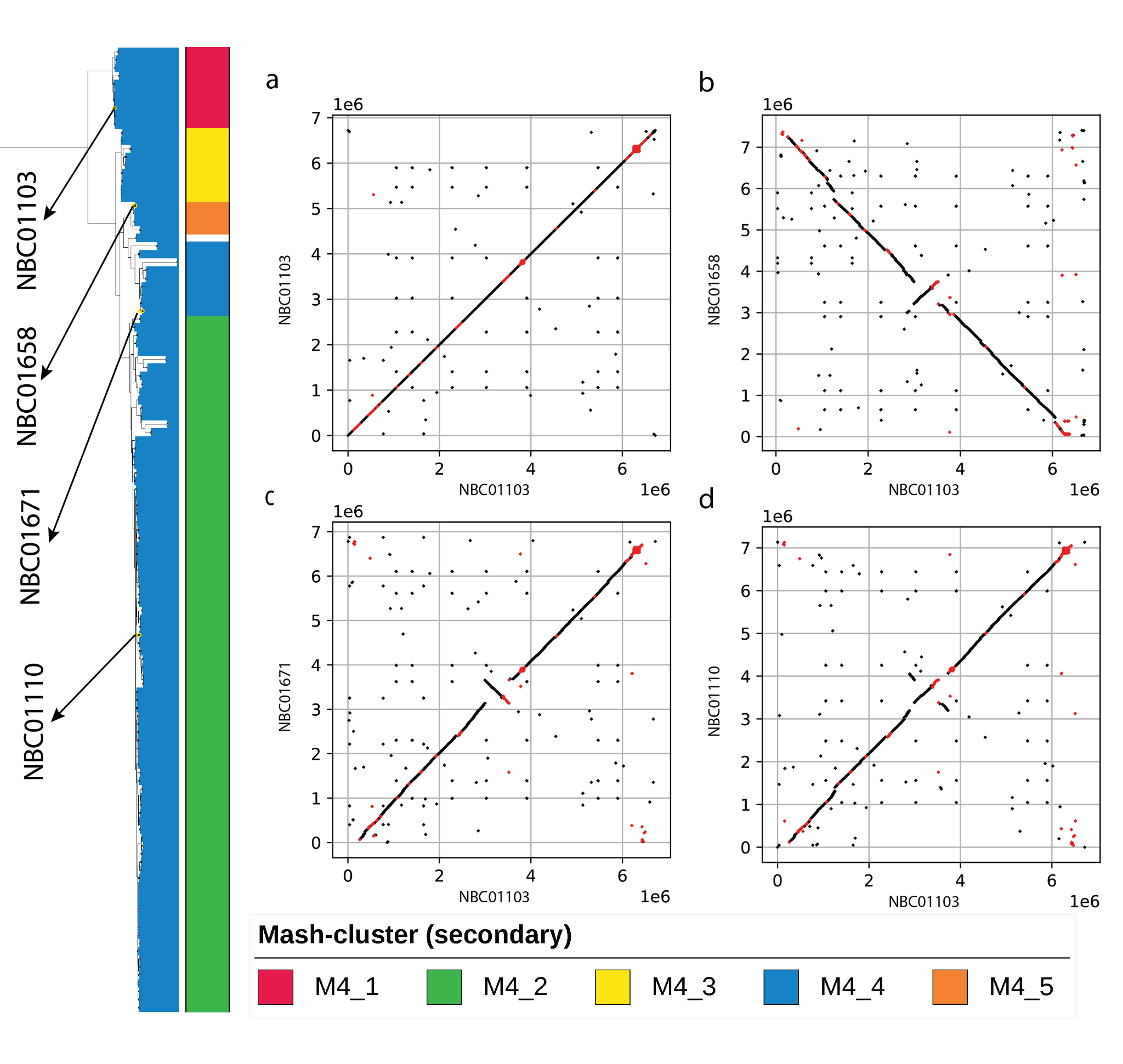
**

**Fig S27. Dotplots showing the conserved chromosomal structure of Mash-cluster M4**

A representative from the earliest diverging subcluster (M4_1) *Streptomyces diastaticus* NBC 01103 is compared to representative strains from subclusters M4_5 (*Streptomyces albidoflavus* NBC 01658) (b), M4_2 (*Streptomyces albidoflavus* NBC 01671) (c), M4_1 (*Streptomyces alboflavus* NBC 01110) (d), and itself (a). Regions corresponding to biosynthetic gene clusters (BGCs) from *Streptomyces diastaticus* NBC 01103 are highlighted in red.


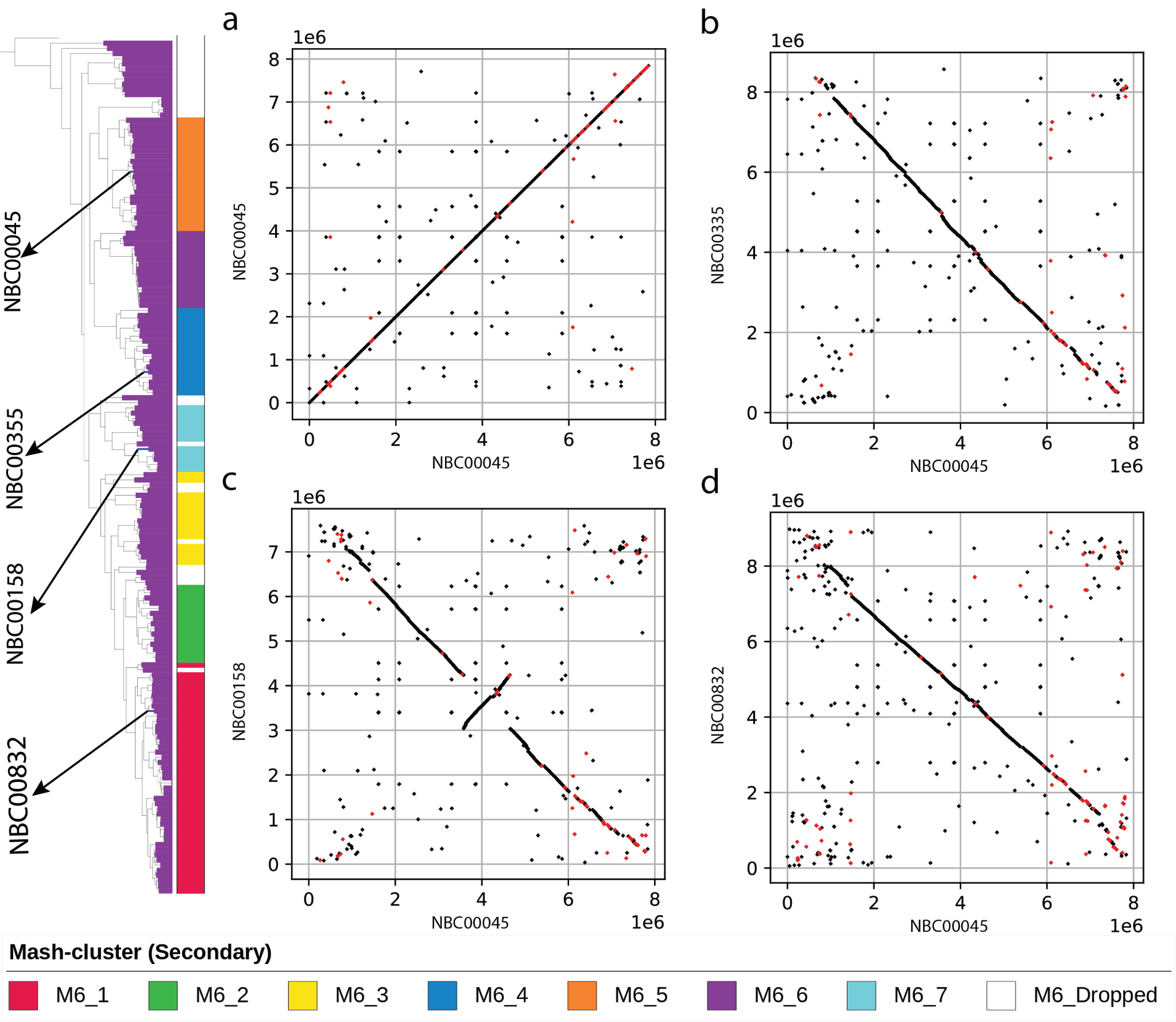


**Fig S28. Dotplots showing the conserved chromosomal structure of Mash-cluster M6**

A representative from one of the three the earliest diverging subgroups (M6_5) *Streptomyces sp*. NBC 00045 is compared to representative strains from subclusters M6_4 (*Streptomyces sp.* NBC 00335) (b), M6_7 (*Streptomyces sp*. NBC 00158) (c), M6_1 (*Streptomyces avidinii* NBC 00832) (d), and itself (a). Regions corresponding to biosynthetic gene clusters (BGCs) from *Streptomyces sp*. NBC 00045 are highlighted in red.

**
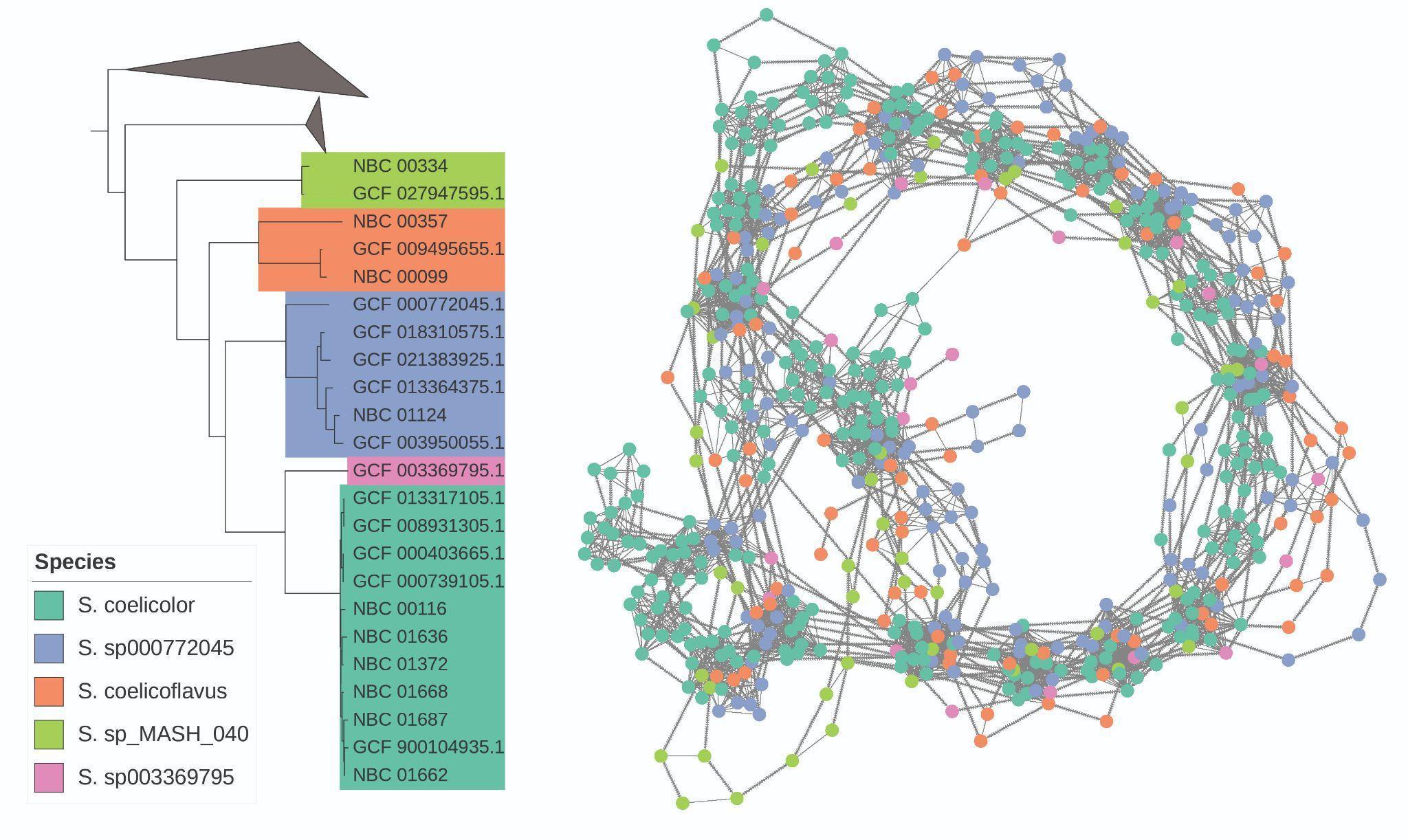
Fig S29. Similarity network integrated with chromosomal order of BGCs across multiple species of M2_3 Mash-cluster**

(*Left*) Phylogenetic tree representing 23 genomes belonging to M2_3 secondary Mash-cluster. The other clades of the Mash-cluster were collapsed. (*Right*) Similarity network integrating chromosomal order across 5 different species of Mash-cluster M2_3 depicting the conserved and variable BGCs across the genomes.
