# Supplementary material for: Pangenome mining of the Streptomyces genus redefines species’ biosynthetic potential
Source: Genome Biol. 2025 Jan 14;26:9. doi: 10.1186/s13059-024-03471-9 (PMC11734326; doi:10.1186/s13059-024-03471-9)
Supplement: Supplementary file 9 — Additional file 9. Review history. The review history for this manuscript. [file 13059_2024_3471_MOESM9_ESM.docx]

**1^st^ round**

**Reviewer 1**

The paper by Mohite and co-workers describes a meta-analysis of the biosynthetic gene clusters in the genus Streptomyces. This genus is highly diverse (more like a family) and historically important as a source of natural products. The authors do an exhaustive analysis and provide considerable new insight into both the biosynthetic potential of this group of bacteria and best practices for how these types of analyses should be performed. Importantly, they provide guidance on how to more accurately predict biosynthetic diversity given that this can be overestimated when using standard bioinformatic tools. For these reasons, I believe this study represents an important contribution. However, there are several issues I believe the authors should consider before the paper is suitable for publication.

My major concern is the lack of specific details describing how some analyses were performed. Enough information should be provided so that the analyses can be repeated, which at present is not the case. For example, the github workflow cited (line 446) appears to suggest that ANI was used to group species? Other examples below.

It's not clear if any further assembly was done on the genomes. If so, how this was done needs to be explained. E.g., if a reference genome was used, that could artificially create synteny, which could account for the conservation of BGC order that was overserved.

Some references are not appropriate for the statements made. E.g., line 56: a mini review is cited instead of the primary literature, line 66: cite the studies where this has been shown not the references for the tools.

Minor points: linen 78: the concept of an open genome should be explained. Mira et al., 2010 International Microbiology or similar would be a good reference.

Line 103: it should be explained how GTDB was used to assign species.

Line 106: This is the first mention of the MASH analysis. An introduction to how this method works (including K-mer selected for the analysis) would be useful. Since the MASH analyses are fundamental to the study, the authors should consider describing the method in the introduction. I'm surprised the authors did not calculate ANI values and compare 95% ANI groups with the MASH results given that this is a common metric used to assess genome level species diversity. I for one would have been interested in seeing how these approaches compared. On line 465, it is stated that in the MASH-based similarity network the edges represent 95% similarity. This level is commonly used for ANI values, but it's not clear to me if this also applies to MASH analyses. Some clarification here would be useful. Line 192: were different parameters used for the second round of clustering?

Line 131: It could also be mentioned that antiSMASH struggles to distinguish among multiple BGCs when they are clustered and this can lead to an underestimation of the total number.

Line 138: These deletions should be clarified. The strains were genetically manipulated before the genomes were deposited?

Line 141: terpene is a compound class not a BGC type.

Line 146: MIBiG estimates the number of BGCs that have experimentally validated products - not those that encode known metabolites. Same comment for line 301. These sentences should be revised.

Line 156: 31% is high and reflects the fact that many characterized BGCs are from streptomycetes. It could be useful for readers if this perspective was added.

Line 176: This paragraph could be better placed in the introduction or possibly discussion as no results are presented.

Line 185: I'm not sure I would call it "statistical". Numeric, metric based?

Line 186: I would consider explaining this method in the introduction. And be sure to provide details in the methods on what K-mer was used for the analysis so it can be repeated.

Line 199: It's not clear why this cluster (M5) was considered ancestral based on Figure 2C.

Line 238: can anything be said about how many Streptomyces genera are predicted from these analyses? Is each primary MASH group considered to be a genus? If so, make this clearer early on?

Line 257: The reduction from 2729 to 440 is quite extraordinary and an important part of this study as it emphasizes that without this extra step the number of GCFs would have been overestimated by a factor of almost 7. I think the authors should emphasize this result and make clear it was only possible because the MIBiG BGCs were available for the analysis. The unknown GCFs (8799) are also likely to be an overestimation since there is no way to make a similar correction. This should be clearly stated.

Line 322: any alternative explanations for the conserved order? I have to say I'm a bit suspicious of this given that most BGCs in Streptomyces genomes, which are linear, are on the chromosome arms where considerable rearrangements are observed. If many of these genomes were assembled using the same reference, that could account for the conservation? Maybe this holds for M4, which is by far the least diverse group and may only be one species? Does it hold for other MASH groups? The authors may want to add some qualifiers to this section - in that it was only shown for one relatively low diversity group and may not apply more broadly applicable when the genus is considered.

Line 412: It is not clear to me how this statement is supported by the results: "This analysis illustrates how neighboring species have evolved distinct strategies to harbor diverse BGCs at specific chromosomal positions."

Conclusions: the observation that GCFs are conserved at various taxonomic levels ("biosynthetic signatures") supports the idea that the products represent ecotype defining traits. The authors may consider adding something to this effect. Especially when the conservation is observed at the species level. This could help support the use of BGCs in defining species boundaries and make the paper of broader interest to biologists.

Lines 457 and 462 and elsewhere: there are a lot of results presented in the methods section. Line 510: remove extraneous text from the methods section.

**Reviewer 2**

The paper by Mohite et al. reports a comprehensive analysis of specialized metabolite gene clusters (BGCs) dynamics toward the Streptomyces genus. This article addresses some very interesting questions about the diversification of BGCs. Combining Mash and BGC analyses seems like a promising way to delve into the history of these gene clusters of biotechnological interest. The authors developed an original workflow to analyze BGC synteny within genome groups. In particular, the study sheds light on the question of whether BGCs are predominantly inherited vertically, or horizontally acquired. Overall, the work is interesting. The manuscript is well-written, and the authors introduce a novel perspective to our understanding of BGC dynamics in Streptomyces. Remarkably, this research encompasses the largest pool of genomes analyzed in this field to date. The analytical methodology is meticulously executed, with the inherent limitations of the approaches thoughtfully addressed at each stage. Below, I've highlighted several remarks and questions that, if addressed by the authors, would strengthen the conclusions of this work about BGC transmission/diversification.

Main comments:
- Would the authors have a way of quantifying the proportion of potentially vertically versus horizontally inherited BGCs in complete genomes to strengthen their conclusion about the main mechanisms of BGC diversification? Including data on the proportion of BGCs per genome that are highly shared with other clusters, those shared with other clusters where BGCs are either common or rare, and cluster-specific BGCs could help approach this question.
- It would be interesting to include the consensus tree from figure S14 in the main figures, as this tree combines information on Mash-clustering, RED groups and an evolutionary model, thus offering a more integrated/robust view. For a thorough vertical versus horizontal transmission analysis, it's advisable to focus on groups where all three methods yield consistent results.
- While the study initially includes a large number of genomes, the conclusion about vertical transmission based on BGC synteny concerns a small number of them: 49 complete genomes for the M4 cluster, 23 genomes for the M2 cluster. Furthermore, the M4 cluster predominantly comprises the same species, S. albidoflavus, in which multiple BGCs had been deleted (line 138 - Fig.1B). Would it be possible to address potential biases or limitations inherent in the analysis of this genome set?
- The BGC synteny analysis conducted on cluster M2 seems to approach also the species level. It raises the question of whether it's unexpected to find such pronounced BGC synteny at this evolutionary distance. Would it be possible to provide insights on this observation?
- The authors indicate that they have observed this remarkable synteny "extended across our entire dataset" (Line 343). Would it be possible to present results obtained from more diversified clusters to strengthen the conclusions regarding the predominantly vertical nature of the transmission, and/or to specify if this conclusion applies only at the species level or close to it?
- A parallel between the synteny of BGCs and that of the core genome (10.1099/mgen.0.000525 ; 10.1093/nar/gkac1076) could be mentioned, as Streptomyces presents a rather remarkable order at both levels. These syntenies may favor homologous recombination and thus gene acquisition by horizontal transfer. Indeed, the presence of a different BGC between two conserved BGCs (as illustrated in Fig 4) could possibly result from horizontal transfer followed by recombination between conserved regions on either side of the new BGC. Could please the authors comment on this point?
- Figures S17 and S18 illustrate several cases of GCF mainly present in one cluster and more rarely in others (e.g. A-factor, scabichelin, tripartilactam). Could these occurrences potentially signify horizontal gene transfers?
- The introduction and discussion seem somewhat brief regarding the debate between vertical and horizontal transmission as the primary factor of BGC diversification. Only one reference is cited in introduction. The discussion refers mainly to articles that bring evidence of vertical transmission. It would be beneficial to provide a comprehensive understanding of the debate by presenting also the studies that have led to the proposal of BGC dynamics also based on horizontal transmission, and how/why the present data do not capture so much this information.
- The observation that the extremities of the Streptomyces genome harbor more variable, often strain-specific, regions compared to the central part of the genome has been documented since the earliest sequenced genomes in Streptomyces. Given the data presented by the authors on genomes with complete assemblies (HQ), is there discernible evidence of this distinction reflected in the evolution of BGCs? Recent advancements in 3D studies of Streptomyces linear genomes, coupled with bioinformatics analyses, have revealed that the central compartment can be delineated by the distal ribosomal RNA encoding operons (10.1093/nar/gkac1076). Would it be possible to consider whether there are disparities in the rate of evolution and/or vertical transmission and/or type of GCF carried by the central and terminal compartments? Please note that S. albidoflavus exhibits some of the smallest terminal compartments, which is consistent with its smaller genome size and relatively low number of BGCs (as illustrated in Figure 1.B). Consequently, groups other than M4 might offer better insights into exploring this question.
- Plasmids can also be a source of BGCs (https://journals.asm.org/doi/10.1128/spectrum.01523-23). Based on the data at their disposal, could the authors provide some information about BGCs and GCFs present on plasmids?

Minor points:
- Previous studies have also documented information regarding the most common BGCs and the count of BGCs per strain. Could the authors cite these studies and conduct a comparative analysis of their findings. For instance, Belknap et al. (10.1038/s41598-020-58904-9) noted a median of 40 BGCs per strain. This variance might originate from sampling biases, such as potential over-representation of certain species. Would it be possible to calculate also the number of BGCs per genome considering only distinct species (selected based on Mash clustering) to provide a genus-wide overview of distribution, minimizing bias toward over-representation of certain species?
- Would it be possible to specify the placement of clades I, II, and the O group previously described by McDonald and Currie (10.1128/mbio.00644-17) within the consensus tree from Figure S14? Does this phylogenetic analysis align with the core genome phylogenetic tree published in (https://doi.org/10.3389/fmicb.2021.677558; or 10.1093/nar/gkac1076)?
- Abstract - lines 39-40: Traditionally, the phrase 'acquisition of new genes' tends to evoke the idea of horizontal gene transfer rather than vertical inheritance. To better convey the concept of vertical inheritance, it might be more fitting to discuss 'BGC diversification'.
- The term 'Mash' (first letter in uppercase followed by lowercase) should be used instead of 'MASH', in accordance with the nomenclature originally published in Genome Biology (DOI 10.1186/s13059-016-0997-x). This will also prevent any confusion with antiSMASH, which readers might naturally associate with BGC clusters.
- Figure 1: Would it be possible to revise the panel C to enhance the coherence of the classification? At first glance, some categories may seem to overlap or appear disparate, such 'NRPS' and 'NRPS-like'. I understand that the current classification follows traditional pipeline biosynthetic (sub)classes, but is there a method to extract the logic more effectively? For instance, grouping all NRPS and NRPS-like categories together or arranging single modules first, followed by double modules, then hybrids could enhance clarity. The current descending order of abundance of sub-categories makes it challenging to discern a clear message regarding biosynthetic classes (NRPS and related categories represented at least three points apart, as with PKS). Additionally, the sentence "Number of known hits against MIBIG vary depending on BGC type" should be removed from the panel, as it is more suited for inclusion in the main text rather than within the figure itself. Please include a list of abbreviations in the legend of this figure.
- Line 76: The list of comparative genomics studies in Streptomyces is not exhaustive (only 2 references cited). For instance, Lorenzi et al. (https://doi.org/10.1099/mgen.0.000525) reported around one thousand core genes. Studies from Nikolaidis et al. (https://doi.org/10.1099/mgen.0.001028) or Belknap et al. (https://www.nature.com/articles/s41598-020-58904-9) may also be interesting to considered.
- Family and genus names should be written in italics (https://doi.org/10.1099/ijsem.0.005585). Please write 'Streptomyces' or 'Streptomycetaceae' in italics at lines: 58, 93, 439, 440, etc. (and in the references).
- Line 203: "(previously designated" should not be written in italics.
- Lines 237 and 501: Perhaps the "_" isn't necessary.
- References need to be edited: references 4, 15 and 38 correspond to the same article (and/or its correction); likewise for 12 and 18.
- Data S1 legend: please replace 'selcted' by 'selected'.
- Figure S2 legend: please replace 'recnet' by 'recent'.
- Figure S14: the word 'color' is present twice.

**Authors’ response**

*We have focused particular efforts on the issues you have highlighted as follows:*

1. *Regarding the ‘suspicions about the conserved order’, we have clarified that the dataset of genomes used in the study was not re-assembled and was directly used as is from the NCBI database. Therefore, these results cannot be an artifact from assembly using a reference genome. The majority of high-quality genomes were de novo assembled in our lab as described in our recent study Jørgensen et al published in NAR (*[*https://doi.org/10.1093/nar/gkae523*](https://doi.org/10.1093/nar/gkae523)*). We have extended the analysis to different Mash-clusters to shed light on the observed pattern across different clades and made points on how these patterns vary along with their limitations. Furthermore, we have provided pairwise dotplots to help illustrate our claims in a more tangible way.*
2. *Regarding* Referee 1’s request to broaden the discussions about the pipeline for finding BGCs, *we have expanded the text to provide details on the pipeline to find BGCs and Mash-clusters. We have also made improvements to the available code to make it easier for other researchers to utilize our pipeline at :* [*https://github.com/NBChub/Streptomyces_pangenome_ms*](https://github.com/NBChub/Streptomyces_pangenome_ms)*.*
3. *Regarding, ‘* the implications this work has for species boundaries’ we want to make it clear that we are not making claims about the taxonomy of these strains. Mash-clusters provide an objective measure for dividing strains into groups that does not rely on species definitions.
4. *We do not make any claims on redefining the taxonomic units such as species,genera or sub-genera. We have modified the text to highlight this fact.*
5. *Finally, regarding ‘* the difference between BGCs at chromosome termini compared to the central portion of the chromosomes’ we have included some additional analysis to*t compare the BGC positions of genomes from different Mash-clusters from M4 and M6 clusters. We also note that we had carried out extensive analysis on the chromosomal locations of different BGC types in our recent study Jørgensen et al (*[*https://doi.org/10.1093/nar/gkae523*](https://doi.org/10.1093/nar/gkae523)*).*

*We hope that the revised manuscript will satisfy the expectations of the editors and the reviewers and look forward to your responses.*

Reviewer #1:

**The paper by Mohite and co-workers describes a meta-analysis of the biosynthetic gene clusters in the genus Streptomyces. This genus is highly diverse (more like a family) and historically important as a source of natural products. The authors do an exhaustive analysis and provide considerable new insight into both the biosynthetic potential of this group of bacteria and best practices for how these types of analyses should be performed. Importantly, they provide guidance on how to more accurately predict biosynthetic diversity given that this can be overestimated when using standard bioinformatic tools. For these reasons, I believe this study represents an important contribution. However, there are several issues I believe the authors should consider before the paper is suitable for publication.**

*>> We thank the reviewer for appreciating the quality of the analysis and the importance of the key findings presented in our study. We welcome the reviewer’s suggestions and believe that these have helped us improve our manuscript further. Please find our responses to each of the raised issues below.*

**My major concern is the lack of specific details describing how some analyses were performed. Enough information should be provided so that the analyses can be repeated, which at present is not the case. For example, the github workflow cited (line 446) appears to suggest that ANI was used to group species? Other examples below.**

*>> We agree that our first submission lacked certain details and thank the reviewer for bringing this important point. We have now improved the details in the revised submission.*

*The BGCFlow workflow (*[*https://github.com/NBChub/bgcflow*](https://github.com/NBChub/bgcflow)*) was used to generate most of the data and tables. These tables were further processed with specific downstream analyses specific to this study - the scripts for which are on GitHub (*[*https://github.com/NBChub/Streptomyces_pangenome_ms*](https://github.com/NBChub/Streptomyces_pangenome_ms)*).*

*BGCFlow workflow is now published* [*[1]*](https://paperpile.com/c/wrXCal/tLKd) *and we have expanded the documentation significantly. The present GitHub workflow also provides tutorials on running large-scale pangenome mining analysis. The workflow allows both fastANI and Mash tools to generate the similarity matrix between all vs all genome comparisons. In this study, we selected Mash as this is a faster tool and provides similar results to ANI as demonstrated by the correlations in the Mash study* [*[2]*](https://paperpile.com/c/wrXCal/df7I) *and another comparative study on different tools* [*[3]*](https://paperpile.com/c/wrXCal/EjeW)*.*

*Furthermore, we have expanded all sections to explain the specific details of the downstream analyses, including species detection (Lines 119-136).*

**It's not clear if any further assembly was done on the genomes. If so, how this was done needs to be explained. E.g., if a reference genome was used, that could artificially create synteny, which could account for the conservation of BGC order that was overserved.**

*>> We did not perform any further assembly of the genomes by using reference genomes. All the genomes studied here are directly downloaded from the NCBI RefSeq database. The RefSeq database also contained 902 high-quality de-novo assembled genomes from our prior study* [*[4]*](https://paperpile.com/c/wrXCal/rzd4)*. These de-novo assembled genomes also form the majority of the dataset and thus our methods do not create artificial synteny impact.*

*We added a sentence in the results section clarifying this at line 109: “We note that all of these 902 newly sequenced genomes were assembled de-novo rather than based on a reference genome. These 902 sequences have also been deposited at RefSeq.”*

**Some references are not appropriate for the statements made. E.g., line 56: a mini review is cited instead of the primary literature, line 66: cite the studies where this has been shown not the references for the tools.**

*>> We have now updated the references to indicate the primary literature in both statements pointed out by the reviewer.*

*Line 55: Added references of Omura et al 2001* [*[5]*](https://paperpile.com/c/wrXCal/xVCT)*, Bently et al 2002* [*[6]*](https://paperpile.com/c/wrXCal/hFMV)*, and Belknap et al 2022* [*[7]*](https://paperpile.com/c/wrXCal/Oths) *for the statement: “Over the past decades, several genomic studies have revealed that the full range of metabolites produced by Streptomyces and the associated biosynthetic pathways are not yet fully known* [*[5–7]*](https://paperpile.com/c/wrXCal/Oths+xVCT+hFMV)*.”*

*Line 65: We updated the statement to make it clear that first statement was about tools and second about the example: “Several genome mining tools such as antiSMASH, BiG-SCAPE, and BiG-SLICE are available to assess the biosynthetic potential encoded in the genomes* [*[8–11]*](https://paperpile.com/c/wrXCal/fuHV+fIxI+p2Dj+S8Z9)*. The genome mining tools have revealed that various bacterial species encode previously unknown biosynthetic potential* [*[12–14]*](https://paperpile.com/c/wrXCal/kz2g+x2jP+Hdit)*.”*

**Minor points: line 78: the concept of an open genome should be explained. Mira et al., 2010 International Microbiology or similar would be a good reference.**

*>> We have now added a sentence describing open pangenome and the suggested reference.*

*Line 79:* *“(a pangenome is said to be open if newly sequenced genomes appear to keep adding novel genes* [*[15]*](https://paperpile.com/c/wrXCal/L8rx)*)”*

**Line 103: it should be explained how GTDB was used to assign species.**

*>> We have expanded the description of species assignment using the GTDB Toolkit and the reference GTDB database. These descriptions were originally included in the Methods section. The genomes without species assignment represent novel species. For the novel species, we used a 95% WGS similarity metric to cluster them into different species equivalents using the similarity network.*

*See lines 119 to 136 for the updated explanations*.

**Line 106: This is the first mention of the MASH analysis. An introduction to how this method works (including K-mer selected for the analysis) would be useful. Since the MASH analyses are fundamental to the study, the authors should consider describing the method in the introduction. I'm surprised the authors did not calculate ANI values and compare 95% ANI groups with the MASH results given that this is a common metric used to assess genome level species diversity. I for one would have been interested in seeing how these approaches compared. On line 465, it is stated that in the MASH-based similarity network the edges represent 95% similarity. This level is commonly used for ANI values, but it's not clear to me if this also applies to MASH analyses. Some clarification here would be useful. Line 192: were different parameters used for the second round of clustering?**

*>> We have now introduced the importance of Mash-clusters in the introduction paragraph (lines 86-94). We have cited other recent studies that have demonstrated the benefits of using genome similarity to groups of species and genera* [*[16,17]*](https://paperpile.com/c/wrXCal/BKCm+RImU)*.*

*The similarity scores of Mash and ANI generally correlate well according to large-scale data analysis carried out in the Mash article* [*[2]*](https://paperpile.com/c/wrXCal/df7I)*. Another study showed that the ANI and Mash perform equally to differentiate between species* [*[3]*](https://paperpile.com/c/wrXCal/EjeW)*. We chose Mash for this study as this method is much faster than calculating ANI.*

*The Mash distance of 0.05 between 2 genomes correlates with 95% ANI distance. Thus we used a similar cutoff of 95% in the similarity network created originally using Mash distance cutoff of 0.05.*

*We used the same parameters for the second round of clustering. The only difference for the second round of clustering is the starting dataset, which only consists of the primary Mash-cluster genomes.*

**Line 131: It could also be mentioned that antiSMASH struggles to distinguish among multiple BGCs when they are clustered and this can lead to an underestimation of the total number.**

*>> As recommended by the reviewer, we have inserted the statement at line 163: “It is essential to highlight that antiSMASH will report two adjacent unrelated BGCs as a single hit.“ to reflect on this issue.*

**Line 138: These deletions should be clarified. The strains were genetically manipulated before the genomes were deposited?**

*>> We changed the sentence at line 174 to: “It should be noted that the set of HQ assemblies included several* ***engineered*** *S. albidoflavus strains in which multiple BGCs had been deleted* ***(e.g.,*** [*[18]*](https://paperpile.com/c/wrXCal/FLa2A)***)****” and added a citation to make this clearer.*

**Line 141: terpene is a compound class not a BGC type.**

*>> This is true however, this is the terminology deployed by antiSMASH. For the purpose of consistency, we have chosen to use the types as defined by the antiSMASH and have clarified that this is an annotation at line 184.*

**Line 146: MIBiG estimates the number of BGCs that have experimentally validated products - not those that encode known metabolites. Same comment for line 301. These sentences should be revised.**

*>> We modified the paragraphs to: (1): “One can estimate the number of BGCs that are experimentally linked with encoded known secondary metabolites by comparing the BGCs against the curated MIBiG database” (line 189), and (2): “This encompassed 289 GCFs with* ***experimentally linked compounds from the MIBiG database*** *and 1,457 putatively novel GCFs. We found that 48 of the* ***“****known****”*** *GCFs (such as ectoine, hopene, desferrioxamine, etc.) displayed a widespread genomic distribution, being present in genomes across all MASH-clusters” (line 358)*

**Line 156: 31% is high and reflects the fact that many characterized BGCs are from streptomycetes. It could be useful for readers if this perspective was added.**

*>> As recommended, we added: “These high numbers also reflect the fact, that 41.6% of the MIBiG entries originate from Actinobacteria.” at line 202.*

**Line 176: This paragraph could be better placed in the introduction or possibly discussion as no results are presented.**

*>> As recommended we have moved this paragraph to the discussion section at lines 465 to 474.*

**Line 185: I'm not sure I would call it "statistical". Numeric, metric based?**

*>> We have replaced “statistical” with “numerical” at line 230.*

**Line 186: I would consider explaining this method in the introduction. And be sure to provide details in the methods on what K-mer was used for the analysis so it can be repeated.**

*>> We have now briefly introduced the Mash-based clustering in the introduction (lines 86-94) and also expanded the details in the results section (lines 230-234) and methods section (line 599). Default K-mer value of 21 was used to run Mash.*

**Line 199: It's not clear why this cluster (M5) was considered ancestral based on Figure 2C.**

*>> M5 is described as ancestral as it is the first Mash-cluster to diverge. We agree, however, that this terminology may be confusing. This line is now removed at line 249.*

**Line 238: can anything be said about how many Streptomyces genera are predicted from these analyses? Is each primary MASH group considered to be a genus? If so, make this clearer early on?**

*>> We are a little unclear about what this comment is referring to as Streptomyces is the genus.*

*Additionally, we do not want to comment on the taxonomy of the genus as this is a highly contentious issue. An advantage of the Mash-clusters is that they can allow for analysis independent of taxonomic assignments. In the manuscript, we have chosen to use GTDB’s definition of the taxonomic levels as they are reproducible and can be assigned in silico based on the genome data. We have addressed this issue in the Discussions at lines 465 to 474.*

**Line 257: The reduction from 2729 to 440 is quite extraordinary and an important part of this study as it emphasizes that without this extra step the number of GCFs would have been overestimated by a factor of almost 7. I think the authors should emphasize this result and make clear it was only possible because the MIBiG BGCs were available for the analysis. The unknown GCFs (8799) are also likely to be an overestimation since there is no way to make a similar correction. This should be clearly stated.**

*>> We have now added a paragraph at lines 332-335 on these unknown GCFs stating: “The regrouping of 2,729 known GCFs was carried out on the basis of the similarity to nearest MIBiG BGCs. For the remaining 8,799 unknown GCFs, this analysis was not possible at this stage. This diversity of unknown GCFs is also likely an overestimation which can’t yet be tested by our approach.”*

**Line 322: any alternative explanations for the conserved order? I have to say I'm a bit suspicious of this given that most BGCs in Streptomyces genomes, which are linear, are on the chromosome arms where considerable rearrangements are observed. If many of these genomes were assembled using the same reference, that could account for the conservation? Maybe this holds for M4, which is by far the least diverse group and may only be one species? Does it hold for other MASH groups? The authors may want to add some qualifiers to this section - in that it was only shown for one relatively low diversity group and may not apply more broadly applicable when the genus is considered.**

*>> We did not perform any further assembly of the genomes by using reference genomes. All the genomes studied here are directly downloaded from the NCBI RefSeq database. The RefSeq database also contained 902 high-quality de-novo assembled genomes from our prior study* [*[4]*](https://paperpile.com/c/wrXCal/rzd4)*. These de-novo assembled genomes also form the majority of the dataset and thus our methods do not create artificial synteny impact.*

*We have now added results on the BGC similarity networks from other Mash-clusters from M6, which showed that this method also holds true beyond the M4 Mash-cluster. We also note that the pattern is more evident in Mash-clusters that have high silhouette scores representing high confidence clustering. See lines 419 to 441, Figure 5, Figures S19-S27.*

*We have further discussed this in the lines 521-526.*

*We have also added dotplots in Figures S28 and S29 for a selected pairs from different Mash-clusters.*

**Line 412: It is not clear to me how this statement is supported by the results: "This analysis illustrates how neighboring species have evolved distinct strategies to harbor diverse BGCs at specific chromosomal positions."**

*>> We have now updated this statement to a more general observation and possible method to investigate the evolution of BGCs by means of identifying the variable BGC regions. We believe that the observation of this study can lead researchers to investigate BGC diversity in the context of chromosomal locations in addition to similarity networks.*

*See lines 521-526: “Over and over again the similarity networks of closely related genomes from species or Mash-clusters showed conserved order of common BGCs and variable BGCs in the neighborhood between conserved BGCs. Such representation could help show how neighboring species or closely related genomes have evolved to harbor diverse BGCs at specific chromosomal positions.”*

**Conclusions: the observation that GCFs are conserved at various taxonomic levels ("biosynthetic signatures") supports the idea that the products represent ecotype defining traits. The authors may consider adding something to this effect. Especially when the conservation is observed at the species level. This could help support the use of BGCs in defining species boundaries and make the paper of broader interest to biologists.**

*>> We have now added a statement in the conclusion at line 552 - “*Our results on GCF conservation at taxonomic levels support that the BGCs are key traits of species defining their ecotypes.*”*

**Lines 457 and 462 and elsewhere: there are a lot of results presented in the methods section.**

*>> We have removed these sentences from the Methods section and moved them to the appropriate places in the Results section.*

**Line 510: remove extraneous text from the methods section.**

*>> We have now removed extraneous text from the methods section.*

**Reviewer #2:**

**The paper by Mohite et al. reports a comprehensive analysis of specialized metabolite gene clusters (BGCs) dynamics toward the Streptomyces genus. This article addresses some very interesting questions about the diversification of BGCs. Combining Mash and BGC analyses seems like a promising way to delve into the history of these gene clusters of biotechnological interest. The authors developed an original workflow to analyze BGC synteny within genome groups. In particular, the study sheds light on the question of whether BGCs are predominantly inherited vertically, or horizontally acquired. Overall, the work is interesting. The manuscript is well-written, and the authors introduce a novel perspective to our understanding of BGC dynamics in Streptomyces. Remarkably, this research encompasses the largest pool of genomes analyzed in this field to date. The analytical methodology is meticulously executed, with the inherent limitations of the approaches thoughtfully addressed at each stage. Below, I've highlighted several remarks and questions that, if addressed by the authors, would strengthen the conclusions of this work about BGC transmission/diversification.**

**Main comments:**

**- Would the authors have a way of quantifying the proportion of potentially vertically versus horizontally inherited BGCs in complete genomes to strengthen their conclusion about the main mechanisms of BGC diversification? Including data on the proportion of BGCs per genome that are highly shared with other clusters, those shared with other clusters where BGCs are either common or rare, and cluster-specific BGCs could help approach this question.**

*>> We appreciate the importance of quantifying vertically vs horizontally inherited BGCs. However, we think that making such quantitative claims about horizontal transfer would require significant additional analysis beyond the scope of this study. Here, we wanted to focus on the pangenome analysis of the Streptomyces by grouping the genomes into different Mash-clusters that would allow investigation of BGCs present across them. We appreciate that the raised question would be of high interest and we hope that this study will lead to the availability of the data to design future studies solely focused on the evolution of BGCs.*

**- It would be interesting to include the consensus tree from figure S14 in the main figures, as this tree combines information on Mash-clustering, RED groups and an evolutionary model, thus offering a more integrated/robust view. For a thorough vertical versus horizontal transmission analysis, it's advisable to focus on groups where all three methods yield consistent results.**

*>> We understand that including the tree from S14 in the Main figures could be valuable in a study focused on the evolution of the BGCs. We are hesitant to include the tree as a replacement for Figure 2B as most of the branches are not resolved in the consensus tree and would potentially lead to more confusion. We believe that a separate dedicated study would be needed for resolving these tree branches and then studying the evolutionary history of specific BGCs.*

**- While the study initially includes a large number of genomes, the conclusion about vertical transmission based on BGC synteny concerns a small number of them: 49 complete genomes for the M4 cluster, 23 genomes for the M2 cluster. Furthermore, the M4 cluster predominantly comprises the same species, S. albidoflavus, in which multiple BGCs had been deleted (line 138 - Fig.1B). Would it be possible to address potential biases or limitations inherent in the analysis of this genome set?**

*>> We have now expanded the analysis by including a much more diverse Mash-cluster M6 in addition to examples of M4 and M2. A new paragraph was added at lines 419 to 441 including a main Figure 5, and supplementary figures S19 to S29. Overall, we observe that the pattern of conserved order of BGCs that are common and then the variable BGC regions holds for other genomes from species level and for the secondary Mash-clusters that have high confidence of clustering based on silhouette scores. We have added statements to make this more clear that the analysis depends on the selection of genomes from neighbouring species or from consistent Mash-clusters.*

**- The BGC synteny analysis conducted on cluster M2 seems to approach also the species level. It raises the question of whether it's unexpected to find such pronounced BGC synteny at this evolutionary distance. Would it be possible to provide insights on this observation?**

*>> We agree that this result is surprising (also see response to reviewer 1).*

*See lines 426-435 for different examples of Mash-clusters displaying slightly different patterns based on the silhouette clustering scores of the Mash-clusters. However, we still observe a general pattern across the entire Mash-cluster M6 which is highly diverse compared to M2.*

*We have also added statements to provide more insights on our observations and the importance of our strategy in lines at line 439: “Thus, the strategy to integrate BGC similarity and synteny provides an effective way to select different levels of Mash-clusters and investigate them individually.”*

**- The authors indicate that they have observed this remarkable synteny "extended across our entire dataset" (Line 343). Would it be possible to present results obtained from more diversified clusters to strengthen the conclusions regarding the predominantly vertical nature of the transmission, and/or to specify if this conclusion applies only at the species level or close to it?**

*>> In response to this specific comment, we’ve expanded the analysis to another Mash-cluster, M6, as an additional example. We have further added a statement in the Discussion highlighting the neighboring species or more closely related genomes could be investigated using the presented analysis in line 524: “Such representation could help to show how neighboring species or closely related genomes have evolved to harbor diverse BGCs at specific chromosomal positions.”*

*These observations are discussed in two paragraphs on lines from 510 to 538.*

**- A parallel between the synteny of BGCs and that of the core genome (10.1099/mgen.0.000525 ; 10.1093/nar/gkac1076) could be mentioned, as Streptomyces presents a rather remarkable order at both levels. These syntenies may favor homologous recombination and thus gene acquisition by horizontal transfer. Indeed, the presence of a different BGC between two conserved BGCs (as illustrated in Fig 4) could possibly result from horizontal transfer followed by recombination between conserved regions on either side of the new BGC. Could please the authors comment on this point?**

*>> It is certainly the case that synteny can be informative of potential HGT. However, as mentioned above we do not have the capacity to analyze this across our whole dataset as predicting BGC boundaries is incredibly difficult/impossible. It is worth mentioning that the distance between neighboring BGCs can sometimes be very large (up to 100s Kbs) so it is difficult to comment on this. The reviewer may well be correct, but we are not comfortable commenting on this in the manuscript.*

**- Figures S17 and S18 illustrate several cases of GCF mainly present in one cluster and more rarely in others (e.g. A-factor, scabichelin, tripartilactam). Could these occurrences potentially signify horizontal gene transfers?**

*>> Again, this could be correct but quantifying if these GCFs are horizontally transferred would require significant additional investigation, which in our opinion is out of the scope of this study. Other explanations are that they are present in the ancestor and have been lost.*

**- The introduction and discussion seem somewhat brief regarding the debate between vertical and horizontal transmission as the primary factor of BGC diversification. Only one reference is cited in introduction. The discussion refers mainly to articles that bring evidence of vertical transmission. It would be beneficial to provide a comprehensive understanding of the debate by presenting also the studies that have led to the proposal of BGC dynamics also based on horizontal transmission, and how/why the present data do not capture so much this information.**

*>> We have now expanded the Discussion section to include the discussion on vertical vs horiztonatal transmission of BGCs at the lines 528 to 538.*

**- The observation that the extremities of the Streptomyces genome harbor more variable, often strain-specific, regions compared to the central part of the genome has been documented since the earliest sequenced genomes in Streptomyces. Given the data presented by the authors on genomes with complete assemblies (HQ), is there discernible evidence of this distinction reflected in the evolution of BGCs? Recent advancements in 3D studies of Streptomyces linear genomes, coupled with bioinformatics analyses, have revealed that the central compartment can be delineated by the distal ribosomal RNA encoding operons (10.1093/nar/gkac1076). Would it be possible to consider whether there are disparities in the rate of evolution and/or vertical transmission and/or type of GCF carried by the central and terminal compartments? Please note that S. albidoflavus exhibits some of the smallest terminal compartments, which is consistent with its smaller genome size and relatively low number of BGCs (as illustrated in Figure 1.B). Consequently, groups other than M4 might offer better insights into exploring this question.**

*>>Again, investigating evolutionary rates at this scale would take significant additional analysis. With regards to* *S. albidoflavus we have included additional figures (S28-S29) that confirm the small size of the termini. As the reviewer suggested, we have also added additional analysis on group M6 (Figure 5, Figures S20-S27), which provides an interesting contrast. Chiefly that the terminal regions are much less conserved.*

**- Plasmids can also be a source of BGCs (https://urldefense.com/v3/__https://journals.asm.org/doi/10.1128/spectrum.01523-23__;!!Mih3wA!E1XY1WDOJi_nHKsUIbda1h9pLfigvbRr-O_XI2P1HWpC2I17JaxBofMwm0M8FsAOA-x-jmngII2jV9sD$ ). Based on the data at their disposal, could the authors provide some information about BGCs and GCFs present on plasmids?**

*>> We have added these stats at lines 180-183:* “Plasmids can also be source of BGCs [[19]](https://paperpile.com/c/wrXCal/5Z8w), however there are limitations on classification of plasmids, circularity, highly diverse plasmid sized in *Streptomyces*. Of the 1,215 HQ genomes, around 352 genomes had BGCs on plasmids with median of 2 BGCs sourced from plasmids among them. For this study, plasmid- and Chromosome-encoded BGCs were analyzed together.”

*We note that for several of the publicly available genomes, the plasmids may not even been sequenced and made available. It would be difficult for us to give accurate statistics on the BGCs and GCFs present in plasmids.*

**Minor points:**

**- Previous studies have also documented information regarding the most common BGCs and the count of BGCs per strain. Could the authors cite these studies and conduct a comparative analysis of their findings. For instance, Belknap et al. (10.1038/s41598-020-58904-9) noted a median of 40 BGCs per strain. This variance might originate from sampling biases, such as potential over-representation of certain species. Would it be possible to calculate also the number of BGCs per genome considering only distinct species (selected based on Mash clustering) to provide a genus-wide overview of distribution, minimizing bias toward over-representation of certain species?**

*>> We have added a statement citing the average number of BGCs reported by earlier studies with an explanation of differences in the antiSMASH version used and the sampling bias of varying assembly qualities used.*

*Line 171: “To avoid sampling bias in number of genomes per species, we first calculated the means for each of the 808 species individually, which was then averaged to result in the mean of 29.9 BGCs in the Streptomyces genus.”*

*Line 177: “Previous genome mining studies of Streptomyces reported average BGCs in the genus to be 39.64* [*[7]*](https://paperpile.com/c/wrXCal/Oths)*, 44* [*[20]*](https://paperpile.com/c/wrXCal/2a3pR)*, and 31* [*[21]*](https://paperpile.com/c/wrXCal/cGXdD)*. The variation on predicted numbers is likely due to different versions of antiSMASH and the quality of genome assemblies used in the studies.”*

**- Would it be possible to specify the placement of clades I, II, and the O group previously described by McDonald and Currie (10.1128/mbio.00644-17) within the consensus tree from Figure S14? Does this phylogenetic analysis align with the core genome phylogenetic tree published in (https://urldefense.com/v3/__https://doi.org/10.3389/fmicb.2021.677558__;!!Mih3wA!E1XY1WDOJi_nHKsUIbda1h9pLfigvbRr-O_XI2P1HWpC2I17JaxBofMwm0M8FsAOA-x-jmngICl8ovR8$ ; or 10.1093/nar/gkac1076)?**

*>> It was difficult to find an accessible table for an accurate comparison to the clades I, II, O directly using the previous article by McDonald and Currie* [*[22]*](https://paperpile.com/c/wrXCal/rC6m)*. However, we manually checked most of the strains from the phylogenetic tree Figure in the article to map these clades onto the Mash-clusters presented in our study. We note that Mash-clusters M3 and M7 aligned with the genomes present in clade I. Mash-clusters M1, M2, and M4 mapped to clade II, whereas M5, M6, and unclustered groups mapped to the O (“others”) clade.*

*We were not able to retrieve the accessible tree file to accurately calculate the alignment with the core genome phylogenetic tree presented by Caicedo-Montoya et al 2021* [*[20]*](https://paperpile.com/c/wrXCal/2a3pR)*. However, we have included the de novo GTDBTk-based tree in our original comparative analysis, which is based on core 120 bacterial marker genes.*

**- Abstract - lines 39-40: Traditionally, the phrase 'acquisition of new genes' tends to evoke the idea of horizontal gene transfer rather than vertical inheritance. To better convey the concept of vertical inheritance, it might be more fitting to discuss 'BGC diversification'.**

*>> As recommended, we changed the phrasing to: “ …diversification of BGCs…”*

**- The term 'Mash' (first letter in uppercase followed by lowercase) should be used instead of 'MASH', in accordance with the nomenclature originally published in Genome Biology (DOI 10.1186/s13059-016-0997-x). This will also prevent any confusion with antiSMASH, which readers might naturally associate with BGC clusters.**

*>> We have replaced all occurrences of “MASH” with “Mash” in the text as well as figures.*

**- Figure 1: Would it be possible to revise the panel C to enhance the coherence of the classification? At first glance, some categories may seem to overlap or appear disparate, such 'NRPS' and 'NRPS-like'. I understand that the current classification follows traditional pipeline biosynthetic (sub)classes, but is there a method to extract the logic more effectively? For instance, grouping all NRPS and NRPS-like categories together or arranging single modules first, followed by double modules, then hybrids could enhance clarity. The current descending order of abundance of sub-categories makes it challenging to discern a clear message regarding biosynthetic classes (NRPS and related categories represented at least three points apart, as with PKS). Additionally, the sentence "Number of known hits against MIBIG vary depending on BGC type" should be removed from the panel, as it is more suited for inclusion in the main text rather than within the figure itself. Please include a list of abbreviations in the legend of this figure.**

*>> We have now used the broad categories such as PKS, NRPS, RiPP, Terpene, and other to annotate each BGC type. We extracted the broad categories for each protocluster from the antiSMASH output. The figure was reordered to include each of the single type categories, followed by hybrids. For example, three PKS BGCs were reported as seperate bars. The other PKS category included all other types of PKS and the hybrids with PKS types. The other PKS category would exclude hybrids with NRPS as this is represented by other bars.*

*As suggested, we have also removed the results highlighted from the figure panel and expanded the legend to include abbreviations for BGC types.*

**- Line 76: The list of comparative genomics studies in Streptomyces is not exhaustive (only 2 references cited). For instance, Lorenzi et al. (https://urldefense.com/v3/__https://doi.org/10.1099/mgen.0.000525__;!!Mih3wA!E1XY1WDOJi_nHKsUIbda1h9pLfigvbRr-O_XI2P1HWpC2I17JaxBofMwm0M8FsAOA-x-jmngIGKnbyvm$ ) reported around one thousand core genes. Studies from Nikolaidis et al. (https://urldefense.com/v3/__https://doi.org/10.1099/mgen.0.001028__;!!Mih3wA!E1XY1WDOJi_nHKsUIbda1h9pLfigvbRr-O_XI2P1HWpC2I17JaxBofMwm0M8FsAOA-x-jmngIMCt56eb$ ) or Belknap et al. (https://urldefense.com/v3/__https://www.nature.com/articles/s41598-020-58904-9__;!!Mih3wA!E1XY1WDOJi_nHKsUIbda1h9pLfigvbRr-O_XI2P1HWpC2I17JaxBofMwm0M8FsAOA-x-jmngIHmeJTAX$ ) may also be interesting to considered.**

*>> We thank the reviewer for suggesting additional references. All the suggested references are now included in lines 77 to 81.*

**- Family and genus names should be written in italics (https://urldefense.com/v3/__https://doi.org/10.1099/ijsem.0.005585__;!!Mih3wA!E1XY1WDOJi_nHKsUIbda1h9pLfigvbRr-O_XI2P1HWpC2I17JaxBofMwm0M8FsAOA-x-jmngICSaU6ZI$ ). Please write 'Streptomyces' or 'Streptomycetaceae' in italics at lines: 58, 93, 439, 440, etc. (and in the references).**

*>> Updated now at all suggested places.*

**- Line 203: "(previously designated" should not be written in italics.**

*>> Updated now.*

**- Lines 237 and 501: Perhaps the "_" isn't necessary.**

*>> Updated now.*

**- References need to be edited: references 4, 15 and 38 correspond to the same article (and/or its correction); likewise for 12 and 18.**

*>> Updated to correct citation by removing the redundant citations*

**- Data S1 legend: please replace 'selcted' by 'selected'.**

*>> Updated now.*

**- Figure S2 legend: please replace 'recnet' by 'recent'.**

*>> Updated now.*

**- Figure S14: the word 'color' is present twice.**

*>> Updated now.*

References:

[1. Nuhamunada M, Mohite OS, Phaneuf PV, Palsson BO, Weber T. BGCFlow: systematic pangenome workflow for the analysis of biosynthetic gene clusters across large genomic datasets. Nucleic Acids Res [Internet]. 2024; Available from:](http://paperpile.com/b/wrXCal/tLKd) <http://dx.doi.org/10.1093/nar/gkae314>

[2. Ondov BD, Treangen TJ, Melsted P, Mallonee AB, Bergman NH, Koren S, et al. Mash: fast genome and metagenome distance estimation using MinHash. Genome Biol. 2016;17:132.](http://paperpile.com/b/wrXCal/df7I)

[3. Hernández-Salmerón JE, Moreno-Hagelsieb G. FastANI, Mash and Dashing equally differentiate between species. PeerJ. 2022;10:e13784.](http://paperpile.com/b/wrXCal/EjeW)

[4. Jørgensen TS, Mohite OS, Sterndorff EB, Alvarez-Arevalo M, Blin K, Booth TJ, et al. A treasure trove of 1034 actinomycete genomes. Nucleic Acids Res [Internet]. 2024; Available from:](http://paperpile.com/b/wrXCal/rzd4) <http://dx.doi.org/10.1093/nar/gkae523>

[5. Omura S, Ikeda H, Ishikawa J, Hanamoto A, Takahashi C, Shinose M, et al. Genome sequence of an industrial microorganism Streptomyces avermitilis: deducing the ability of producing secondary metabolites. Proc Natl Acad Sci U S A. 2001;98:12215–20.](http://paperpile.com/b/wrXCal/xVCT)

[6. Bentley SD, Chater KF, Cerdeño-Tárraga A-M, Challis GL, Thomson NR, James KD, et al. Complete genome sequence of the model actinomycete Streptomyces coelicolor A3(2). Nature. 2002;417:141–7.](http://paperpile.com/b/wrXCal/hFMV)

[7. Belknap KC, Park CJ, Barth BM, Andam CP. Genome mining of biosynthetic and chemotherapeutic gene clusters in Streptomyces bacteria. Sci Rep. 2020;10:2003.](http://paperpile.com/b/wrXCal/Oths)

[8. Blin K, Shaw S, Augustijn HE, Reitz ZL, Biermann F, Alanjary M, et al. antiSMASH 7.0: new and improved predictions for detection, regulation, chemical structures and visualisation. Nucleic Acids Res. 2023;51:W46–50.](http://paperpile.com/b/wrXCal/fuHV)

[9. Navarro-Muñoz JC, Selem-Mojica N, Mullowney MW, Kautsar SA, Tryon JH, Parkinson EI, et al. A computational framework to explore large-scale biosynthetic diversity. Nat Chem Biol. 2020;16:60–8.](http://paperpile.com/b/wrXCal/fIxI)

[10. Kautsar SA, van der Hooft JJJ, de Ridder D, Medema MH. BiG-SLiCE: A highly scalable tool maps the diversity of 1.2 million biosynthetic gene clusters. Gigascience [Internet]. 2021;10. Available from:](http://paperpile.com/b/wrXCal/p2Dj) <http://dx.doi.org/10.1093/gigascience/giaa154>

[11. Blin K, Shaw S, Kautsar SA, Medema MH, Weber T. The antiSMASH database version 3: increased taxonomic coverage and new query features for modular enzymes. Nucleic Acids Res. 2021;49:D639–43.](http://paperpile.com/b/wrXCal/S8Z9)

[12. Gavriilidou A, Kautsar SA, Zaburannyi N, Krug D, Müller R, Medema MH, et al. Compendium of specialized metabolite biosynthetic diversity encoded in bacterial genomes. Nat Microbiol. 2022;7:726–35.](http://paperpile.com/b/wrXCal/kz2g)

[13. Steinke K, Mohite OS, Weber T, Kovács ÁT. Phylogenetic Distribution of Secondary Metabolites in the Bacillus subtilis Species Complex. mSystems [Internet]. 2021;6. Available from:](http://paperpile.com/b/wrXCal/x2jP) <http://dx.doi.org/10.1128/mSystems.00057-21>

[14. Mohite OS, Lloyd CJ, Monk JM, Weber T, Palsson BO. Pangenome analysis of Enterobacteria reveals richness of secondary metabolite gene clusters and their associated gene sets. Synth Syst Biotechnol. 2022;7:900–10.](http://paperpile.com/b/wrXCal/Hdit)

[15. Mira A, Martín-Cuadrado AB, D’Auria G, Rodríguez-Valera F. The bacterial pan-genome:a new paradigm in microbiology. Int Microbiol. 2010;13:45–57.](http://paperpile.com/b/wrXCal/L8rx)

[16. Passarelli-Araujo H, Franco GR, Venancio TM. Network analysis of ten thousand genomes shed light on Pseudomonas diversity and classification. Microbiol Res. 2022;254:126919.](http://paperpile.com/b/wrXCal/BKCm)

[17. Abram K, Udaondo Z, Bleker C, Wanchai V, Wassenaar TM, Robeson MS 2nd, et al. Mash-based analyses of Escherichia coli genomes reveal 14 distinct phylogroups. Commun Biol. 2021;4:117.](http://paperpile.com/b/wrXCal/RImU)

[18. Myronovskyi M, Rosenkränzer B, Nadmid S, Pujic P, Normand P, Luzhetskyy A. Generation of a cluster-free Streptomyces albus chassis strains for improved heterologous expression of secondary metabolite clusters. Metab Eng. 2018;49:316–24.](http://paperpile.com/b/wrXCal/FLa2A)

[19. Saati-Santamaría Z. Global Map of Specialized Metabolites Encoded in Prokaryotic Plasmids. Microbiol Spectr. 2023;11:e0152323.](http://paperpile.com/b/wrXCal/5Z8w)

[20. Caicedo-Montoya C, Manzo-Ruiz M, Ríos-Estepa R. Pan-Genome of the Genus Streptomyces and Prioritization of Biosynthetic Gene Clusters With Potential to Produce Antibiotic Compounds. Front Microbiol. 2021;12:677558.](http://paperpile.com/b/wrXCal/2a3pR)

[21. Otani H, Udwary DW, Mouncey NJ. Comparative and pangenomic analysis of the genus Streptomyces. Sci Rep. 2022;12:18909.](http://paperpile.com/b/wrXCal/cGXdD)

[22. McDonald BR, Currie CR. Lateral Gene Transfer Dynamics in the Ancient Bacterial Genus. MBio [Internet]. 2017;8. Available from:](http://paperpile.com/b/wrXCal/rC6m) <http://dx.doi.org/10.1128/mBio.00644-17>

**2^nd^ round**

**Reviewer 1**

The author's have done a nice job responding to the queries and improving the quality of the manuscript. No further comments from me.

**Reviewer 2**

The revisions bring substantial value to the article by clarifying the scope of the analyses and addressing the limitations of the methods used, as well as their significance and how they compare with other studies in the field. Additionally, the integration of sequencing data from a recent panel of sequenced genomes offers a meaningful enhancement to our understanding of Streptomyces genome biology.
I appreciate the inclusion of the M6 cluster analysis, which deepens our insights by expanding the range of species examined.
Throughout the abstract and text, the article supports the idea that vertical heredity is a primary driver in the diversification of biosynthetic gene clusters (BGCs). While precise quantification of vertical versus horizontal transfer effects was not feasible within this study's scope—likely due to methodological complexities and the substantial time required— the findings offer valuable discussion points. They suggest that the impact of horizontal transfer on BGC diversification may have been overestimated. This article stands to make a significant contribution to this area of research.

Minor remarks:
Reference 20: 124 genomes were included, not 125.
Please italicise the genus Streptomyces (in sentences added during revision this is sometimes missing - e.g. line 94, 173, 177, 531, etc.) or species (line 439).
Lines 219 and 898: please use "Mash" instead of "MASH."
